# Aluminium und seine schwerlöslichen Verbindungen: MAK-Begründung, Nachtrag

**DOI:** 10.34865/mb742990verda10_3ad

**Published:** 2025-09-29

**Authors:** Andrea Hartwig, MAK Commission

**Affiliations:** 1 Institut für Angewandte Biowissenschaften. Abteilung Lebensmittelchemie und Toxikologie. Karlsruher Institut für Technologie (KIT) Adenauerring 20a, Geb. 50.41 76131 Karlsruhe Deutschland; 2 Ständige Senatskommission zur Prüfung gesundheitsschädlicher Arbeitsstoffe. Deutsche Forschungsgemeinschaft, Kennedyallee 40, 53175 Bonn, Deutschland. Weitere Informationen: Ständige Senatskommission zur Prüfung gesundheitsschädlicher Arbeitsstoffe | DFG

**Keywords:** Aluminium, schwerlösliche Aluminiumverbindungen, Lunge, Entzündung, Neurotoxizität, MAK-Wert, maximale Arbeitsplatzkonzentration, Spitzenbegrenzung, Kanzerogenität

## Abstract

The German Senate Commission for the Investigation of Health Hazards of Chemical Compounds in the Work Area (MAK Commission) summarized and evaluated the data for aluminium and its poorly soluble compounds to derive an occupational exposure limit value (maximum concentration at the workplace, MAK value) considering all toxicological end points. Relevant studies were identified from a literature search and also unpublished study reports were used. In occupational studies, aluminium causes lung toxicity for which no NOAEC could be established. In a 28-day study with rats, signs of pulmonary inflammation were detected in the bronchoalveolar lavage fluid, the lungs contained enlarged and foamy macrophages and the elimination half-life in the lungs was increased after exposure to aluminium oxyhydroxide at 11.03 to 12.29 mg Al/m^3^. Based on the NOAEC of 1.18 to 1.32 mg Al/m^3^, the MAK value for the respirable fraction (R fraction) of poorly soluble aluminium compounds such as aluminium oxide, aluminium hydroxide and aluminium oxyhydroxide has been set to 0.05 mg/m^3^. No neurotoxicity was observed in welders at an aluminium concentration of 0.25–0.5 mg Al/m^3^ for the R fraction. At an exposure to this concentration as inhalable fraction (10 m^3^/day), the amount of bioavailable aluminium (max. 1%) excreted daily with the urine corresponds to the Biological Tolerance Value (50 µg Al/g creatinine). The MAK value for the inhalable fraction (I fraction) has therefore been set to 0.5 mg Al/m³. Aluminium and its poorly soluble compounds are not acutely irritating and have thus been assigned to Peak Limitation Category II. Due to the long half-life in humans, an excursion factor of 8 has been set for both the R fraction and the I fraction. The increased elimination half-life in the lungs is a clear sign of particle overload which may induce lung carcinogenicity at higher concentrations. Therefore, aluminium and its poorly soluble compounds have been classified in Carcinogen Category 4. Aluminium and its poorly soluble compounds are not mutagenic. The results of several indicator tests suggest that aluminium and its poorly soluble compounds have DNA damaging potential, but valid in vivo tests did not find clastogenic or aneugenic effects. In the absence of inhalation developmental toxicity studies in animals, aluminium and its poorly soluble compounds have been assigned to Pregnancy Risk Group D. As absorption through the skin in toxicologically relevant amounts was not observed even in the case of soluble aluminium compounds, this is also not assumed to occur with aluminium and its poorly soluble compounds. A sensitizing potential is not expected based on the available data.

**Table:** 

**MAK-Wert (2024)**	**0,05 mg Al/m³ A** **0,5 mg Al/m³ E**
**Spitzenbegrenzung (2024)**	**Kategorie II, Überschreitungsfaktor 8 **
	
**Hautresorption **	**–**
**Sensibilisierende Wirkung **	**–**
**Krebserzeugende Wirkung (2024)**	**Kategorie 4^a)^**
**Fruchtschädigende Wirkung (2024)**	**Gruppe D**
**Keimzellmutagene Wirkung **	**–**
	
**BAT-Wert (2017)**	**50 µg Aluminium/g Kreatinin**
**BAR (2018)**	**15 µg Aluminium/g Kreatinin**

^a)^ aufgrund von Partikelüberladungseffekt in der Lunge

Seit der Begründung (Henschler 1987) und den Nachträgen (Greim 2007; Hartwig und MAK Commission 2019) sind neue Studien zu Aluminium und seinen schwerlöslichen Verbindungen publiziert worden, was eine Neubewertung aller Endpunkte erforderlich macht.

Dieser Nachtrag gilt für Aluminium und seine schwerlöslichen Verbindungen (physikalisch-chemische Daten, siehe Tabelle 1), inklusive ultrafeiner Partikel. Zitierte unveröffentlichte toxikologische Studien von Firmen wurden der Kommission zur Verfügung gestellt. Für das unlösliche α-Aluminiumoxid (Korund) (Hartwig und MAK Commission 2019) gilt dieser Nachtrag nicht. Lösliche Aluminiumverbindungen werden in einer separaten Begründung bewertet (Hartwig und MAK Commission 2025).

Als schwerlöslich werden solche Aluminiumverbindungen angesehen, die nicht mit dem von der Kommission publizierten Verfahren (Rosenberger et al. 2014), welches derzeit reevaluiert wird, in Lösung gebracht werden können. Für nicht bioverfügbare Aluminiumverbindungen, so auch für das unlösliche Korund (Hartwig und MAK Commission 2019) gilt der Allgemeine Staubgrenzwert für granuläre biobeständige Stäube (GBS) (siehe Hartwig 2012). Eine Aluminiumverbindung gilt als nicht bioverfügbar, wenn bei Exposition gegen diese Verbindung der Biologische-Arbeitsstoff-Referenzwert (BAR) für Aluminium (15 µg Al/g Kreatinin) (Klotz et al. 2019) nicht überschritten wird.

Die Referenzwerte des Umweltbundesamts für die Allgemeinbevölkerung liegen bei < 5 µg Al/l Blutserum und bei < 15 µg Al/l Urin (HBM-Kommission 1998).

**Tab. 1:** Physikalisch-chemische Parameter der in diesem Nachtrag bewerteten schwerlöslichen Aluminiumverbindungen

**Verbindung** **CAS-Nr.**	**Formel**	**Molmasse** **[g/mol]**	**Smp.** **[° C]**	**Sdp.** **[° C]**	**Dichte ** **[g/cm^3^]**	**Löslichkeit in Wasser** **im Sauren/Alkalischen**	**Literatur**
Aluminium [7429-90-5]	Al	26,98	660	2450	2,7	unlöslich löslich im Sauren und Alkalischen	ECHA 2023 a; NEG und DECOS 2011
Aluminiumhydroxid [21645-51-2]	Al(OH)_3_	78,01	300	2980	2,42	unlöslich löslich im Sauren	ECHA 2023 b; NEG und DECOS 2011
Aluminiumoxid [1344-28-1] γ-Modifikation δ-Modifikation	Al_2_O_3_	101,94	2054	2977	3,99	0,73 mg/l (ultrafein) < 10 µg/l (mikroskalig) etwas löslich im Alkalischen	Avramescu et al. 2022; ECHA 2023 c; NEG und DECOS 2011
Aluminiumoxyhydroxid [1318-23-6; 24623-77-6] γ-Modifikation	AlOOH	59,99	k. A.	k. A.	k. A.	k. A.k. A.	

k. A.: keine Angabe; Sdp.: Siedepunkt; Smp.: Schmelzpunkt

## 1 Allgemeiner Wirkungscharakter

In der bronchoalveolären Lavageflüssigkeit (BALF) von Wistar-Ratten sind nach vierwöchiger Inhalation von ultrafeinem **Aluminiumoxyhydroxid** ab 28 mg/m^3^ die Werte von Entzündungsmarkern erhöht. Diese Konzentration führt in der Lunge zu einem Partikelüberladungseffekt, der eine lungenkanzerogene Wirkung haben kann. Schwerlösliche Aluminiumverbindungen rufen bei exponierten Arbeitern in Konzentrationen von 4,5–6,8 mg/m^3^ präklinische neurotoxische Effekte durch Al^3+^-Ionen hervor. Für elementares Aluminium sowie schwerlösliche Aluminiumverbindungen lassen sich vereinzelt DNA-schädigende Wirkungen in Indikatortests beobachten. Untersuchungen zur Induktion von Chromosomenaberrationen und Mikronuklei geben jedoch keine Hinweise auf eine direkte Genotoxizität. Schwerlösliche Aluminiumverbindungen sind nicht haut- oder augenreizend. Es liegen keine Studien zur Entwicklungstoxizität nach inhalativer Exposition vor. Keine entwicklungs- und maternaltoxischen Effekte haben sich für **Aluminiumhydroxid**, der einzigen untersuchten Verbindung, bis zu den höchsten Dosierungen von 266 bzw. 100 mg Aluminium/kg KG und Tag bei Ratten und Mäusen ergeben. Es liegen nur wenige Hinweise auf eine allergene Wirkung von Aluminium und seinen schwerlöslichen Verbindungen vor.

## 2 Wirkungsmechanismus

Für Aluminium ist keine physiologische Funktion im menschlichen Organismus bekannt, wenngleich es Hinweise auf Interaktionen mit über 200 biologisch wichtigen Prozessen gibt (Kawahara und Kato-Negishi 2011).

Die Toxizität schwerlöslicher Aluminiumverbindungen in der Lunge wird durch den Partikelüberladungseffekt zusammen mit der Wirkung freigesetzter Aluminiumionen ausgelöst:

Aluminiumionen liegen nur in der Oxidationsstufe Al^3+^ vor und besitzen eine hohe Affinität für negativ geladene Sauerstoffliganden. So geht das Aluminiumion mit anorganischen und organischen Phosphaten, Carboxylaten und deprotonierten Hydroxylgruppen starke Bindungen ein. Konkret kann das Aluminiumion an Phosphatgruppen der DNA, RNA und Nukleosid-Di- und -Triphosphate binden. Aufgrund der stark positiven Ladung sowie des im Vergleich mit anderen Metallionen wie Ca^2+^, Zn^2+^ und Na^+ ^relativ kleinen Ionenradius kann es an Aminosäuren wie Histidin, Tyrosin, Arginin und phosphorylierte Aminosäuren binden und diese quervernetzen (Kawahara und Kato-Negishi 2011, siehe auch Abschnitt 2.3.4, Hartwig und MAK Commission 2025).

### 2.1 Partikelüberladungseffekt und Kanzerogenität

In einer 28-Tage-Inhalationsstudie wurden männliche Wistar-Ratten gegen Aerosole von zwei ultrafeinen **Aluminiumoxyhydroxid**-Chargen (Primärpartikeldurchmesser 10 oder 40 nm, MMAD (massenmedianer aerodynamischer Durchmesser): 1,7 μm bzw. 0,6 μm) in Konzentrationen bis zu 28 mg/m^3^ nur über die Nase exponiert. In der höchsten Konzentrationsgruppe zeigten sich Entzündungsreaktionen in der Lunge (Bayer Schering Pharma AG 2008, 2009; Pauluhn 2009 b, siehe Abschnitt 5.2.1), verringerte Lungenclearance und erhöhte Aluminiumkonzentrationen im Urin. Die Entzündungsreaktionen in der Lunge können daher sowohl auf den Überladungseffekt, als auch auf die Toxizität des freigesetzten Aluminiumions zurückzuführen sein.

Der mögliche Wirkungsmechanismus der ultrafeinen Aluminiumoxyhydroxid-Partikel wurde detailliert analysiert:

Nach Inhalation von agglomerierten ultrafeinen Partikeln (Agglomerate ≥ 0,5 µm) ist die Phagozytose der zu erwartende Hauptaufnahmeweg in Makrophagen. Kleinere Partikel werden vorwiegend über Endozytose aufgenommen (Pauluhn 2009 a).

Die Eliminations-Halbwertszeit (HWZ) für Aluminiumoxyhydroxid blieb in der 28-Tage-Inhalationsstudie bis zur mittleren Konzentration von 3 mg/m^3^ unter 60 Tagen (Normalwert für Ratten; Donaldson et al. 2008; Stöber und McClellan 1997), überschritt diese allerdings deutlich bei der höchsten Konzentration von 28 mg/m^3^ bei den 10-nm-Partikeln und den 40-nm-Partikeln (177 Tage bzw. 94 Tage) (Bayer Schering Pharma AG 2008, 2009; Pauluhn 2009 b), was einen Überladungseffekt in der Lunge anzeigt (Hartwig 2012). Bei der mittleren Konzentration trat zudem keine Erhöhung der neutrophilen Granulozyten in der BALF auf. Somit lag kein Hinweis auf ein Entzündungsgeschehen vor, solange die Partikellast in der Lunge die normale Makrophagen-Clearance nicht behinderte (Pauluhn 2009 c). Diese Interpretation entspricht dem Konzept der pulmonalen Partikelüberlastung und Toxizität in der Lunge von Oberdörster (1995) und Oberdörster et al. (2005). Eine längere Eliminations-HWZ setzt ein, wenn ein Lungenmakrophage ein Partikelvolumen von ≥ 60 μm^3^ akkumuliert und dadurch immobilisiert wird. Dieser Wert entspricht einer Partikelbelastung der Lunge von ca. 1 mg/g (Morrow 1988, 1992). Passend dazu zeigte sich in der Studie von Pauluhn (2009 b) eine verzögerte Elimination bei einer Partikelkonzentration von ca. 2 mg Partikel/g Lunge. Die geringere Agglomerat-Dichte von nanoskaligen Primärpartikeln führt dazu, dass sie bei geringeren Massen zu einer volumetrischen Überladung führen, als wenn sie als mikroskalige Primärpartikel vorliegen (Pauluhn 2009 c).

Die längere Eliminations-HWZ der 10-nm-Partikel (177 Tage für Lungengewebe und BAL-Zellen) deutet auf eine größere Potenz für Partikelakkumulation über längere Expositionszeiträume hin als bei 40-nm-Partikeln (94 Tage für Lungengewebe und BAL-Zellen) (Pauluhn 2009 b). Für die 40-nm-Partikel, die einen kleineren MMAD aufwiesen (0,6 μm), konnte eine höhere Lungenbelastung beobachtet werden im Vergleich zu den 10-nm-Partikeln, die einen größeren MMAD besaßen (1,7 μm). Demnach hängt die Lungenbelastung mehr vom MMAD der agglomerierten Partikel ab, der den Ablagerungsort und das Ausmaß der Deposition in den Atemwegen bestimmt, als von der Größe der Primärpartikel (Pauluhn 2009 c). Warum die 40-nm-Partikel trotz höherer Lungenbeladung eine geringere HWZ aufwiesen, ist unklar.

In einem Review wurden die molekularen Mechanismen, die nach Inhalation von ultrafeinen Aluminiumoxidpartikeln in der Lunge ausgelöst werden, beschrieben. Zu den proinflammatorischen Mechanismen, die durch eine Inhalation von Aluminiumoxid (γ-, δ-Modifikation) in Ratten ausgelöst werden, zählt der Anstieg von Neutrophilen, Lymphozyten und Makrophagen in der BALF in Verbindung mit der Sekretion von proinflammatorischen Cytokinen (IL (Interleukin)-6, IL-1β, TNF-α (Tumornekrosefaktor α) und MIP-2 (Macrophage inflammatory protein) und LDH (Lactatdehydrogenase), was eine Schädigung der alveolokapillären Barriere anzeigt (Dekali et al. 2022; siehe auch Abschnitt 5.2.1.2, Tabelle 7).

Der zuvor beschriebene Überladungseffekt in der Lunge kann zu Tumoren führen. Detailliert beschrieben ist dieser Wirkmechanismus nach chronischer inhalativer Exposition gegen unlösliches Korund, was als GBS ohne stoffspezifische Toxizität zur Akkumulation von Staubpartikeln in der Lunge und den Lymphknoten, zur Beeinträchtigung der Lungenfunktion, zur Überlastung der Clearance, zu entzündlichen Veränderungen der Lunge, zur Fibrosierung und zur Tumorentstehung führen kann (Hartwig 2012; Hartwig und MAK Commission 2019). Die Untersuchungen von Pauluhn (2009 a, b, c) mit schwerlöslichen, ultrafeinen Aluminiumoxyhydroxid-Partikeln zeigen deutlich einen partikelbedingten Überladungseffekt, weshalb ein Wirkungsmechanismus ähnlich dem von GBS angenommen wird und eine Tumorentstehung in der Lunge möglich ist.

**Fazit Partikelüberladungseffekt und Kanzerogenität:** Für **Aluminiumoxyhydroxid**partikel ist anhand der deutlichen Zunahme der Eliminations-HWZ (> 60 Tage) und der Induktion von Entzündungsreaktionen in der Rattenlunge ein Partikelüberladungseffekt nachgewiesen. Zusätzlich muss mit einer Wirkung von freien Aluminiumionen (siehe Abschnitt 2.2 und 2.3) gerechnet werden. Welcher Effekt bei chronischer Exposition führend ist, kann anhand der vorliegenden Daten nicht geklärt werden. Da der mit Aluminiumoxyhydroxid nachgewiesene Überladungseffekt der Wirkungsweise von GBS gleicht, kann eine Tumorbildung in der Lunge durch schwerlösliche Aluminiumverbindungen bei ausreichend hoher Konzentration möglich sein.

Wegen der Wirkung von freien Aluminiumionen kann der Allgemeine Staubgrenzwert (A-Fraktion) GBS nicht angewendet werden („Der Allgemeine Staubgrenzwert ist nicht zur Anwendung gekommen, wenn genotoxische, krebserzeugende, fibrogene, allergisierende oder sonstige systemisch-toxische Wirkungen des Staubes zu erwarten waren“ (Hartwig 2012)).

### 2.2 Neurotoxizität

Eine Vielzahl von Studien deuten auf durch Aluminiumionen ausgelöste mitochondriale Dysfunktion, veränderten Energiestoffwechsel, oxidativen Stress, Störung der Calciumhomöostase, Entzündungsreaktionen und Apoptose hin. Derartige Effekte wurden auch im Gehirn nachgewiesen. Aluminiumionen können Marker für neurodegenerative Erkrankungen wie Tau-Proteine, β-Amyloid-Proteine und intrazelluläre Neurofibrillenbündel (NFT) beeinflussen, die synaptische Plastizität und die Signalweiterleitung beeinträchtigen und scheinen einen signifikanten Einfluss auf die Neurotransmission durch Glutamat, Gamma-Aminobuttersäure, Acetylcholin, Serotonin und Dopamin zu haben. Details zu diesen Untersuchungen sind in der Begründung zu löslichen Aluminiumverbindungen aufgeführt (Hartwig und MAK Commission 2025). Untersuchungen mit elementarem Aluminium und schwerlöslichen Aluminiumverbindungen sind nachfolgend dargestellt.

#### 2.2.1 Humandaten

In einer Studie wurde die Tau-Protein-Expression in peripheren Blutlymphozyten an 66 sich im Ruhestand befindenden exponierten Beschäftigten einer Aluminiumgießerei im Vergleich zu nicht exponierten Kontrollpersonen untersucht. In der exponierten Gruppe zeigte sich eine statistisch signifikante Abnahme der kognitiven Funktion im Minimal-Mental-Status-Test (MMSE) sowie ein statistisch signifikant erhöhter Gehalt an p-Tau 181 und p-Tau 231 (Lu et al. 2014). Bei 85 Arbeitern in der Aluminiumproduktion mit leichter Verminderung der kognitiven Funktion im MMSE- und Uhrentest (Clock-Drawing-Test, CDT; MMSE < 24; CDT < 3) waren im Vergleich zu 85 angepassten Kontrollpersonen der Serumgehalt an Aluminium signifikant erhöht (ca. 80 im Vgl. zu 40 µg Al/l) und die Genexpression von Phosphoinositid-3-Kinasen (PI3K, kodiert von PIK3CA), *AKT* und mammalian target of rapamycin (*MTOR*)1 statistisch signifikant vermindert (Shang et al. 2020). In einer Querschnittsstudie ließ sich an 75 Gießereiarbeitern eine statistisch signifikante Abnahme kognitiver Funktionen anhand des Montreal-Cognitive-Assessment (MocA)-Tests und eine Zunahme des Tau-Proteins im Serum im Vergleich zu 75 Kontrollpersonen feststellen (Mohammed et al. 2020). Im Serum der exponierten Arbeiter waren neben Aluminium allerdings auch die Blei- und Mangankonzentrationen erhöht, sodass nicht klar ist, ob der Effekt auf Aluminium zurückgeführt werden kann.

#### 2.2.2 Tierdaten

Männliche NMRI-Mäuse erhielten fünf Tage lang 5 oder 10 mg ultrafeines **Aluminiumoxid**/kg KG und Tag per Schlundsonde. Im Hippocampus waren die Proteine p-p38 und p-ERK (extracellular-signal regulated kinases), die Verhältnisse von p-p38/p38, p-ERK/GAPDH (Glycerinaldehyd-3-phosphat-Dehydrogenase) und p-ERK/ERK sowie die Protein-Spaltung durch Caspase-3, ein Biomarker der Apoptose, erhöht. Die Untersuchung der kognitiven Erinnerung im novel-object recognition (NOR)-Test zeigte keinen signifikanten Unterschied im Vergleich zur Kontrolle (Mehrbeheshti et al. 2022).

#### 2.2.3 Fazit Neurotoxizität

Die Neurotoxizität von Aluminiumionen kann durch eine Vielzahl von Interaktionen mit molekularen Mechanismen begründet werden, welche ausführlich in der Begründung für lösliche Aluminiumionen beschrieben sind (Hartwig und MAK Commission 2025). Auch schwerlösliche Aluminiumverbindungen beeinflussen wiederum durch die Freisetzung von Aluminiumionen das Tau-Protein sowie die Genexpression von *PIK3CA* (kodiert für PI3K), *AKT* und *MTOR* bei Exponierten. An der Maus wurde die Veränderung von Proteinen des MAP (mitogen-activated protein)-Kinasen-Wegs sowie der Aktivität des Apoptosemarkers Caspase-3 beobachtet.

### 2.3 Genotoxizität 

Die genotoxische Wirkung der schwerlöslichen Aluminiumverbindungen beruht auf den Mechanismen wie sie auch für lösliche Aluminiumverbindungen (Hartwig und MAK Commission 2025) beschrieben sind. Im Folgenden sind die Studien, die mit elementarem Aluminium oder schwerlöslichen Aluminiumverbindungen durchgeführt wurden, dargestellt.

#### 2.3.1 Humandaten

Bei Beschäftigten in der Aluminiumindustrie kam es zu einer Induktion von reaktiven Sauerstoffspezies (ROS), die jedoch aufgrund der Mischexposition nicht eindeutig auf Aluminium zurückführbar ist (Bulat et al. 2008).

#### 2.3.2 In-vitro-Studien

In humanen Lymphozyten ergab die 24-stündige Inkubation mit ultrafeinem **Aluminiumoxid** (< 50 nm) eine statistisch signifikante Zunahme an ROS und eine Abnahme des Glutathion (GSH)-Gehaltes bei 100 µg/ml sowie eine Steigerung der Katalase-Aktivität ab 75 µg/ml (Rajiv et al. 2016).

Lungenzellen des Chinesischen Hamsters (CHL-Zellen) wurden mit mikroskaligem (10 µm) sowie ultrafeinem Aluminiumoxid (13 und 50 nm) in Konzentrationen von 15, 30 und 60 µg/ml inkubiert und GSH, Superoxiddismutase (SOD), Malondialdehyd (MDA) und die antioxidative Kapazität (total antioxidant capacity, TAC) nach 12, 24 und 48 Stunden untersucht. Während MDA anstieg, nahmen GSH, SOD sowie die TAC ab. Zwischen den ultrafeinen und mikroskaligen Partikeln zeigte sich nur ein marginaler Unterschied. Zytotoxizität trat ab 12,5 µg/ml für alle Substanzen auf (siehe Abschnitt 5.6.1) (Zhang et al. 2017).

In HepG2-Zellen ließ sich nach Inkubation mit ultrafeinem Aluminiumoxid (30–60 nm) nach 48 Stunden ab 50 µg/ml und nach 24 Stunden ab 150 µg/ml eine Zunahme von ROS und SOD sowie eine Abnahme an GSH feststellen. Die Induktion von Apoptose wurde als erhöhte Caspase-3-Aktivität nach 48 Stunden ab 150 µg/ml sowie nach 24 Stunden bei 450 µg/ml beobachtet (Alarifi et al. 2015).

#### 2.3.3 Tierdaten

Die toxischen Effekte von **ultrafeinen** und **mikroskaligen Aluminium**partikeln auf das Rattengehirn wurden nach 28-tägiger intraperitonealer Gabe von 2, 4 oder 8 mg/kg KG und Tag untersucht. Anhand der Marker für oxidativen Stress (GSH, MDA) ließen sich in den Gehirnzellen nach Gabe des ultrafeinen verglichen mit mikroskaligem Aluminium ausgeprägtere oxidative Schäden erkennen, was durch die vermutete höhere Aufnahme der ultrafeinen Partikel ins Gehirn erklärt wurde (Mirshafa et al. 2018).

Nach oraler Gabe von 53 mg Al/kg KG und Tag als ultrafeines **Aluminiumoxid** für 75 Tage ließ sich eine Induktion von ROS sowie eine Modulation antioxidativer Marker wie die Verminderung der SOD-Aktivität im Serum von Wistar-Ratten beobachten (Naji et al. 2023). Mit der gleichen Expositionszeit und Dosis an ultrafeinem Aluminiumoxid zeigte sich eine Zunahme von MDA sowie eine Abnahme der Aktivitäten der SOD und Glutathionperoxidase (GPx) in der Leber von Sprague-Dawley-Ratten (Abo-EL-Sooud et al. 2023). Bei BALB/c-Mäusen waren nach 6-monatiger oraler Gabe von mikroskaligem **Aluminiumhydroxid** (3 mg Al/kg KG und Tag) in der Lunge die SOD- und Katalase (CAT)-Enzymaktivität reduziert (de Souza et al. 2023). Bei Swiss-Albino-Mäusen waren nach fünftägiger Gabe von **ultrafeinem Aluminiumoxid** Veränderungen von SOD und CAT sowie Lipidperoxidation und GSH in Gehirn, Leber, Milz, Nieren und Hoden zu beobachten (De et al. 2020, Details siehe Abschnitt 5.2.2).

Bei 28-tägiger Schlundsondengabe von ultrafeinen Aluminiumoxidpartikeln hatten ICR-Mäuse veränderte Konzentrationen von Kupfer, Zink, Mangan und Eisen in verschiedenen Geweben. Die Autoren sehen dies als Hinweis einer antioxidativen Reaktion auf Aluminium-induzierte ROS (Park et al. 2017). Es wurden keine antioxidativen Enzyme gemessen und nicht untersucht, ob die gemessenen Metallionen aus antioxidativen Enzymen stammen. Es ist somit nicht klar, ob die Veränderung der Metallionenkonzentrationen einen adversen Effekt darstellt. Weiterhin waren die Metallionenkonzentrationen auch in Organen verändert, in denen keine Zunahme an Aluminiumoxidpartikeln beobachtet wurde (wie etwa in der Leber, siehe Abschnitt 3.1.6), weshalb ein Rückschluss auf einen Effekt durch Aluminium nicht plausibel ist.

Sechs weibliche Wistar-Albino-Ratten pro Gruppe wurden per Schlundsonde mit 0; 0,5 oder 50 mg ultrafeinem Aluminiumoxid (γ-Modifikation)/kg KG und Tag für 14 Tage behandelt. Es ließ sich in den Nieren ab 5 mg/kg KG und Tag eine Abnahme der Gesamt-ATPase-Aktivität sowie nur bei 5 mg/kg KG und Tag eine Verminderung der Na-K-ATPase-Aktivität feststellen. Im Gehirn war eine Abnahme der Na-K-ATPase- und Acetylcholinesterase-Aktivität ab 0,5 mg/kg KG und Tag sowie der Gesamt-ATPase-Aktivität ab 5 mg/kg KG und Tag zu beobachten. Die ultrafeinen Aluminiumoxidpartikel akkumulierten in Gehirn, Leber und Nieren (Canli et al. 2019).

#### 2.3.4 Fazit Genotoxizität 

Die durch das Aluminiumion ausgelöste Genotoxizität kann durch oxidativen Stress erklärt werden. Die Untersuchungen mit mikroskaligen und ultrafeinen Aluminiumverbindungen zeigen eine Verminderung der Aktivität und der Proteinmenge antioxidativer Enzyme, eine Induktion der Lipidperoxidation sowie eine Zunahme an ROS. Weiterhin ist die Bildung von DNA-Schäden durch elementares Aluminium sowie schwerlösliche Aluminiumverbindungen zu nennen (siehe Abschnitt 5.6), welche durch ROS induziert werden können.

## 3 Toxikokinetik und Metabolismus 

Das Löslichkeitsverhalten von ultrafeinen und mikroskaligen Metalloxiden, darunter auch **Aluminiumoxid** (γ-Modifikation), wurde in folgenden Flüssigkeiten in einem Orbitalschüttler bei 37 °C untersucht: Wasser (pH: 6,4 ± 0,5), Zellkulturmedium (DMEM + 2 % fetales Kälberserum, pH: 7,6 ± 0,1), simulierte Phagolysosomalflüssigkeit (PSF, pH: 4,5 ± 0,02) und simulierte Lungenflüssigkeit (Gamble's solution, pH: 7,5 ± 0,1). Nach 48 Stunden zeigte sich bei einer Konzentration von 100 mg Aluminiumoxid/l eine statistisch signifikant höhere Löslichkeit (% der Ausgangskonzentration) der ultrafeinen Partikel im Vergleich zu den mikroskaligen Partikeln in Wasser (1,38 % (0,73 mg/l) bzw. < 0,01 % (< 10 µg/l)) und DMEM (0,73 % bzw. 0,02 %). In DMEM war die prozentuale Löslichkeit von ultrafeinem Aluminiumoxid bei 10 mg/l statistisch signifikant höher als bei 100 mg/l (1,11 bzw. 0,73 %). Die Untersuchung in PSF und simulierter Lungenflüssigkeit konnte für die Aluminiumoxidverbindungen nicht durchgeführt werden, was auf Wiederfindungsverluste während der Partikelsedimentation durch Zentrifugation erklärt wurde. Als Separationsmethode wurde in dieser Studie Zentrifugation gewählt, um eine Artefaktbildung durch Ultrafiltration zu vermeiden. Die Autoren vermuten allerdings auch bei dieser Methode eine Artefaktbildung, da Aluminiumionen an Phosphate in Proteinen und den Medien binden und dadurch eine Präzipitation des Aluminiumphosphates erfolgt, wodurch die Löslichkeiten tatsächlich höher sind als gemessen (Avramescu et al. 2022).

### 3.1 Aufnahme, Verteilung, Ausscheidung

#### 3.1.1 Inhalative Aufnahme 

##### 3.1.1.1 Humandaten

Aluminium-Schweißer haben erhöhte Aluminium-Werte im Urin (siehe Tabelle 4, Abschnitt 4.2), womit eine inhalative Aufnahme gezeigt ist.

Bei zwei männlichen Freiwilligen, welche radioaktiv markiertes **Aluminiumoxid** (^26^Al_2_O_3_, alveolengängig) einmalig 20 Minuten lang inhalierten, waren über einen Beobachtungszeitraum von 1000 Tagen von der anfangs in der Lunge deponierten Aluminiummenge ca. 1,9 % systemisch verfügbar. Dies stimmt mit den bei Arbeitern der Aluminiumindustrie berechneten Werten für eine Aufnahme von ca. 1,5–2 % überein (Greim 2007; Priest 2004). Neue Untersuchungen am Menschen zu einzelnen schwerlöslichen Aluminiumverbindungen liegen nicht vor.

Anhand ihrer durchschnittlichen Aluminiumexposition wurden 84 Beschäftigte einer Aluminiumschmelzerei in drei Gruppen eingeteilt: 0,036 mg Al/m³ (niedrig; 33 Personen), 0,35 mg Al/m³ (mittel; zwölf Personen) und 1,47 mg Al/m³ (hoch; 39 Personen). Die Messungen wurden stationär in Atemhöhe durchgeführt. Die durchschnittlichen Aluminiumkonzentrationen im Serum der Beschäftigten betrugen 4,10 ± 2,55 µg/l (niedrig); 4,85 ± 3,26 µg/l (mittel) und 7,15 ± 3,03 µg/l (hoch), bei den 48 Kontrollpersonen 4,76 ± 1,63 µg/l. Die durchschnittlichen Aluminiumkonzentrationen im Urin der drei exponierten Personengruppen betrugen 33,2 ± 19,0 µg/l (niedrig); 67,0 ± 34,0 µg/l (mittel) und 133,3 ± 63,4 µg/l (hoch), bei 59 Kontrollpersonen 23,7 ± 11,6 µg/l (Röllin et al. 1996). Bei der Untersuchung wurde nicht ermittelt, welche Aluminiumverbindung(en) die Beschäftigten inhalierten.

In fünf Betrieben für Recycling von elektronischen Geräten wurde u. a. Aluminium in der Arbeitsplatzluft und im Urin von 51 Beschäftigten sowie 20 Kontrollpersonen bestimmt. Die Messung der alveolengängigen und einatembaren Fraktion ergab mediane Werte von 1,8 µg/m³ (1,4–2,1) bzw. 6,85 µg/m³ (2,0–85,0) für die personenbezogene Messung, sowie 1,50 µg/m³ (0,75–4,5) bzw. 3,70 µg/m³ (0,89–15,0) für die stationäre Messung. Im Urin waren die Median-Aluminiumgehalte 4,10 µg/l (1,5–21,2) bzw. 3,40 µg/g Kreatinin (1,5–21,2) bei den Beschäftigten und 3,60 µg/l (1,5–8,7) bzw. 2,90 µg/g Kreatinin (1,5–8,7) bei den Kontrollpersonen (Gerding et al. 2021).

##### 3.1.1.2 Tierdaten

Im Nachtrag (Greim 2007) ist hierzu nur eine Studie aufgeführt. Bei je acht Kaninchen zeigte sich nach Inhalation von **Aluminiumoxid**staub (0,56 ± 0,17 mg Aluminium/m³) über einen Zeitraum von fünf Monaten (8 Stunden pro Tag, 5 Tage pro Woche) im Vergleich zu nicht exponierten Tieren ein Anstieg der Aluminiumkonzentration im Serum von ca. 0,2 mg/l auf maximal 0,45 mg/l. Neue Daten liegen nicht vor.

#### 3.1.2 Orale Aufnahme

Die Bioverfügbarkeit von Aluminiumverbindungen nach oraler Gabe wird von verschiedenen Faktoren beeinflusst. Sie wird durch einen Zusatz von Citrat, Lactat oder Ascorbat in der Ausgangslösung erhöht, wohingegen silikat- und phosphathaltige Bestandteile, Eisen- und Calciumionen oder eine Steigerung des pH-Wertes von 4 auf 7 die Aufnahme vermindern (ATSDR 2008; EFSA 2008; Greim 2007; Krewski et al. 2007).

##### 3.1.2.1 Humandaten

Die Aufnahme von Aluminium aus dem Trinkwasser beträgt 0,3 %, die aus der Nahrung wird mit 0,1 % angegeben, kann aber um den Faktor 10 variieren (EFSA 2008). Für den Menschen ist für **Aluminiumhydroxid** eine Bioverfügbarkeit nach oraler Gabe von 0,01 % angegeben (Greim 2007). Neue Daten liegen nicht vor.

##### 3.1.2.2 Tierdaten

Tierversuchsdaten zeigen eine geringe Bioverfügbarkeit nach oraler Gabe an. Die Gavage-Gabe von 2,7 ng ^26^Al als **Aluminiumhydroxid** führte bei männlichen Wistar-Ratten zu einer Bioverfügbarkeit von 0,1 %, gemessen als Radioaktivität im 5-Stunden-Urin (Greim 2007; Schönholzer et al. 1997). Bei Kaninchen ließ sich für 780 mg **Aluminiumhydroxid** und 2736 mg **Aluminiumborat** eine orale Aufnahme von 0,45 % bzw. 0,27 % beobachten (ATSDR 2008; Yokel und McNamara 1988).

Neue Studien mit oraler Gabe von schwerlöslichen **mikroskaligen Aluminiumverbindungen** bestätigen die geringe Bioverfügbarkeit und sind im Folgenden aufgeführt. Daten zu **ultrafeinen schwerlöslichen Aluminiumverbindungen** liegen nicht vor.

Je sechs weibliche Sprague-Dawley-Ratten erhielten per Schlundsonde **Aluminiumhydroxid, Aluminiumoxid** oder **elementares Aluminium**. Die Aluminiumverbindungen enthielten ^26^Al und waren in Wasser, welches 1 % Carboxymethylcellulose enthielt, suspendiert. Zusätzlichen zwölf Ratten wurde **Aluminiumcitrat**, welches 0,19 ng ^26^Al enthielt, in den Blutkreislauf injiziert. Die Kontrolltiere erhielten Wasser ohne zugesetztes Aluminium. Sieben Tage nach oraler Gabe bzw. Injektion wurde die orale Bioverfügbarkeit durch den Vergleich des verbleibenden Anteils der ^26^Al-Menge in der Karkasse nach oraler und intravenöser Gabe berechnet und diese betrug für Aluminiumhydroxid 0,03 % und für Aluminiumoxid 0,02 % (AECL Chalk River Laboratories 2010; Priest et al. 2021).

Nach sieben- oder vierzehntägiger Schlundsondengabe von **Aluminiumhydroxid** (30 mg Al/kg KG und Tag) an jeweils fünf Sprague-Dawley-Ratten pro Geschlecht wurden das Gesamtblut und Leber, Nieren, Gehirn, Rückenmark und Knochen untersucht. Die Tiere erhielten zwei Wochen vor und während der Expositionszeit aluminiumarmes Futter und Trinkwasser. In der Studie zeigten sich keine Zeichen von klinischer Toxizität. Das Körpergewicht der exponierten Tiere war nicht signifikant unterschiedlich zu dem der Kontrollgruppe. Die mittleren Aluminiumkonzentrationen in den verschiedenen Kompartimenten zeigten keine statistisch signifikanten Unterschiede zwischen den behandelten Tieren und den Kontrolltieren. Zum Teil waren die Kontrollwerte sogar höher. Nach der längeren Behandlungszeit waren die Aluminiumkonzentrationen in den meisten Geweben der behandelten sowie der Kontrolltiere niedriger, aber nicht statistisch signifikant. Die Dosis von 30 mg Al/kg KG und Tag scheint nicht ausreichend, um einen Einfluss auf den systemischen Blut- bzw. Gewebegehalt von Aluminium zu haben. Das einzige Gewebe, welches eine höhere (nicht statistisch signifikant) Aluminiumkonzentration nach 14 im Vergleich zu sieben Tagen hatte, war das Rückenmark (ToxTest 2009).

#### 3.1.3 Dermale Aufnahme

Studien zur dermalen Aufnahme von schwerlöslichen mikroskaligen Aluminiumverbindungen sind nicht verfügbar.

Die transdermale Aufnahme von **ultrafeinem Aluminiumoxid** (Partikelgröße 30–60 nm) wurde an Hautproben von zwei Frauen in einer Franzdiffusionszelle 24 Stunden nach Auftragung von 6,06 mg Aluminiumoxid/cm^2^ untersucht. Hierbei wurde sowohl intakte als auch abradierte Haut eingesetzt. Der Gehalt von Aluminium in der Rezeptorflüssigkeit (bezogen auf Applikationsfläche) betrug für die intakte Haut 35,0 ± 6,0 ng/cm^2^ (0,0011 % der applizierten Dosis), bei abradierter Haut 88,5 ± 34,2 ng/cm^2^ (0,0028 %) und bei der physiologischen Kontrolllösung 36,3 ± 47,0 ng/cm². Es zeigte sich somit kein statistisch signifikanter Unterschied zwischen intakter Haut und Kontrolle. Der Anteil der applizierten Dosis, der in der Haut gefunden wurde, betrug 0,12 % für die intakte und 0,13 % für die abradierte Haut (Mauro et al. 2019).

#### 3.1.4 Intranasale Aufnahme

Eine direkte Aluminiumaufnahme über das olfaktorische Epithel, den Bulbus olfactorius bis hin zu weiteren Strukturen im Gehirn, wird diskutiert (Yokel und McNamara 2001).

#### 3.1.5 Plazentare Aufnahme

Der plazentare Übergang von freigesetzten Aluminiumionen vom Muttertier zum Fetus ist für Ratten und Mäuse belegt (Hartwig und MAK Commission 2025). In den Studien zur pränatalen Entwicklungstoxizität mit schwerlöslichen Aluminiumverbindungen wurde **Aluminiumhydroxid** vom 6. bis zum 15.  Gestationstag per Schlundsonde verabreicht (Colomina et al. 1992, 1994; Domingo et al. 1989; Gomez et al. 1990, 1991), wobei keine entwicklungstoxischen Effekte festgestellt wurden. Im gesamten Fetus von Mäusen betrugen die Aluminiumkonzentrationen bei der Dosis von 103 mg Al/kg KG und Tag: 0,88 ± 0,75 µg Al/g Feuchtgewicht; Kontrollgruppe: 0,66 ± 0,59 µg Al/g Feuchtgewicht (Colomina et al. 1994), bei 57,5 mg Al/kg KG und Tag: 0,18 ± 0,27 µg Al/g Feuchtgewicht; Kontrollgruppe: unter der Nachweisgrenze (0,05 µg Al/g Feuchtgewicht; Colomina et al. 1992). Die Werte stammen aus derselben Arbeitsgruppe, der eingesetzte Stamm, die Testsubstanz Aluminiumhydroxid, Futter, Trinkwasser sowie die Analytik waren gleich. Unklar bleibt, warum die Kontrollgruppen unterschiedliche Aluminiumkonzentrationen im Gesamtfetus aufweisen. Weder im Trinkwasser noch im Futter wurden die Aluminiumkonzentrationen gemessen (Colomina et al. 1992, 1994). In der Studie an Ratten waren bis zur höchsten Dosis von 266 mg Al/kg KG und Tag keine erhöhten Aluminiumkonzentrationen in Leber, Gehirn und Knochen der Muttertiere festzustellen. Im gesamten Fetus lag die Aluminiumkonzentration in allen Dosisgruppen unterhalb der Nachweisgrenze (0,05 µg/g; Gomez et al. 1990).

Während der Schwangerschaft kommt es aufgrund des intrauterinen Wachstums zu einer Mobilisierung von Metallionen aus den maternalen Knochen zum Fetus hin, was für Blei gezeigt ist (ATSDR 2020). Aus einer vergleichenden Untersuchung von trächtigen (Exposition von GD 1–20, Untersuchung GD 20) und nicht-trächtigen Ratten (Behandlung 20 Tage lang) mit **Aluminiumhydroxid** (0, 200, 400 mg/kg KG und Tag, Gavage; 0, 70, 140 mg Al/kg KG und Tag) geht hervor, dass es zu einer Umverteilung von Aluminium während der Trächtigkeit kommt. So waren die Aluminiumkonzentrationen bei trächtigen Ratten bei 140 mg Al/kg KG und Tag am höchsten in den Nieren und am niedrigsten im Gehirn, bei nicht-trächtigen Tieren am höchsten im Gehirn und am niedrigsten in der Leber. Bei den Kontrollgruppen war ebenfalls die höchste Aluminiumkonzentration im Gehirn. Die Aluminiumkonzentration in den Feten wurde nicht bestimmt (Bellés et al. 2001). Das ist als Hinweis für eine mögliche Umverteilung von Aluminium während der Trächtigkeit in den Fetus zu werten. Diese Umverteilung findet unabhängig von einer externen Zufuhr statt.

#### 3.1.6 Verteilung 

Daten zur Verteilung von Aluminium im Körper sind im Nachtrag von Greim (2007) aufgeführt. Im Folgenden werden neu verfügbare Daten dargestellt.

Fischer-344-Ratten wurden 4–6 Stunden lang einmalig gegen 0; 9,7; 18,1 oder 20,9 mg/m³ eines Aerosols aus **ultrafeinem Aluminiumoxid** (20,3 ± 4,3 nm) exponiert und Lunge, Lymphknoten, Leber, Nieren und Milz nach 24 Stunden oder sieben Tagen mikroskopisch untersucht. Bei vierstündiger Exposition gegen die niedrigste Konzentration zeigten sich nach sieben Tagen im Alveolargewebe der Lunge Cluster von Aluminiumoxidpartikeln im Gefäßlumen, die von roten Blutzellen umgeben waren. Zudem traten Cluster in Endothelzellen der Blutgefäße der Nieren, die an Glomeruli angrenzten, auf. Ratten, die sechs Stunden die höchste Aluminiumoxid-Konzentration inhalierten, wiesen nach sieben Tagen Aluminiumoxid-Cluster in den Lymphknoten der Lungen, welche von weißen Blutzellen umgeben waren, in Epithelzellen des Gallengangs der Leber und in der weißen Pulpa der Milz auf (Guttenberg et al. 2016). Die Aluminiumkonzentrationen wurden nicht quantifiziert.

In einer 28-Tage-Studie wurden männliche Wistar-Ratten gegen **Aluminiumoxyhydroxid** (10 nm oder 40 nm Primärpartikel, MMAD: 1,7 μm bzw. 0,6 μm) als Aerosol in Konzentrationen von 0; 0,4; 3 oder 28 mg/m^3 ^(0; 0,16/0,18; 1,18/1,32; 11,03/12,29 mg Al/m^3^) nur über die Nase, sechs Stunden pro Tag, an fünf Tagen pro Wochen exponiert (siehe Abschnitt 5.2.1). Hierbei war die Verteilung in Lungen-assoziierte Lymphknoten von der Akkumulation in der Lunge abhängig, die bei 40-nm-Partikeln im Vergleich zu den 10-nm-Partikeln bei gleicher Expositionskonzentration aufgrund des geringeren MMAD höher war. Bei beiden niedrigen Konzentrationen konnte kein Aluminium in den Lymphknoten nachgewiesen werden. Es ließ sich keine erhöhte Aluminiumbelastung in Gehirn, Leber und Nieren feststellen (Pauluhn 2009 b).

Männliche Sprague-Dawley-Ratten erhielten an drei aufeinander folgenden Tagen per Schlundsonde **ultrafeines, elementares Aluminium** oder **ultrafeines Aluminiumoxid** (k. A. zur Modifikation) in Dosen von 6; 12,5 oder 25 mg/kg KG und Tag (entspricht bei Al_2_O_3_: 3,2; 6,6 und 13,2 mg Al/kg KG und Tag) oder 25 mg **Aluminiumchloridhexahydrat**/kg KG und Tag (2,8 mg Al/kg KG und Tag). In Leber, Nieren, Milz, Dünndarm und Dickdarm wurde drei Stunden nach der letzten Gabe Aluminium unabhängig von der Art der eingesetzten Verbindung nachgewiesen. Die Autoren sehen darin einen Unterschied zu der inhalativen Studie von Pauluhn (2009 b), bei der außer in der Lunge keine signifikante Aluminiumbelastung in Leber, Nieren und Gehirn beobachtet wurde. In der Leber zeigte sich ein geringerer Aluminiumgehalt nach Gabe von elementarem Aluminium im Vergleich zum Aluminiumchlorid. Im Gegensatz dazu war der Aluminiumgehalt nach Gabe von Aluminiumoxid im Vergleich zum Aluminiumchlorid in der Leber höher. Mit zunehmender Dosis zeigte sich eine Abnahme, was die Autoren auf eine stärkere Agglomeration und dadurch geringerer intestinaler Aufnahme bei höheren Dosen zurückführten. Im Blut ließ sich für die ultrafeinen Aluminiumverbindungen im Vergleich zu Aluminiumchlorid deutlich mehr Aluminium nachweisen, wobei jeweils die geringste Dosierung die stärkste Zunahme zeigte. Die Konzentration von Aluminiumoxid im Blut war hierbei im Vergleich zum elementaren Aluminium und zum Aluminiumchlorid 10-fach bzw. 100-fach höher. Die Autoren vermuten, dass die ultrafeinen Aluminiumverbindungen, insbesondere Aluminiumoxid, im Blut mit hochmolekularen Plasmaproteinen interagieren, was deren Zugang zu Geweben limitiert und somit letztlich die Halbwertszeit im Blut erhöht (Krause et al. 2020).

An Wistar-Ratten wurde die Verteilung in Blut, Leber, Milz, Herz, Nieren, Gehirn, Urin und Faeces 14 Tage nach einmaliger oraler Gabe von **ultrafeinen Aluminiumoxidpartikeln** (30 nm, 40 nm, k. A. zur Modifikation) sowie **mikroskaligem Aluminiumoxid** in Dosierungen von 0, 500, 1000 oder 2000 mg/kg KG (0, 265, 529, 1059 mg Al/kg KG) untersucht. Für die ultrafeinen Partikel ist ab 1000 mg Aluminiumoxid/kg KG (Nieren, Urin schon ab 500 mg Aluminiumoxid/kg KG) eine statistisch signifikante, dosisabhängige Zunahme der Konzentration in den Organen zu beobachten. Zudem wurden eine Aufnahme und Verteilung in das Blut gezeigt. Für mikroskaliges Aluminiumoxid ließ sich eine statistisch signifikante Zunahme nur in den Faeces beobachten, in den anderen Geweben waren die Spiegel allerdings auch deutlich und dosisabhängig erhöht (Balasubramanyam et al. 2009 a, b). In beiden Publikationen ist eine Messung der Verteilung abgebildet. Obwohl hierbei die gleichen Messwerte berichtet werden, unterscheiden sich die Angaben dahingehend, ob sich die Werte auf Aluminiumoxid oder Aluminium beziehen. Wie der ECHA-Datenbank zu entnehmen ist, ergab die Rücksprache mit den Autoren, dass Aluminiumoxid bestimmt wurde (ECHA 2023 c). Im Hinblick auf die geringen Standardabweichungen und niedrigen Werte der Kontrollen ist es nicht plausibel, wieso die Werte bei 500 mg/kg KG in Blut, Leber, Milz, Herz und Gehirn keine statistische Signifikanz aufweisen. Weitere Kritikpunkte zu dieser Studie sind, dass die ultrafeinen Partikel nicht ausreichend charakterisiert waren und einen hohen Gehalt an Verunreinigungen aufwiesen (siehe Abschnitt 5.6.2). Da die Untersuchung nur zu einem Zeitpunkt, 14 Tage nach der Dosierung, durchgeführt wurde, stellt sie eine Momentaufnahme der Belastung der Organe dar.

Jeweils zwölf ICR-Mäuse erhielten 13 Wochen lang **ultrafeines Aluminiumoxid** (γ-Modifikation, < 50 nm) in Dosierungen von 0; 1,5; 3 oder 6 mg/kg KG und Tag mit dem Trinkwasser (siehe auch Abschnitt 5.2.2). Am Studienende war im Vergleich zu der Kontrollgruppe die höchste Akkumulation von Aluminium in der Leber > Nieren > Herz > Lunge > Thymus. Der Gehalt im Gehirn war geringer als in der Kontrolle (Park et al. 2015). Von der selben Arbeitsgruppe wurde eine weitere Untersuchung mit ICR-Mäusen (sechs Tiere pro Gruppe) durchgeführt, welche 28 Tage lang, an sechs Tagen pro Woche ultrafeine Aluminiumoxidpartikel in Dosierungen von 1 oder 6 mg Aluminium/kg KG per Schlundsonde erhielten. Es ließen sich statistisch signifikant erhöhte Aluminiumkonzentrationen für beide Dosierungen im Herz, in den Nieren und im Blut feststellen sowie nur für die höhere Konzentrationsgruppe in Thymus und Milz (Park et al. 2017).

Swiss-Albino-Mäuse erhielten fünf Tage lang** ultrafeines Aluminiumoxid** (vermutlich γ-Modifikation laut Herstellerkatalog) in den Dosierungen von 0, 15, 30 oder 60 mg/kg KG und Tag (0, 8, 16, 32 mg Al/kg KG und Tag). Hierbei zeigte sich im Vergleich zur Kontrolle eine Zunahme der Aluminiumkonzentration im Gehirn (ab 16 mg Al/kg KG und Tag), in der Leber (nur bei 16 mg Al/kg KG und Tag), der Milz (ab 8 mg Al/kg KG und Tag), den Nieren (bei 32 mg Al/kg KG und Tag) und den Hoden (ab 16 mg Al/kg KG; De et al. 2020, siehe auch Abschnitt 5.2.2).

Zusammenfassend spiegeln die Studien mit wiederholter oraler Gabe von schwerlöslichen Aluminiumverbindungen nicht die Verteilung nach Inhalation wider, bei der außer in der Lunge keine signifikante Aluminiumbelastung in Leber, Nieren und Gehirn beobachtet wurde und sie sind daher nicht für eine Bewertung der Toxizität am Arbeitsplatz geeignet.

#### 3.1.7 Ausscheidung

Daten zur Ausscheidung von Aluminium sind in Greim (2007) aufgeführt. Im Folgenden sind neue Studien zur Ausscheidung schwerlöslicher Aluminiumverbindungen aufgeführt.

Zwölf Probanden, welche vor Studienbeginn nicht arbeitsbedingt geschweißt hatten, führten einmalig sechs Stunden lang Schweißprozesse durch, bei denen sie gegen aluminiumhaltigen Schweißrauch exponiert waren. Der Schweißrauch (stationär gemessen) hatte eine Gesamt-Staubkonzentration von 2,5 mg/m^3^. Dabei betrug die Partikelgröße 40–340 nm. Der prozentuale Anteil von Aluminium im Schweißrauch betrug 51,43 ± 0,22 %, in der Luft wurden 1,29 mg Al/m^3^ gemessen. Urin und Plasmaproben wurden unmittelbar vor und nach sowie einen und sieben Tage nach der Exposition genommen und der Aluminiumgehalt mittels Atomabsorptionsspektrometrie untersucht. Die Konzentrationen von Aluminium im Urin zeigten statistisch signifikante Veränderungen nach der Exposition im Vergleich zu den Proben vor der Exposition (siehe Tabelle 2). Im Plasma stieg der Aluminiumgehalt nach sechs Stunden an, 24 und 168 Stunden nach der Exposition war der Gehalt unterhalb des Wertes der Messung vor der Exposition. Ein statistisch signifikanter Unterschied zwischen vor und nach der Exposition wurde nicht festgestellt. Die Autoren erwähnen, dass die Plasmakonzentrationen nach der Exposition unterhalb der Referenzwerte der Allgemeinbevölkerung lagen (Bertram et al. 2015).

**Tab. 2:** Aluminiumkonzentration in Plasma und Urin von exponierten Probanden (Bertram et al. 2015)

**Stunden nach Expositionsbeginn**	**Aluminium im Plasma** **[µg/l]**	**Aluminium im Urin** **[µg/l]**	**Aluminium im Urin ** **[µg/g Kreatinin]**
0	3,2	13,5	10,6
6	4,7^a)^	23,5*	15,0
24	3,1	18,0*	19,1**
168	2,7	16,9*	14,0

^a)^ Der Datensatz beinhaltet einen Ausreißer mit 14,7 µg/l Plasma. Der Aluminiumgehalt im Plasma ohne Ausreißer liegt bei 3,8 µg/l.

* p nach 6 h: 0,001; 24 h: 0,023; 168 h: 0,024;

** p nach 24 h: 0,007

In einer 28-Tage-Studie wurden männliche Wistar-Ratten gegen **Aluminiumoxyhydroxid** (10 nm oder 40 nm Primärpartikel, MMAD: 1,7 μm bzw. 0,6 μm) als Aerosol in Konzentrationen von 0; 0,4; 3 oder 28 mg/m^3 ^(0; 0,16/0,18; 1,18/1,32; 11,03/12,29 mg Al/m^3^) nur über die Nase, sechs Stunden pro Tag, an fünf Tagen pro Wochen exponiert (siehe Abschnitt 5.2.1). Für die Gruppe der gegen 40-nm-Partikel exponierten Tiere wurde der Aluminiumgehalt im Urin an vier Tagen bestimmt (siehe Tabelle 3). Wenn auch die 28-Tage-Studie zu kurz war, um eine Dosisabhängigkeit der Aluminiumkonzentration im Urin nachzuweisen, ist bei der höchsten Konzentration ab dem 11. Tag eine Erhöhung und am 25. Expositionstag ein deutlicher Anstieg von Aluminium im Urin zu beobachten (Bayer Schering Pharma AG 2008; Pauluhn 2009 b).

**Tab. 3:** Aluminiumgehalt (µg Al/mmol Kreatinin, Mittelwert (SD), n = 6) im Urin von Wistar-Ratten nach bis zu 25-tägiger Exposition gegen Aluminiumoxyhydroxid-Aerosol (40 nm große Primärpartikel) (Bayer Schering Pharma AG 2008; Pauluhn 2009 b)

**Exposition [mg Al/m^3^]**	**Expositionstage**			
	**4**	**11**	**18**	**25**
0	5,94 (3,74)	3,14 (2,50)	6,70 (5,30)	5,21 (4,26)
0,18	3,83 (1,56)	3,13 (2,29)	3,13 (1,47)	2,31 (1,39)
1,32	6,24 (2,48)	5,28 (2,81)	4,01 (1,83)	4,85 (2,76)
12,29	3,97 (1,44)	9,12 (9,57)	9,48 (5,83)	19,05 (20,22)

### 3.2 Metabolismus 

Aluminium wird nicht metabolisiert. Schwerlösliche Aluminiumverbindungen lösen sich unter sauren Bedingungen (siehe Tabelle 1) wie sie in den Lysosomen der Lunge oder im Magen vorliegen.

## 4 Erfahrungen beim Menschen

### 4.1 Einmalige Exposition 

In Übersichtsarbeiten (ATSDR 2008; WHO 2003; Willhite et al. 2014) sind keine Humandaten nach einmaliger Exposition gegen Aluminium oder schwerlösliche Aluminiumverbindungen aufgeführt. Eine Studie mit zweistündiger Exposition und Untersuchung von Entzündungsparametern im Sputum (Sikkeland et al. 2016) ist in Abschnitt 4.4.2 dargestellt. Insgesamt stehen Effekte nach einmaliger Exposition nicht im Vordergrund dieser Bewertung.

### 4.2 Wiederholte Exposition 

#### 4.2.1 Wirkungen auf das respiratorische System

Studien zu respiratorischen Effekten nach wiederholter Exposition gegen Aluminium am Arbeitsplatz sind in Greim (2007) ausführlich beschrieben und die für die Diskussion zur Grenzwertableitung relevanten Studien werden im Folgenden zusammengefasst dargestellt. Neue Studien wurden ergänzt (siehe Tabelle 4).

**Tab. 4:** Befunde bei Beschäftigten nach chronischer, arbeitsplatzbedingter Aluminiumexposition

**Kollektiv, Land**	**Expositionshöhe**	**Befunde**	**Untersuchung**	**Literatur**
**Al-Schweißer**, 25 Beschäftigte, 25 Kontrollen, Exposition [a]: Beschäftigte: 12 (> 2,5), Kontrollen: 13 (< 2,5),**Schweden**	**Al-Konzentration** (Median (Bereich)) (n = 14): 1,4 (0,2–6,1) mg/m^3^, **Gesamtstaub** (n = 19): 2,8 (0,7–19) mg/m^3^, personenbezogen, **Urin **(n = 19): Freitag: 0,29 (0,08–1,1) mmol/mol Kreatinin, Montag: 0,16 (0,07–1,16) mmol/mol Kreatinin	**Respiratorische Symptome:** Beschäftigte (> 2,5 a): nicht spezifische bronchiale Hyperreagibilität (5/12)*, Beschäftigte (< 2,5 a): Nasenverstopfung (4/13)*, Pharyngitis (5/13)*, nicht spezifische bronchiale Hyperreagibilität (4/13)*, bronchiale Hyperreagibilität (Langzeitschweißer) ↑*, **Spirometrie:** keine Effekte, **Blut: **IgA ↓***	Symptome, Spirometrie: VC, FEV_1_, MEF_25_, Blut: IgA, IgE, IgG, IgM	Greim 2007; Nielsen et al. 1993
**Werftarbeiter**, 12 Metallarbeiter, 38 Schweißer, 50 Kontrollen, Exposition [a]: Beschäftigte: 11,8 (Mittelwert) ± 3,71, **Italien**	**Al-Konzentration**: 6,2–20,2 mg/m^3^, personenbezogen, **Blut** (Mittelwert ± SD): Beschäftigte: 32,64 ± 8,69 µg/l, Kontrollen: 6,43 ± 1,00 µg/l	**Spirometrie:** VC ↓**, FVC ↓**, FEV_1_ ↓**, FEV_1_/VC ↓**, FEF_25-75_ ↓**, **Röntgenaufnahme: **keine Effekte	Spirometrie: VC, FVC, FEV_1_, FEV_1_/VC, FEF_25-75_, Röntgenaufnahmen im Brustbereich	Abbate et al. 2003
**Al-Gießerei**, Exposition [a]: Schmelzer (n = 50): 14,8 ± 8,0, Schlosser (n = 5): 28,4 ± 2,0, Säge-/Hilfsarbeiter (n = 11): 15,8 ± 7,8, Kontrollen (n = 42): 20,9 ± 10,9 **Polen**	**Al_2_O_3_-Konzentration**: Schmelzer: 0,32 ± 0,18 mg/m^3^, Schlosser: 0,41 ± 0,18 mg/m^3^, Säge-/Hilfsarbeiter: 0,61 ± 0,63 mg/m^3^, personenbezogen, **Al-Konzentration**: Urin: Schmelzer: 43,7 ± 23,7 µg/l, Schlosser: 35,2 ± 12,4 µg/l, Säge-/Hilfsarbeiter: 21,7 ± 7,4 µg/l, Kontrollen: 10,2 ± 7,7 µg/l, Serum: Schmelzer: 1,3 ± 1,1 µg/l, Schlosser: 1,16 ± 0,6 µg/l, Säge-/Hilfsarbeiter: 1,16 ± 1,2 µg/l, Kontrollen: 1,2 ± 1,1 µg/l	**Spirometrie:** FEV_1_ ↓, FEV_1_/FVC ↓, FEV_50_ ↓ (Schlosser stärker als Schmelzer betroffen, k. A. zur Signifikanz), Schmelzer: Verschlechterung, Lungenfunktion korreliert mit Al_2_O_3_-Konzentration in der Luft, SOD ↑*, MPO ↑*, CC16 ↓*, **Lineare Regressionsanalyse:** (Ausschluss Rauchen, Alter) Al-Urin-Konzentration stat. sig. negative Korrelation mit FVC, FEV_1_, Al-Serum-Konzentration stat. sig. negative Korrelation mit CC16 (nur bei Rauchern)	Funktion des bronchiolären Epithels (CC16 im Serum), Spirometrie: FVC, FEV_1_, FEV1/FVC, FEV_50_, Blutserum: Fe, MPO, ECP, IgE, GST, SOD	Hałatek et al. 2006
**Al-Schweißer**, Automobilindustrie, Beschäftigte: Studienbeginn (n = 98), Kontrolle (n = 50) Exposition [a]: 1999: 4,7 ± 1,6 2003: 9 ± 2, **Deutschland**	**Al-haltiger Schweißrauch**^a)^, alveolengängige Fraktion, **Rauch-Konzentration Luft **(Median (Bereich)): **1999** (n = 50): 0,47 (0,1–6,17) mg/m^3^ (Buchta et al. 2003; Letzel et al. 2006), **2001** (n = 26): 0,67 (0,2–1,5) mg/m^3^ (Buchta et al. 2003; Letzel et al. 2006), 0,4 (0,2–1,4) mg/m^3^ (Kiesswetter et al. 2009), **2003** (n = 26): 0,7 (0,2–1,5) mg/m^3^ (Kiesswetter et al. 2009), 0,55 (0,15–0,96) mg/m^3^ (Letzel et al. 2006), personenbezogene Messungen, **keine** Bestimmung der Al-Konzentration, **Al-Konzentration** (Median): Urin: **1999**: Vorschicht: 71,8 µg/l, 38,4 µg/g Krea, Nachschicht: 47,6 µg/l, 37,9 µg/g Krea, **2001**: Vorschicht: 58,3 µg/l, 35,5 µg/g Krea, Nachschicht: 39,8 µg/l, 33,6 µg/g Krea, **2003**: Vorschicht: 21,7 µg/l, 12,6 µg/g Krea, Nachschicht: 16,1 µg/l, 15,4 µg/g Krea, Plasma: **1999**: Vorschicht: 10,3 µg/l, Nachschicht: 8,3 µg/l, **2001**: Vorschicht: 4,3 µg/l, Nachschicht: 4,1 µg/l, **2003**: Vorschicht u. Nachschicht: 4,3 µg/l	**Respiratorische Symptome:** Atembeschwerden, Husten ohne/mit Auswurf im Längsschnittverlauf ↓^b)^ **Spirometrie:** keine Hinweise auf restriktive Atemwegserkrankung, **HRCT:** emphysematöse, bullöse und bronchitische Lungenveränderungen, insb. Raucher u. Ex-Raucher (96 %) betroffen → nicht alleinig auf Al-Schweißen zurückführbar	Respiratorische Symptome, Spirometrie: VC_max_, FVC, FEV_1_, FEV_1/_VC_max, _MEF_50_, MEF_25_, HRCT	Buchta et al. 2003; Greim 2007; Kiesswetter et al. 2009; Letzel et al. 2006
**Al-Verarbeitung** (Wertstoff- oder Altmaterialbetrieb), **Kollektiv 2007** 30 Al-Beschäftigte, 60 Kontrollen, Exposition [Mo]: 78,1 ± 51,3, **Kollektiv 2012** 36 Al-Beschäftigte, 36 Kontrollen, Exposition [Mo]: 111 ± 77,5, **Frankreich**	**Al-Konzentration** (Mittelwert (Bereich) od. ± SD): personenbezogen **Kollektiv 2007** (n = 8) 2,23 (0,12–10,86) mg/m^3^, Urin (n = 30): 11,02 ± 8,23 µg/l (Vorschicht), 11,59 ± 10,09 µg/l (Nachschicht), Kontrolle: 4,37 ± 3,67 µg/l, Plasma: 3,78 ± 4,1 µg/l, Kontrolle: 3,31 ± 3,38 µg/l, **Kollektiv 2012** (k. w. A.): 1,61 (0,13–3,13) mg/m^3^, Urin (n = 25): 20,84 ± 27,85 µg/l (Vorschicht), 20,12 ± 31,61 µg/l (Nachschicht), Kontrolle: 13,8±13,33 µg/l	**Respiratorische Symptome:** Kollektiv 2007: keine Effekte, Kollektiv 2012: chronischer Husten ↑*, 9/36 (25 %) im Vgl. zur Kontrollgruppe	Spirometrie: FEV_1_, FEV_1_/FVC, Röntgenaufnahmen im Brustbereich**,** abgefragte respiratorische Symptome: respiratorische Insuffizienz, chronischer Husten, Knisterrasseln (Lunge), Atemgeräusche	Deschamps et al. 2009, 2018

* p < 0,05;

** p < 0,01;

*** p < 0,001

a: Jahre; ECP: eosinophil cationic protein; FVC: forcierte exspiratorische Vitalkapazität; FEV_1_: forcierte exspiratorische Sekundenkapazität; GST: Glutathion-S-Transferase; HRCT: High-Resolution-Computertomographie; Ig: Immunglobulin; Krea: Kreatinin; MEF_25_, MEF_50_: maximaler exspiratorischer Fluss bei 25 bzw. 50 % Vitalkapazität; Mo: Monate; MPO: Myeloperoxidase; SD: Standardabweichung; SOD: Superoxid-Dismutase; stat. sig.: statistisch signifikant; VC (auch VC_max_): Vitalkapazität; Vgl.: Vergleich; ↓: vermindert; ↓↓: stark vermindert; ↑: erhöht

^a)^ Es ist nicht untersucht worden, wie hoch der Al-Anteil im Schweißrauch ist. Wird davon ausgegangen, dass Aluminium unverändert vorliegt, ist von einem Aluminiumanteil von mehr als 90 % auszugehen, liegt Aluminium als Al_2_O_3_ vor, ist der Al-Anteil im Schweißrauch ca. 50 %.

^b)^ Im Jahr 2001, 2003 führten nicht alle exponierten Probanden regelmäßig Al-Schweißarbeiten durch.

In der Untersuchung an Beschäftigten einer Aluminiumgießerei sind die geringsten Expositionskonzentrationen berichtet. Es handelt sich um einmalige Nach-Schicht-Expositionsmessungen mit parallelen einmaligen Bestimmungen gesundheitlicher Parameter, die es nicht erlauben, einen direkten Zusammenhang zwischen Exposition und Effekten herzustellen, da unklar ist, inwiefern frühere Expositionen für die Befunde relevant sind. Die Spirometrien wurden nach der Schicht durchgeführt, wodurch mögliche Across-Shift-Verschlechterungen von dauerhaften Lungenfunktionseinschränkungen nicht nachweisbar sind. Die Messergebnisse für das Keulenzellenproteins CC16 im Serum und der Lungenfunktionstests können nur für die Schmelzer bewertet werden, da hier Stichprobenumfang, Alter und Raucherstatus ähnlich denen der Kontrollen sind. Die Lungenfunktionsparameter der Schmelzer zeigen im Vergleich zur Kontrollgruppe keine relevanten Veränderungen. Angaben zur statistischen Signifikanz liegen nicht vor. Die angegebene Dosis-Wirkungs-Beziehung bei den Schmelzern (siehe Abbildung 3 in Hałatek et al. 2006) ist nicht bewertbar, da sie auf einer Klassifikation von Obstruktionsgraden beruht, welche nicht näher definiert sind. Die CC16-Protein-Konzentration im Serum der Schmelzer war statistisch signifikant geringer (Hałatek et al. 2006; Hartwig und MAK Commission 2019). Bei akuter Exposition mit Lungenreizstoffen wie Chlor oder Ozon steigt der Serum-CC16-Spiegel vorübergehend an, während chronische Exposition wie Rauchen oder berufliche Exposition gegen Siliciumdioxid mit einem verminderten CC16-Serumwert verbunden ist (Rava et al. 2013). Allerdings ist die Konzentration von CC16 von anderen Faktoren wie Rauchen, Umgebungstemperatur, Tageszeit, Infektionen oder sonstiger inhalativer Exposition abhängig, wobei diese Faktoren in der Studie nicht oder nur unzureichend beschrieben wurden. Abschließend ist die Studie aufgrund der dargestellten Unsicherheiten nicht für eine Grenzwertableitung geeignet.

Von der gleichen Arbeitsgruppe sind weitere Veröffentlichungen mit dem gleichen Kollektiv verfügbar. Bei der Studie von Hałatek et al. (2005) handelt es sich um eine Teilanalyse, da nur die Ergebnisse für das Schmelzerkollektiv beschrieben wurden. In Hałatek et al. (2008) wurde das Kollektiv anhand der Ergebnisse von neurologischen Untersuchungen (visuell evozierte Potenziale (VEP), Elektroenzephalographie (EEG)) in weitere Gruppen aufgeteilt. In beiden Veröffentlichungen (Hałatek et al. 2005, 2008) werden keine neuen Daten zum Endpunkt Lungentoxizität geliefert, weshalb sie in der Übersicht (Tabelle 4) nicht aufgenommen wurden.

Es liegt eine Längsschnittstudie mit drei Untersuchungen in den Jahren 1999, 2001 und 2003 bei Aluminiumschweißern im Automobilbau mit einer Exposition gegen aluminiumhaltigen Schweißrauch von 0,4 bis 0,7 mg/m^3^ vor. Im Längsschnittverlauf ist bei den Aluminiumschweißern insbesondere für das Jahr 2003 eine Abnahme der Atemwegsbeschwerden zu verzeichnen. Die Autoren weisen darauf hin, dass nicht mehr alle Aluminiumschweißer aus dem Jahr 1999 im Jahr 2003 Schweißarbeiten ausgeführt haben, was ggf. die Reduktion der angegebenen Atemwegsbeschwerden erklären könnte. Im untersuchten Kollektiv zeigen die Aluminiumschweißer im Vergleich zu den Kontrollen bei den Lungenfunktionsuntersuchungen schlechtere Werte bei den Parametern „maximaler exspiratorischer Fluss“ (MEF)_25_ und MEF_50_, die auf eine Obstruktion im Bereich der kleinen Atemwege hindeuten. Keine Hinweise ergeben sich für eine restriktive Lungenerkrankung, wie sie u. a. häufig bei Personen mit einer Aluminiumstaublunge im fortgeschrittenen Stadium beobachtet werden. In einer hochauflösenden Computertomographie (HRCT)-Untersuchung fallen bei den Aluminiumschweißern emphysematöse, bronchitische und bullöse Lungenveränderungen auf, die vor allem auf das Zigarettenrauchen zurückgeführt werden. Zusammenfassend ist festzustellen, dass die bei dem untersuchten Kollektiv gefundenen Lungenveränderungen durch Zigarettenrauch, Aluminiumschweißen oder Ozonexposition verursacht sein könnten. Eine abgrenzende Wertung der Einzelfaktoren ist jedoch nicht möglich (Buchta et al. 2003; Kiesswetter et al. 2009; Letzel et al. 2006).

Die Studien von Nielsen et al. (1993) und Deschamps et al. (2018) zeigen respiratorische Effekte in einem ähnlichen Expositionsbereich von 1,4 bzw. 1,61 mg Aluminium/m^3^. Letztere Untersuchung ist aufgrund mehrerer Einschränkungen (siehe Abschnitt 4.2.2.1) nur hinweisgebend und kann nicht zur Grenzwertableitung herangezogen werden.

Studien bei denen von Mischexpositionen auszugehen ist, beispielsweise bei Beschäftigten in Bauxit-Minen und Aluminiumraffinerien, werden nicht zur Bewertung herangezogen (u. a.: Cavallari et al. 2008; Del Monaco et al. 2020; Edling et al. 1987; Godderis et al. 2005; Hartmann et al. 2014; Jederlinic et al. 1990; Musk et al. 2000; Neophytou et al. 2019; Sjögren und Ulfvarson 1985).

**Fazit respiratorische Effekte: **Von den aufgeführten Studien, die Effekte auf das respiratorische System zeigen, ist keine ausreichend aussagekräftig, um daraus allein einen Grenzwert für Lungenveränderungen abzuleiten (siehe Tabelle 5). Die Studie mit den niedrigsten gemessenen Konzentrationen von aluminiumhaltigem Schweißrauch im Bereich von 0,47–0,76 mg/m^3^ zeigt Lungeneffekte, die allerdings nicht allein auf Aluminium, sondern auch auf eine Koexposition mit Ozon oder Rauchen zurückzuführen sein könnte. Eine NOAEC kann folglich nicht abgeleitet werden.

**Tab. 5:** Übersicht der Arbeitsplatz-Studien

**Konzentration Luft [mg/m^3^]**	**Lungenfunktion**	**obere Atemwege**	**Blutparameter/HRCT **	**Literatur**
Al_2_O_3_: 0,32 ± 0,18	Spirometrie: kein Effekt	n. u.	Blut: stat. sig. ↑ SOD, MPO; CC16 ↓/ n. u.	Hałatek et al. 2006
Schweißrauch: 0,47–0,67	Spirometrie: kein Effekt	Atembeschwerden, Husten und Husten mit Auswurf im Längsschnittverlauf ↓	Blut: n. u./emphysematöse, bullöse u. bronchitische Lungenveränderungen	Buchta et al. 2003; Kiesswetter et al. 2009; Letzel et al. 2006
	→ Effekte nicht allein auf Al-Schweißen zurückzuführen			
Al: Kollektiv 2007: 2,23 Kollektiv 2012: 1,61	Spirometrie: kein Effekt	stat. sig. ↑ chronischer Husten	n. u.	Deschamps et al. 2009, 2018
Al: 1,4	Spirometrie: kein Effekt	stat. sig. ↑ Pharyngitis, Nasenverstopfung, nicht spezifische bronchiale Hyperreagibilität	Blut: stat. sig. IgA ↓/n. u.	Nielsen et al. 1993
Al: 6,2–20,2	stat. sig. ↓ Lungenfunktionsparameter	n. u.	n. u.	Abbate et al. 2003

CC16: Keulenzelprotein; IgA: Immunglobolin A; MPO: Myeloperoxidase; n. u.: nicht untersucht; SOD: Superoxid-Dismutase; stat. sig.: statistisch signifikant

#### 4.2.2 Wirkungen auf das zentrale Nervensystem 

Im Jahr 2017 wurde ein BAT-Wert von 50 µg Aluminium/g Kreatinin abgeleitet (Klotz et al. 2018) zum Schutz vor präklinischen neurotoxischen Effekten. In der oben erwähnten Längsschnittuntersuchung mit drei Querschnittstudien in den Jahren 1999, 2001 und 2003 wurden zwei Teilkollektive untersucht, wobei für die gegen Aluminium exponierten Schweißer des Schienen- und Spezialfahrzeugbaus ein LOAEL von ca. 100 µg Aluminium/g Kreatinin (ca. 120 µg Aluminium/l Urin, 13 µg Aluminium/l Plasma) aufgrund schlechterer Leistungen in wiederholt durchgeführten neuropsychologischen Tests festgelegt worden ist. Die Schweißer des Schienen- und Spezialfahrzeugbaus waren zu den drei jeweiligen Untersuchungszeitpunkten gegen Schweißrauch in Konzentrationen (Median) von 5,6 mg/m^3^ (Bereich: 0–31,5); 4,5 mg/m^3^ (Bereich: 1,9–29,7) bzw. 6,8 mg/m^3^ (Bereich: 1,9–29,7) exponiert. Anhand der Daten aus dem zweiten Teilkollektiv, Beschäftigte des Automobilbaus, ließ sich für die Neurotoxizität ein NOAEL von 38 µg Aluminium/g Kreatinin (ca. 45 µg Aluminium/l Urin, 5 µg Aluminium/l Plasma) festlegen. Die Arbeiter waren zu den drei jeweiligen Untersuchungszeitpunkten alveolengängigen Staubkonzentrationen (Median) von 0,47 mg/m^3^ (Bereich: 0,1–6,17); 0,67 mg/m^3^ (Bereich: 0,2–1,5) bzw. 0,7 mg/m^3^ (Bereich: 0,2–1,5) ausgesetzt (Buchta et al. 2005; Kiesswetter et al. 2007; Letzel et al. 2006). Die Konzentration von Aluminium im Urin korrelierte nicht mit der Luftkonzentration (Kiesswetter et al. 2007). Es ist nicht untersucht worden, wie hoch der Al-Anteil im Schweißrauch ist. Falls Aluminium unverändert vorliegt, ist von einem Aluminiumanteil von mehr als 90 % auszugehen, wird davon ausgegangen, dass Aluminium als Al_2_O_3_ vorliegt, ist der Al-Anteil im Schweißrauch ca. 50 %.

Im Folgenden sind neue Arbeitsplatzstudien ab dem Jahr 2017 aufgeführt, sowie zusätzliche relevante Studien zur neurotoxischen Wirkung. Metaanalysen, Fallberichte sowie Patienten- und Umweltstudien werden zum Zwecke der Datendokumentation erwähnt, aber nicht detailliert dargestellt.

##### 4.2.2.1 Untersuchungen mit Messung der Luftkonzentration am Arbeitsplatz

Im Hinblick auf ihre kognitiven Fähigkeiten (siehe auch Abschnitt 4.2.1) wurden 30 männliche Beschäftigte sowie 60 männliche Kontrollpersonen eines Wertstoff- oder Altmaterialbetriebs in Frankreich mittels MMSE, CDT und DST (Dementia Rating Scale) im Jahr 2007 untersucht. Die Staubmessung erfolgte personenbezogen bei acht der 30 Beschäftigten und mit zwei stationären Staubmessungen. Für die personenbezogene Messung wurden die Filterkassetten nahe am Gesicht positioniert, was eine selektive Sammlung von einatembaren Partikeln (E-Staub) erlaubt. Die Filter bestanden aus Quarz-Mikrofasern, was die Kontaminationsgefahr durch Aluminium stark mindert. In dem Staub wurde sowohl der lösliche als auch der unlösliche Aluminium-Anteil analysiert. Anhand eines Interviews wurden Daten zu Bildungsgrad, Arbeitshistorie, vergangener und aktueller Exposition gegen neurotoxische Stoffe, Krankheitshistorie, Verletzungen, klinischen Symptomen, Medikamenteneinnahme (inklusive aluminiumhaltiger Antazida), Alkoholkonsum, Rauchgewohnheiten, allgemeiner Gesundheit, Zufriedenheit am Arbeitsplatz und Verwendung von Atemschutz ermittelt. Als Ausschlusskriterien galten die Exposition gegen Lösungsmittel und Krankheiten, die das zentrale Nervensystem (ZNS) betreffen. Es ließen sich bei den Beschäftigten verglichen mit den Kontrollpersonen keine statistisch signifikanten neurologischen Effekte feststellen. Die Ergebnisse der Luftmessungen sowie Plasma- und Urin-Aluminium-Konzentrationen sind in Tabelle 4 dargestellt. Die personenbezogenen Luftmessungen ergaben einen Mittelwert von 2,23 (0,12–10,86) mg Al/m^3^ im einatembaren Staub (Deschamps et al. 2009). Das Probandenkollektiv wurde im Jahr 2012 in einer Follow-up-Studie nochmals untersucht, wobei 36 Beschäftigte und 36 Kontrollpersonen teilnahmen. Auch hierbei wurden bei den Untersuchungen zu kognitiven Fähigkeiten keine statistisch signifikanten Unterschiede zwischen den Exponierten und den Kontrollpersonen beobachtet. Die personenbezogene Luftmessung lag im Mittel bei 1,61 (0,13–3,13) mg Al/m^3^ in der einatembaren Staubfraktion (Deschamps et al. 2018). Die Kommission sieht die Durchführung und Auswahl der neuropsychologischen Tests als valide an. Allerdings stellen die beiden Publikationen Querschnittstudien mit Selektionsbias dar. Ob eine Adjustierung für mögliche potentielle Störfaktoren (z. B. Alkohol) erfolgte, wird nicht angegeben und die Auswertung basiert lediglich auf t-Tests, die Gruppenmittelwerte für verschiedene neurologische Testergebnisse von Exponierten und Nichtexponierten vergleichen. Anhand der Aluminiumgehalte in Luft, Urin und Plasma aus dem Kollektiv von 2007 fällt auf, dass nur bei den Urinspiegeln Unterschiede zwischen Exponierten und Nichtexponierten auftreten. Auch hatten die Kontrollpersonen im Jahr 2012 höhere Aluminium-Urinkonzentrationen als die Beschäftigten aus dem Jahr 2007 und die Beschäftigten im Jahr 2012 fast doppelt so hohe Aluminiumgehalte im Urin im Vergleich zum Kollektiv von 2007, obwohl die Luftmessung geringere Aluminiumkonzentrationen ergab. Eine Erklärung hierzu wird von den Autoren nicht gegeben. Die Validität des Biomonitoring ist in Frage zu stellen. Insgesamt sind die Studien aufgrund der beschriebenen Einschränkungen nicht geeignet, um daraus einen Grenzwert abzuleiten.

##### 4.2.2.2 Untersuchungen ohne Messung der Luftkonzentration

Untersuchungen, die seit dem Jahr 2017 publiziert wurden und mit Beschäftigten der Aluminiumindustrie durchgeführt worden sind, ohne dass Aluminium in der Luft gemessen wurde, sind im Addendum zum BAT-Wert ausführlich dargestellt (Michaelsen et al. 2025) und werden in diesem Nachtrag nicht aufgeführt. Insgesamt zeigen diese Arbeitsplatzstudien sehr hohe Aluminiumgehalte im Plasma oder Serum bei Aluminium-Exponierten und auch bei den nicht exponierten Kontrollpersonen (Meng et al. 2019 a, b; Shang et al. 2020), was auf eine hohe Hintergrundbelastung in den in China durchgeführten Studien hinweist. Die Aluminiumblutspiegel in den Studien waren nicht nur höher als der Referenzwert der Allgemeinbevölkerung in Deutschland von < 5 µg Aluminium/l Serum (HBM-Kommission 1998), sondern sie lagen bereits in einem Bereich (ab 13 µg Aluminium/l Plasma) bei denen Leistungsabnahmen in neuropsychologischen Tests beobachtet wurden und somit mit neurotoxischen Effekten zu rechnen ist (Klotz et al. 2018).

##### 4.2.2.3 Nicht bewertungsrelevante Arbeitsplatzstudien 

An Elektrolysearbeitern in China wurden verschiedene Untersuchungen zur neurotoxischen Wirkung durchgeführt und Effekte mit den Plasma-Aluminiumspiegeln korreliert (Zhang et al. 2022 b). Aufgrund der fehlenden Einteilung nach der Aluminiumexposition in der Luft kann die Studie nicht zur Ableitung eines Luftgrenzwertes herangezogen werden.

Arbeitsplatzstudien mit Exposition gegen andere Metalle werden aufgrund der Koexposition nicht zur Bewertung der neurotoxischen Wirkung herangezogen (Dick et al. 1997; Iregren et al. 2001; Mohammed et al. 2020; Shang et al. 2021).

In den Studien von Hałatek et al. (2005, 2008) und Sinczuk-Walczak et al. (2003) wurden 50 bzw. 67 Arbeiter in einer Aluminiumschmelzerei und 42 bzw. 57 Kontrollpersonen auf Veränderungen neurophysiologischer Parameter wie EEG- und ZNS-Unterschiede (k. w. A.) untersucht. Diese Parameter entsprechen nicht den Standardparametern neuropsychologischer Untersuchungen, weshalb die Ergebnisse nicht zur Bewertung herangezogen werden.

Mehrere Arbeitsplatzuntersuchungen, die nur in chinesischer Sprache verfügbar sind (Gao et al. 2021; Li et al. 2021; Qiu et al. 2016; Yang et al. 1998), werden aufgrund mangelnder Überprüfbarkeit der Methodik nicht für eine Bewertung der Neurotoxizität berücksichtigt.

In McGuire et al. (1997) wurde die Exposition lediglich mit einem Interview bestimmt.

In einer Untersuchung mit 41 Beschäftigten in der Aluminiumrecycling-Industrie ist die Teilnahmequote an der Studie nur 28 % und das untersuchte Beschäftigtenkollektiv besteht aus einer hohen Anzahl an Personen mit gesundheitlichen Problemen (Kilburn 1998; McCurdy 1998).

In einer Studie an Gold- und Uran-Minenarbeitern ist die Aluminiumexposition nicht genau angegeben (Rifat et al. 1990).

##### 4.2.2.4 Metaanalysen 

In einer Metaanalyse wurden 62 epidemiologische Studien von 1985 bis 2016 betrachtet, die eine Aluminiumexposition am Arbeitsplatz und adverse neurologische (31 Studien) sowie respiratorische Effekte (17 Studien) untersuchten. Es zeigte sich keine eindeutige Assoziation oder klare Expositions-Wirkungs-Beziehung zwischen einer Aluminiumexposition am Arbeitsplatz und dem Entstehen neurologischer, neuropsychologischer oder respiratorischer Erkrankungen (Ferguson et al. 2018).

Eine Metaanalyse von Wang et al. (2016) untersuchte, ob eine chronische Aluminiumexposition mit einem erhöhten Risiko für die Alzheimer-Krankheit assoziiert ist. Dazu wurden Arbeitsplatzstudien und Studien mit Expositionen über das Trinkwasser in Form von fünf Fall-Kontroll-Studien und drei prospektiven Kohortenstudien betrachtet (Gauthier et al. 2000; Graves et al. 1998; Gun et al. 1997; McLachlan et al. 1996; Peters et al. 2013; Rondeau et al. 2000, 2009; Salib und Hillier 1996). Die Metaanalyse ergab für die Alzheimer-Krankheit ein statistisch signifikant erhöhtes Odds Ratio von 1,71 (95-%-KI-Intervall: 1,35–2,18) und die Autoren schlussfolgern daraus, dass eine chronische Aluminiumexposition im Zusammenhang mit einem erhöhten Risiko für die Alzheimer-Krankheit steht (Wang et al. 2016). Die Untersuchung ist nur eingeschränkt aussagekräftig, da bei den Arbeitsplatzstudien keine Angaben zur Expositionshöhe gemacht wurden. Bei den Studien, die eine Exposition mit dem Trinkwasser untersuchten, ist eine Abschätzung der individuellen Aluminiumbelastung nicht möglich. Aluminium kann bei extremer Exposition eine spezifische Enzephalopathie mit einem demenziellen Syndrom verursachen, allerdings ist diese Aluminiumenzephalopathie eine eigenständige Erkrankung und nicht mit der Demenz vom Alzheimer-Typ gleichzusetzen (Klotz et al. 2017). Zusammenfassend ist anhand dieser Metanalyse ein eindeutiger Zusammenhang zwischen einer Aluminiumexposition am Arbeitsplatz oder durch eine Trinkwasserexposition und der Entstehung der Alzheimer-Krankheit insbesondere aufgrund der fehlenden Quantifizierung der Aluminium-Belastung nicht ableitbar.

##### 4.2.2.5 Reviews

In drei Reviews wird der Zusammenhang zwischen der Exposition gegen Aluminium und der Entstehung von neurodegenerativen Erkrankungen bei Minenarbeitern (Gold-, Uran-, Nickel- oder Kupferminen) diskutiert (OCRC 2020; Peters et al. 2013; Willhite et al. 2021). Aufgrund von Mischexposition ist keine zuverlässige Aussage über die Auswirkung von Aluminium möglich. In einem Review (Williams et al. 2010), bei dem anhand von 25 Reviews und 250 primärer Veröffentlichungen Risikofaktoren für die Entstehung und Progression der Alzheimer-Krankheit untersucht wurden, schlussfolgern die Autoren, dass die Datenlage für Aluminium unzureichend ist, um eine Assoziation abzuleiten. In einem Review (Santibáñez et al. 2007) wurden Studien beschrieben, die einen Zusammenhang zwischen der Alzheimer-Krankheit und Arbeitsstoffen wie Aluminium untersuchen. Für Aluminium sind drei Fall-Kontroll-Studien (Graves et al. 1998; Gun et al. 1997; Salib und Hillier 1996) genannt. Expositionshöhen werden in allen drei Studien nicht angegeben, weshalb diese nicht bewertungsrelevant sind.

##### 4.2.2.6 Patientenstudien, Umweltstudien und Fallberichte

Studien, die im Vergleich zu einer Kontrollgruppe erhöhte Aluminiumgehalte in Blut, Haar sowie Gehirn bei neurodegenerativ erkrankten Personen berichten (Exley und Clarkson 2020; Exley und Vickers 2014; Fiore et al. 2020; King et al. 2017; McLachlan et al. 2019; Mold et al. 2020), werden für die Bewertung nicht herangezogen, da unklar ist, inwiefern eine Aluminiumexposition vor der Erkrankung stattgefunden hat.

##### 4.2.2.7 Fazit

Insgesamt ist ein Kausalzusammenhang zwischen Aluminium und der Entstehung einer neurodegenerativen Erkrankung aus diesen Studien nicht herzustellen und deshalb werden diese Studien nicht für eine Grenzwertableitung berücksichtigt. Unter den Arbeitsplatzstudien, die seit dem Jahr 2017 publiziert worden sind, ist keine enthalten, bei der eine valide Luftmessung durchgeführt worden ist. Es liegen seit Erscheinen des Nachtrags (Greim 2007) keine neuen bewertungsrelevanten Daten vor. Die Studien von Deschamps et al. (2009, 2018) sind aufgrund eines Selektionsbias (Querschnittsstudien), unzureichender Auswertung, mangelnder Plausibilität der Biomonitoring-Ergebnisse sowie der Urin-Aluminiumwerte im Vergleich zu den Luft-Aluminiumwerten nicht ausreichend belastbar. Sie werden deshalb nicht zur Grenzwertableitung herangezogen. Die Studien von Buchta et al. (2003), Kiesswetter et al. (2009) und Letzel et al. (2006) zeigen keine präklinischen neurotoxischen Effekte bei Schweißern nach Inhalation von aluminiumhaltigem Schweißrauch von ca. 0,25–0,5 mg/m^3^. 

#### 4.2.3 Wirkungen auf die Knochen

Für das in Antazida (Medikamente zur Neutralisation der Magensäure) enthaltene **Aluminiumhydroxid** sind Effekte wie Osteomalazie beschrieben, jedoch treten diese nach wiederholter Gabe von sehr hohen Dosierungen (bis zu 2 g/Person und Tag) auf (Wissenschaftliche Dienste Deutscher Bundestag 2022).

#### 4.2.4 Wirkungen auf das biliäre System

Eine Arbeitsplatzuntersuchung mit 34 männlichen Beschäftigten, welche gegen Aluminium-Rauch und -Dämpfe in Höhe von 4,6 mg Al/m^3^ exponiert waren, zeigte erhöhte Werte von Gesamt- und indirektem Bilirubin sowie alkalischer Phosphatase sowie signifikant höhere Aluminiumkonzentrationen im Serum und Urin im Vergleich zu nicht exponierten Kontrollpersonen (Bogdanović et al. 2008).

### 4.3 Wirkung auf Haut und Schleimhäute 

In einer Arbeitsplatzuntersuchung an 25 Aluminiumschweißern (siehe Abschnitt 4.2.1) trat sowohl bei Personen, die über 2,5 Jahre beschäftigt waren (3/12), als auch bei Personen, die weniger als 2,5 Jahre beschäftigt waren (3/13) eine statistisch signifikante Zunahme an Konjunktivitis auf. Die Gesamtstaubkonzentration wird mit 2,8 mg/m^3 ^(Median) und die Aluminiumkonzentration mit 1,4 mg/m^3 ^(Median) angegeben (Greim 2007; Nielsen et al. 1993).

### 4.4 Allergene Wirkung

#### 4.4.1 Hautsensibilisierende Wirkung

Generell richten sich Metallallergien gegen die entsprechenden Ionen, nicht aber gegen das elementare Metall.

Obwohl die für den Epikutantest verwendeten Testkammern fast immer aus Aluminium bestehen, wurde eine kontaktallergische Reaktion gegen Aluminiumionen nach wie vor nur sehr selten beobachtet (Bruze et al. 2022, S. 20; Kullberg et al. 2020). Gelegentlich werden ringförmige Hautreaktionen auf die Testkammern beobachtet, wodurch Aluminiumallergien zufällig aufgedeckt wurden (Brodbaker und Pratt 2009; Deleuran et al. 2019; Hedberg et al. 2020; Inerot et al. 2020; King und Moffitt 2018).

Bei Verdacht auf eine Kontaktallergie gegen Aluminiumionen können Epikutantests mit elementarem Aluminium durchgeführt werden, wobei sich geringe Mengen an Aluminiumionen lösen können. Um eine Allergie gegen Aluminium und schwerlösliche Verbindungen zu detektieren, erfolgt die Testung für eine niedrigere Nachweisgrenze jedoch häufig mit löslichen Verbindungen. Diese Studien sind detailliert in der Begründung für lösliche Aluminiumverbindungen aufgeführt (Hartwig und MAK Commission 2025). Aus den Untersuchungen mit löslichen Aluminiumverbindungen geht hervor, dass eine 10%ige Testzubereitung mit Aluminiumchloridhexahydrat oder eine 12%ige Testzubereitung mit Aluminiumlactat sowie eine zusätzliche Ablesung nach sieben Tagen notwendig sind, um klinisch relevante Aluminiumallergien aufzudecken.

##### 4.4.1.1 Beruflicher Kontext 

Als mögliche berufliche Ursachen einer Sensibilisierung durch Ionen aus Aluminiummetall sowie schwerlöslichen Aluminiumverbindungen wurden der Kontakt mit **Aluminiumoxid**-haltigen Schleifmaterialien (Tosti et al. 1990) und Aluminiumstaub oder feinen Aluminiumspänen bei der Aluminiumbearbeitung (Hall 1944; Peters et al. 1998) genannt (Greim 2007). Das Vorliegen von Kontaktallergien gegen Aluminiumionen wurde auch bei Beschäftigten im Zahntechnikbereich untersucht (Lyapina et al. 2018).

In zwei neuen Epikutantestungen bei vermuteter Kontaktallergie mit möglicherweise berufsbedingtem Umgang mit Aluminium war das Testergebnis negativ bzw. wurde als nicht relevant bewertet. Bei einem Atopiker (47 Jahre) mit Hand- und Armdermatitis wurden bei der Epikutantestung an mehr als der Hälfte der Patch-Teststellen ringförmige Reaktionen beobachtet. Teilweise traten Reaktionen mit konfluierendem, ödematösem Aussehen auf, die sich von den Ringreaktionen im Hintergrund unterschieden und als echte positive Reaktionen interpretiert wurden. Bei der erneuten Testung mit einer leeren Finn-Kammer sowie Aluminiumchloridhexahydrat (2 % in Vaseline) und **Aluminiumpulver** (100 %) (beide in Kunststoffkammern getestet) wurden positive Reaktionen sowohl auf die leere Finn-Kammer als auch auf Aluminiumchloridhexahydrat beobachtet, jedoch nicht auf das Aluminiumpulver. Die Handdermatitis war bereits Jahre zuvor nach dem Umgang mit Aluminiumrohren bei seiner Arbeit aufgetreten, eine aktuelle Relevanz lag jedoch nicht vor (Kullberg et al. 2020). Es fehlen Angaben zu Reaktionsstärke und Zeitpunkt der Ablesung.

Bei einer Person (39 Jahre) mit beruflich bedingter Handdermatitis, die über 20 Jahre in der Herstellung von Jalousieschienen aus Aluminium tätig war, wurde eine Kontaktallergie auf Aluminiumionen vermutet. Die Person reagierte im Epikutantest negativ auf **elementares Aluminium** und Aluminiumchlorid (k. w. A.), jedoch positiv auf Mangan an den Tagen 2 und 4. Als Ursache für die Handdermatitis wurden manganhaltige Aluminiumverbindungen genannt (Leis Dosil et al. 2006). Eine Spätablesung an Tag 7 erfolgte nicht.

In zwei Studien aus einem Zentrum wurden Kontaktsensibilisierungen gegen Dentalmetalle bei Beschäftigten im zahnmedizinischen/zahntechnischen Bereich untersucht. Aluminiumchloridhexahydrat (2 % in Vaseline) wurde mitgetestet. Von insgesamt 287 Personen reagierten insgesamt 34 Personen am 2. Tag oder 3. Tag positiv auf Aluminiumchloridhexahydrat (Lyapina et al. 2018, 2021). Diese Studien werden zur Bewertung nicht herangezogen, da sie monozentrisch sind und der Kommission eine beruflich bedingte Sensibilisierung dieser Berufsgruppe aufgrund der fehlenden Expositionsquelle fraglich erscheint.

##### 4.4.1.2 Nicht-beruflicher Kontext

Aus einem Gesamtkollektiv von 1112 Patienten mit vermuteter Metallallergie wurden zwischen 2000 und 2009 insgesamt 1059 Personen mit **Aluminiumpulver **(100 %) getestet. Bei der Ablesung am 3. und 5. sowie 7. oder 10. Tag wurden keine positiven Reaktionen beobachtet (Davis et al. 2011). Weitere Untersuchungen zu nicht-beruflich bedingten Aluminiumallergien liegen mit löslichen Aluminiumverbindungen vor (Hartwig und MAK Commission 2025).

##### 4.4.1.3 Implantate

Des Weiteren wurden Epikutantests mit Aluminiumverbindungen bei Unverträglichkeitsreaktionen auf metallhaltige Implantate durchgeführt, wobei in verschiedenen Kollektiven bei der Testung mit **elementarem Aluminium**, **Aluminiumhydroxid** (10 % in Vaseline) oder **Aluminium** unbekannter Testzubereitung an insgesamt 210 Personen keine Reaktionen beobachtet wurden (Kręcisz et al. 2006, 2012; Pautasso et al. 2023; Zeng et al. 2014). In keiner der Studien wurden die Testergebnisse nach 7 Tagen abgelesen.

In einer Studie wurde bei 220 Erwachsenen mit geplanter Knie-Arthroplastie ein Lymphozyten-Transformationstest mit **Aluminiumoxid** (k. w. A.) (5 und 10 µg/ml) durchgeführt. Bei drei Personen (1,3 %) wurde eine „true hypersensitivity“ festgestellt (Stimulationsindex SI ≥ 5) (Boutefnouchet et al. 2022).

In einem Fall wurde über das Auftreten von Infiltrationen in 20 Jahre alten, aluminiumhaltigen Tätowierungen berichtet, welche gleichzeitig mit systemischen Symptomen nach Einsatz eines metallhaltigen Implantats über mehrere Monate auftraten. Nach der chirurgischen Entfernung der Metallosteosynthese-Implantate aus der Wirbelsäule klangen die Symptome innerhalb einer Woche ab. Die Epikutantest-Ergebnisse mit elementarem **Aluminium** (100 %) sowie **Aluminiumhydroxid** (10 % in Vaseline) waren negativ (2. Tag, 4. Tag, Arm und Rücken; positive Reaktionen auf Titan) (de Cuyper et al. 2017). Eine Ablesung am 7. Tag erfolgte nicht.

##### 4.4.1.4 Impfungen

Die meisten Studien betreffen nach wie vor eine Aluminiumsensibilisierung in Folge der Injektion von Impfstoffen, die neben löslichen Aluminiumverbindungen auch das schwerlösliche Aluminiumoxyhydroxid sowie gemischte Aluminiumsalze enthalten können (z. B. Bergfors et al. 2019; Bruze et al. 2008; Danielsson und Eriksson 2021; Deleuran et al. 2019; Goiset et al. 2018; Inerot et al. 2020; Kullberg et al. 2020; Laera et al. 2023; Wahl et al. 2014; Xará et al. 2023) oder von Zubereitungen zur allergenspezifischen Immuntherapie (Hyposensibilisierung) (Bruze et al. 2008; Hindsén 2005; Netterlid et al. 2013). Die Aluminiumverbindungen werden hierbei als Adjuvans eingesetzt. Betroffen sind vor allem Kinder und Jugendliche, seltener Erwachsene. Die Befunde zu Impfnebenwirkungen bei Kindern und zu Implantaten weisen auf systemische Verfügbarkeit und mögliche Sensibilisierung durch Ionen aus Aluminium(verbindungen) hin. Sie sind jedoch nicht direkt auf die Situation am Arbeitsplatz übertragbar, untermauern aber das Wirkprinzip der Materialien und vervollständigen das Gesamtbild.

##### 4.4.1.5 Fazit

Unter Berücksichtigung der weiten Verbreitung von Aluminium, das unter anderem routinemäßig bei der Epikutantestung als inertes Material für „Finn Chambers“ verwendet wird, sprechen die wenigen Fallberichte mit nachgewiesener Sensibilisierung gegen Aluminiumionen nach wie vor für eine geringe allergene Potenz.

#### 4.4.2 Atemwegssensibilisierende Wirkung

Trotz zahlreicher Fälle von Aluminose nach hoher Aluminiumexposition finden sich keine neuen Hinweise auf eine atemwegssensibilisierende Wirkung. Auch das gehäufte Auftreten obstruktiver Atemwegserkrankungen („Potroom-Asthma“) bei Beschäftigten in Aluminiumschmelzbetrieben konnte nicht auf eine immunologische Genese zurückgeführt werden, zumal in diesem Bereich eine Mischexposition auftritt (Desjardins et al. 1994; Kongerud und Søyseth 2014).

Die Ergebnisse der bereits im Nachtrag (2007) dargestellten einzelnen Fallberichte (Burge et al. 2000; Park et al. 1996; Vandenplas et al. 1998) deuten darauf hin, dass eine Sensibilisierung der Atemwege bei Exposition gegen schwerlösliche Aluminiumverbindungen auftreten kann, aber die Ergebnisse größerer Studien (Beach et al. 2001; Fritschi et al. 2001) legen nahe, dass sie selten ist.

Es liegen einige neue Studien zur Prävalenz von Asthma bei Beschäftigten in der Aluminiumherstellung vor, aus denen jedoch aufgrund fehlender allergologischer Untersuchungen keine Hinweise auf eine sensibilisierende Wirkung von Aluminium oder Aluminiumverbindungen abgeleitet werden können (Abramson et al. 2010; Barnard et al. 2004; Benke et al. 2008; Cvejanov Kezunović 2008; Donoghue et al. 2011; Gulati et al. 2009; Kudaeva et al. 2016; Leira et al. 2005; van Rooy et al. 2011; Sjåheim et al. 2007; Taiwo et al. 2006).

Es wurde der Fall einer 41-jährigen Frau beschrieben, welche nach beruflicher Exposition gegen aluminiumhydroxidhaltige Pulverfarbe über fünf Jahre Atemwegssymptome (trockener Husten, Dyspnoe und Keuchen) entwickelte. In ihrem beruflichen Umfeld war sie indirekt Pulverfarbe ausgesetzt, die **Aluminiumhydroxid** (20–25 %) enthielt. Der Lungenfunktionstest zeigte eine minimale Obstruktion der Atemwege mit einer forcierten exspiratorischen Einsekundenkapazität (FEV1) von 88 % Soll, einer forcierten Vitalkapazität (FVC) von 116 % Soll und einem FEV1/FVC-Quotienten von 0,66. Die Konzentration des fraktionierten exhalierten Stickstoffmonoxids (FeNO), die während einer Arbeitsperiode gemessen wurde, war normal (4,15 µl/m^3^). Im Methacholintest wurde ein Abfall der FEV1 um 27 % (von 2,4 Liter bei Studienbeginn auf 1,7 Liter bei einer kumulativen Dosis [PD20] von 80 µg Methacholin 1 %) in Kombination mit Asthmasymptomen beobachtet. Bei der Durchführung eines spezifischen inhalativen Provokationstests mit der Pulverfarbe (Expositionsdauer: 10 Sekunden, 1 Minute, 5 Minuten) wurden nach fünfminütiger Exposition Asthmasymptome und ein Abfall der FEV1 um 16 % beobachtet. Nachdem die Beschäftigte ihren Arbeitsplatz innerhalb der Firma wechselte, klangen die Symptome ab. In Abwesenheit anderer Atemwegsallergene wie Triglycidylisocyanurat oder organischer Säureanhydride in Pulverlacken vermuten die Autoren Aluminiumhydroxid als das wahrscheinlichste Allergen (Tiotiu et al. 2019). Da der Verlauf der Vitalkapazität nicht berichtet wurde und auch (mangels Ganzkörperplethysmographie) keine Atemwegswiderstände gemessen wurden, bleibt unklar, ob es sich tatsächlich um eine substanzinduzierte Obstruktion handelt oder ob lediglich die Anstrengungsbereitschaft der Probandin während der Exposition rückläufig war. Die Studie ist daher von sehr begrenztem Aussagewert und kann somit nicht gesichert im Sinne einer substanzinduzierten Atemwegsobstruktion interpretiert werden.

Die Auswirkungen einer kurzzeitigen kontrollierten Ganzkörper-Exposition gegen **Aluminiumoxid **(nicht näher spezifiziert) auf die Lungenfunktion und Entzündungsmarker wurden bei 15 gesunden Freiwilligen untersucht. Die Personen wurden zwei Stunden, einschließlich 2 × 15 Minuten Fahrradergometerarbeit (75 Watt) gegen 3,8–4,0 mg Aluminiumoxid/m^3^ exponiert. Im Sputum wurden 24 Stunden nach der Exposition ein Anstieg von Neutrophilen und IL-8 sowie eine Zunahme der Expression von Genen, die eine Rolle bei der Regulierung beispielsweise von Zell-Zell-Signalübertragung spielen, festgestellt (Sikkeland et al. 2016).

Eine Studie an Schweißarbeitern (Lillienberg et al. 2008) sowie ein Fallbericht einer Person, die mit Maurerarbeiten beschäftigt war (Burge et al. 2012), können aufgrund von Koexposition gegen andere Materialien nicht zur Bewertung herangezogen werden.

### 4.5 Reproduktionstoxizität 

#### 4.5.1 Fertilität

Seit dem Nachtrag (Greim 2007) sind keine weiteren Studien zur Fertilität veröffentlicht worden.

Eine hohe Aluminiumkonzentration in Spermien korrelierte mit einer verminderten Spermienmotilität. Die Blei- und Cadmiumkonzentrationen waren in dieser Studie niedrig und zeigten keinen Einfluss auf die Samenqualität (Greim 2007; Hovatta et al. 1998).

#### 4.5.2 Entwicklungstoxizität

Im Rahmen einer Kohortenstudie (Workers Health in Apprenticeship Trades – Metal and Electrical (WHAT-ME) Study) wurden 447 Frauen, die eine Ausbildung als Schweißerin antraten, alle sechs Monate bis zu fünf Jahre lang einem Follow-up mit detaillierter Befragung unterzogen und jede Schwangerschaft aufgezeichnet. Die Exposition gegen Metalle (Aluminium, Chrom, Mangan, Nickel) und Partikel wurde mittels validierten Expositionsalgorithmen geschätzt, die den Schweißprozess, das Basismetall und die Verbrauchsmaterialien beim Empfängnistag widerspiegeln sollten. Es kam zu 122 Schwangerschaften bei 90 Schweißerinnen. Von den 122 Schwangerschaften ergaben 91 eine Lebendgeburt und 31 einen fetalen Verlust (27 Fehlgeburten und vier Totgeburten). In einer bivariaten Analyse zeigte sich, dass für Schweißerinnen mit mehr als einer Schwangerschaft das Risiko eines fetalen Verlusts mit der geschätzten Aluminiumluftkonzentration (und zu einem geringeren Grad auch Nickel und Partikel) zunahm (Odds Ratio OR = 1,52; 95-%-Konfidenzintervall: 1,04–2,24) (Galarneau et al. 2022). Die geringe Anzahl untersuchter Schweißerinnen und die fehlenden Messwerte sowie die anzunehmende Mischexposition lassen eine abschließende Bewertung des Einflusses von Aluminium auf den Verlust des Fetus nicht zu.

Eine prospektive Kohortenstudie aus dem Zeitraum Juli bis September 2015 umfasste 703 Mutter-Kind-Paare aus der Guangxi-Geburtskohortenstudie (China). Das Alter der Mütter lag bei 28,68 ± 4,33 Jahren. Die Aluminiumkonzentrationen im Serum lagen bei 85 % der Proben über der Nachweisgrenze (0,33 µg/l); das 95. Perzentil betrug 224,7 µg/l. Im Multi-Metallmodell ergab sich eine negative Assoziation zwischen der pränatalen Aluminiumkonzentration im Serum der Mütter und den Entwicklungsquotienten für Feinmotorik, Anpassung, Sprache und Sozialverhalten der Kinder. Den Autoren zufolge sind Limitierungen der Studie die Einzelmessung der Metalle und nicht berücksichtigte Confounder wie Bildung und genetische Faktoren (Liu et al. 2022).

Eine Querschnittsuntersuchung in einer Aluminiumschmelzerei ergab, dass die Exposition gegen mittlere zeitgewichtete Luftkonzentrationen für Aluminiumoxid von 0,091 (0,02–0,411) mg/m^3^ und für Aluminium von 2,8226 (0,073–8,3) mg/m^3^ nicht für eine Zunahme kongenitaler Anomalien verantwortlich war (Sakr et al. 2010).

In einer Fall-Kontroll-Studie in Oaxaca City, Mexiko, wurden acht Neugeborene mit Neuralrohrdefekten (sechs Mädchen, zwei Jungen) und 23 Neugeborene ohne einen derartigen Defekt (acht Mädchen, 15 Jungen) aus einem Krankenhaus miteinbezogen. Die Aluminiumkonzentration im Haar lag bei den Kindern mit den Neuralrohrdefekten bei 152,8 ± 51,1 μg/g und bei den Kontrollen bei 76,2 ± 27,9 μg/g (p < 0,005; Ramírez-Altamirano et al. 2012). Der Zeitraum, in dem die Fälle und Kontrollen rekrutiert wurden, ist nicht angegeben. Auch fehlt eine Aussage darüber, wie die Kontrollen ausgewählt wurden und über die Hintergrundwerte der Aluminiumkonzentration in den Haaren von Personen aus der Region. Zudem ist die Fallzahl gering. Die Studie ist daher nicht zur Bewertung von Aluminiumeffekten geeignet.

Umweltepidemiologische Studien berichten über Assoziationen zwischen Aluminiumluftkonzentrationen und erniedrigtem Geburtsgewicht (Bell et al. 2010) bzw. vorzeitiger Plazentaablösung (Ibrahimou et al. 2017). Auch Assoziationen zwischen erhöhten Aluminiumkonzentrationen im Serum der Mütter und dem Risiko von fetalen Neuralrohrdefekten (Liu et al. 2021) bzw. angeborenen Herzdefekten (Liu et al. 2016) werden beschrieben. Eine Untersuchung ergab eine Assoziation zwischen hohen Aluminiumkonzentrationen im Urin (geometrischer Mittelwert 6,14 µg/l; 95-%-Konfidenzintervall: 3,80–9,90 µg/l) von Müttern beduinisch-arabischer Herkunft und dem Auftreten einer vorzeitigen Geburt sowie von Fehlbildungen (Karakis et al. 2021). Eine weitere Untersuchung dieser Arbeitsgruppe zeigte, dass bei den Neugeborenen von Müttern beduinisch-arabischer Herkunft mit hohen Aluminiumkonzentrationen im Urin (n = 15, > 9 µg/l; Vergleichsgruppe n = 43, < Nachweisgrenze von 9 µg/l) und stärkeren Hinweisen auf oxidativen Stress im Nabelschnurblut eine höhere Wahrscheinlichkeit für eine geringere Körpergröße bestand (Karakis et al. 2014). Bei jamaikanischen Neugeborenen hingegen wurde kein Zusammenhang zwischen der Aluminiumkonzentration im Nabelschnurblut und der Körpergröße gefunden (Rahbar et al. 2015). Die wenigen Untersuchungen geben kein konsistentes Bild. Die Ursache für eine vorzeitige Plazentaablösung ist nicht bekannt, es gibt mehrere Risikofaktoren dafür. Einen Zusammenhang mit einer Exposition gegen Aluminium herzustellen, ist aufgrund des unbekannten Entstehungsmechanismus spekulativ.

Aluminiumhydroxid und Aluminiumphosphat werden therapeutisch als Antazida eingesetzt. Obwohl gelegentlich diskutiert wird, dass aus Antazida resorbiertes Aluminium zu funktionellen Störungen im Zentralnervensystem und in den Nieren der Feten führen könnte, haben sich dafür klinisch bisher keine Hinweise ergeben (Schaefer et al. 2011).

### 4.6 Genotoxizität

Eine Studie zur Untersuchung von DNA-Schäden und dem Gehalt unterschiedlicher Metalle in Urin und Blut wurde an 30 männlichen Schweißern (Alter: 42 ± 11,9 Jahre; 40 % Raucher) und einer Kontrollgruppe von 22 Nicht-Schweißern (Alter: 43,9 ± 10,9 Jahre; 55 % Raucher), angepasst nach Alter, Geschlecht und Raucherstatus durchgeführt. Die Konzentrationen der Metalle im Blut (abgeschätzt aus Abbildung, 25. und 75. Perzentil-Werte) wurden am Wochenanfang vor Arbeitsbeginn für die Schweißer (ca. 30 bzw. 100 µg Al/l) und die Kontrollgruppe (ca. 40 bzw. 75 µg Al/l) und am Ende der Arbeitswoche nur für die Schweißer (ca. 25 bzw. 110 µg Al/l) bestimmt. Die Urinuntersuchung erfolgte für alle Gruppen am Montag zu Arbeitsbeginn und nur für die Schweißer am Donnerstag am Ende des Arbeitstages. Bei 20 Schweißern waren am Ende der Arbeitswoche DNA-Schäden (Comet-Assay) in den Lymphozyten statistisch signifikant erhöht, bei sechs Schweißern unverändert und bei vier statistisch signifikant vermindert im Vergleich zu den Werten am Wochenanfang. Im Blut wurde kein statistisch signifikanter Unterschied zwischen den Aluminiumkonzentrationen der Schweißer und denen der Kontrollpersonen gemessen. Im Urin waren die Aluminiumkonzentrationen am Beginn und Ende der Arbeitswoche statistisch signifikant erhöht im Vergleich zu den Kontrollpersonen. Die Konzentrationen im Urin (abgeschätzt aus Abbildung, 5. und 95. Perzentil) lagen zu Wochenbeginn bei 5 bzw. 25 µg/g Kreatinin und am Ende der Arbeitswoche bei 5 bzw. 35 µg/g Kreatinin. Die Aluminiumkonzentration im Urin korrelierte nicht mit der Zunahme an DNA-Schäden. Eine statistisch signifikante positive Korrelation (substanzspezifisch berechneter Spearman-Rangkorrelationskoeffizient) ergab sich für DNA-Schäden und die Aluminiumkonzentration im Blut zum Wochenbeginn. Die Schweißer waren auch gegen Cadmium, Mangan, Zink, Chrom, Cobalt, Nickel und Blei exponiert. Eine statistisch signifikant positive Korrelation ergab sich auch für DNA-Schäden und die Cobalt-, Nickel- und Bleikonzentration im Blut zum Wochenbeginn (Botta et al. 2006). Die Konzentrationen im Urin lagen unterhalb des BAT-Wertes von 50 µg Al/g Kreatinin (Klotz et al. 2018). Die hohen Blutwerte, im Vergleich zum Referenzwert von ≤ 5 µg Al/l Serum, sind in Kenntnis der Urinwerte jedoch nicht plausibel und wahrscheinlich auf eine Kontamination der Blutentnahmeröhrchen zurückzuführen, was ein bekanntes Problem bei der Probenahme darstellt. Zudem liegt eine Koexposition mit anderen Metallen vor. Insgesamt ist die Studie somit nicht belastbar, um einen Zusammenhang von Aluminium und einer genotoxischen Wirkung festzustellen.

In einer Studie an 143 gesunden Personen, die an der Reinigung der spanischen Küste nach einem Öltanker-Unglück beteiligt waren, wurden die Konzentrationen an Aluminium, Cadmium, Nickel, Blei und Zink im Blut jeweils morgens vor Beginn der Arbeit bestimmt. Mit den Leukozyten wurden Mikronukleustests, Comet-Assays und Untersuchungen zu SCE (Schwesterchromatidaustausch) durchgeführt. Die Aluminium-Konzentrationen im Blut betrugen 9,94 ± 6,88 µg/l bei Männern und 10,42 ± 7,42 µg/l bei Frauen. Eine statistisch signifikante Korrelation zwischen der Aluminiumkonzentration im Blut und der Induktion von Genotoxizität in den Tests ergab sich nicht (Pérez-Cadahía et al. 2008).

In einer Querschnittstudie wurden bei 96 männlichen Beschäftigten einer Aluminium-Schmelzerei (Alter: 36,41 ± 11,5 Jahre; 50 % Raucher) und einer Kontrollgruppe von 96 männlichen Beschäftigten, die nicht in der Schmelzerei tätig waren (Alter: 34,8 ± 11,1 Jahre; 48 % Raucher), angepasst nach Alter und Raucherstatus, die Konzentrationen von Aluminium im Blutserum nach achtstündigem Fasten (k. w. A.), die Konzentration von 8-OHdG (8-Hydroxydesoxyguanosin) im Urin und die DNA-Schädigung im Comet-Assay an Blutlymphozyten bestimmt. Im Blutserum war die Aluminiumkonzentration bei den Schmelzern (Median: 10 µg/l, Bereich: 4–30 µg/l) im Vergleich zur Kontrollgruppe (0,3 µg/l; 0,01–2,1 µg/l) statistisch signifikant erhöht. Die 8-OHdG-Konzentrationen im Urin lagen im Median bei 7,6 ng/mg Kreatinin (2,7–17,2 ng/mg Kreatinin) für die Aluminium-Schmelzer und bei 3,5 ng/mg Kreatinin (2–7 ng/mg Kreatinin) für die Kontrollgruppe. Im Comet-Assay war die Anzahl an DNA-Schäden bei den Schmelzern im Vergleich zur Kontrollgruppe statistisch signifikant erhöht. Die Aluminiumkonzentration im Serum korrelierte mit der Dauer der Beschäftigung, mit der Induktion von DNA-Schäden, mit der Konzentration an 8-OHdG im Urin und der Abnahme der antioxidativen Kapazität im Blut. Der geometrische Mittelwert von Aluminiumstaub in der Luft an verschiedenen Stellen der Schmelzerei (vierstündige Sammlung während der Arbeitsschicht, k. w. A.) betrug 0,5 ± 0,02 mg/m^3^ (0,07–0,37 mg/m^3^) (Samir und Rashed 2018). Es sind keine Einzeldaten zu den Experimenten dargestellt und die Angaben in Tabelle und Abstract zur antioxidativen Kapazität im Blut sind widersprüchlich, was die Nachvollziehbarkeit und Validität einschränkt. Eine Koexposition gegen andere Metalle ist nicht auszuschließen, sodass das vermehrte Auftreten an DNA-Schäden bei den Schmelzern nicht allein auf die Belastung mit Aluminium zurückgeführt werden kann.

Aufgrund fehlender Quantifizierung der Exposition bzw. Belastung kann die Studie von Gundy et al. (2013) nicht zur Bewertung der Genotoxizität von Aluminium herangezogen werden. Ebenfalls wird die Umweltstudie von Hou et al. (2014) nicht zur Bewertung herangezogen, da sie keine Aussage zur spezifischen Wirkung von Aluminium zulässt.

**Fazit Genotoxizität beim Menschen**: Es ergeben sich keine Hinweise auf eine genotoxische Wirkung von Aluminium beim Menschen**.**

### 4.7 Kanzerogenität

#### 4.7.1 Fall-Kontroll-Studien

Es liegen keine Fall-Kontroll-Studien mit schwerlöslichen Aluminiumverbindungen vor.

#### 4.7.2 Kohortenstudien

Kohortenstudien mit Arbeitern von Bauxit-Minen, Aluminium-Raffinerien und Aluminium-Schmelzöfen sind ausführlich in der Bewertung von Korund beschrieben. Auf der Basis dieser epidemiologischen Daten kann das kanzerogene Potenzial schwerlöslicher Aluminiumverbindungen aufgrund unvollständiger Informationen vor allem zur Exposition und dem Auftreten von Mischexpositionen nicht bewertet werden (Hartwig und MAK Commission 2019).

## 5 Tierexperimentelle Befunde und In-vitro-Untersuchungen

### 5.1 Akute Toxizität 

#### 5.1.1 Inhalative Aufnahme

##### 5.1.1.1 Mikroskalige Partikel

Je fünf weibliche und männliche Wistar-Ratten wurden vier Stunden lang gegen **Aluminiumoxid** („fumed alumina", hergestellt durch Verbrennen von Aluminiumchlorid, MMAD: 2,31–2,85 µm; geometrische Standardabweichung (GSD): 2,97–3,22) in einer Konzentration von 2300 mg/m^3^ ganzkörperexponiert und nach 14 Tagen untersucht. Es zeigten sich keine bewertungsrelevanten Auffälligkeiten. Die 4-Stunden-LC_50_ war > 2300 mg/m^3^ (ECHA 2023 c).

Gegen ein Aerosol von **Aluminiumoxid** („fumed alumina", γ-Modifikation, k. A. zur Partikelgröße) in Konzentrationen von 0, 5060, 5880, 6280 oder 8220 mg/m^3^ wurden zehn männliche Charles-River-Ratten pro Gruppe eine Stunde lang ganzkörperexponiert und nach 14 Tagen untersucht. In der höchsten Konzentrationsgruppe starb die Hälfte der Tiere. Vergiftungserscheinungen während der Exposition waren Piloarrektion oder Schnappatmung. Bereits ab der geringsten getesteten Konzentration traten in der Nachbeobachtungszeit vereinzelt Haarverlust, verkrampfte Körperhaltung und Verkrustungen um die Nase auf, welche am Ende der Beobachtungszeit reversibel waren. Die 1-Stunden-LC_50_ betrug 7600 mg/m^3 ^(ECHA 2023 c).

Je zehn männliche und weibliche Sprague-Dawley-Ratten pro Gruppe wurden vier Stunden lang gegen 5090 mg/m^3^ eines Aerosols exponiert, welches zu 75 % aus **Aluminiumoxid** und zu 25 % aus **Aluminiumhydroxid** bestand (MMAD: 4,6 µm/GSD: 3,12). Die Effekte wurden 72 Stunden nach der Exposition untersucht. Exponierte Tiere zeigten Piloarrektion, verminderte Aktivität und Ptosis. Mortalität trat nicht auf. Die 4-Stunden-LC_50_ war > 5090 mg/m^3^ (ECHA 2023 c).

Je elf weibliche BALB/c-Mäuse wurden eine Stunde lang gegen 8 mg suspendierten Metallstaub/m^3^ (50 mg Al/g, 0,4 mg Al/m^3^, Partikelgröße: 90 % < 150 µm, 50 % < 81 µm, 10 % < 17 µm) aus der Aluminiumproduktion exponiert. Die Tiere zeigten nach 24 Stunden eine statistisch signifikante Zunahme an alveolärem Kollaps, einen Einstrom polymorphkerniger Zellen in das Lungenparenchym und eine Beeinträchtigung der Lungenmechanik (Mazzoli-Rocha et al. 2010). Aufgrund der geringen Aluminiumkonzentration im Staub ist fraglich, ob die Befunde durch Aluminium ausgelöst wurden und die Studie ist daher nicht für die Beurteilung der Aluminiumtoxizität geeignet.

Es wurden je sechs F344-Ratten gegen 0, 10, 50, 100, 200 oder 1000 mg **Aluminiumstaub**/m^3^ (Partikelgröße 2,62–3,28 µm) vier Stunden lang ganzkörperexponiert. Die Tiere wurden 24 Stunden, 14 Tage, drei und sechs Monate nach Exposition untersucht, wobei auch eine Analyse der BALF durchgeführt wurde. Akute toxische Symptome traten nicht auf. In der BALF zeigte sich eine Zunahme der alkalischen Phosphatase (ALP) ab 50 mg Al/m^3^ nach 24 Stunden, 14 Tagen und drei Monaten, nicht aber nach sechs Monaten. Die Konzentration der LDH stieg ab 100 mg Al/m^3^ nach 24 Stunden, 14 Tagen und drei Monaten an, der Proteingehalt nahm ab 100 mg Al/m^3^ nach 14 Tagen sowie bei 200 mg Al/m^3^ nach 24 Stunden, 14 Tagen und drei Monaten zu. Die Glucose-6-phosphat-Dehydrogenase zeigte bei 1000 mg Al/m^3^ eine Zunahme nach 24 Stunden und 14 Tagen. Die Makrophagen, Lymphozyten und polymorphkernigen Neutrophilen in der BALF waren nach vier Stunden unverändert (Thomson et al. 1986).

##### 5.1.1.2 Ultrafeine Partikel

Zwölf weibliche Wistar-Ratten wurden einmalig vier Stunden lang gegen in Wasser suspendiertes **ultrafeines Aluminiumoxid** (Partikelgröße 13 nm, γ/δ-Modifikation) in einer Konzentration von 20,0–22,1 mg/m^3^ nur über die Nase exponiert. Es ließen sich verglichen mit der Kontrolle keine klinischen Symptome, Veränderungen des Körpergewichts sowie des Gesamtproteins oder der LDH in der BALF beobachten. Die histopathologische Untersuchung der Lunge zeigte interstitielle und entzündliche Läsionen (Bourgois et al. 2021). Die LC_50_ für ultrafeines Aluminiumoxid ist > 20,0–22,1 mg/m^3^.

Acht bis zehn weibliche BALB/cj-Mäuse pro Gruppe wurden eine Stunde lang nur über den Kopf gegen staubförmiges **ultrafeines Aluminiumoxid** (Partikelgröße 13,6 ± 8,4 nm, k. A. zur Modifikation) in Konzentrationen von 0, 23, 94 oder 235 mg/m^3^ exponiert und nach 24 Stunden sowie 13 Wochen untersucht. Es zeigten sich keine Mortalität, keine Zunahme an Neutrophilen, Lymphozyten, Makrophagen oder Gesamtprotein in der BALF und keine histopathologischen Veränderungen in der Lunge. Die LC_50_ ist > 235 mg Aluminiumoxid/m^3^ (ECHA 2023 c; Larsen et al. 2016).

#### 5.1.2 Orale Aufnahme

In Tabelle 6 sind die verfügbaren Studien zur einmaligen oralen Aufnahme und die zugehörigen LD_50_-Werte schwerlöslicher Aluminiumverbindungen dargestellt.

**Tab. 6:** Untersuchungen zur akuten Toxizität nach oraler Verabreichung schwerlöslicher Aluminiumverbindungen

**Spezies,** **Stamm, ** **Anzahl pro Gruppe **	**Dosis**	**LD_50_** **[mg Al/kg KG]**	**Literatur**
Ratte, Sprague Dawley, 5 ♂, 5 ♀	**Al_2_O_3_** (k. w. A.) 1000, 1590, 2510, 3980, 6310, 10 000, 15 900 mg/kg KG (529, 842, 1328, 2106, 3340, 5293, 8415 mg Al/kg KG)	> 8415	Hazleton Laboratories 1969
Ratte, Wistar, 10 ♂, 10 ♀	**Al_2_O_3_** (k. w. A.) 10 000 mg/kg KG (5293 mg Al/kg KG)	> 5293	Degussa Frankfurt 1979 a
Ratte, Wistar, 6 ♀, OECD TG 423	**Al(OH)_3_** 2000 mg/kg KG (692 mg Al/kg KG)	> 619	LAB Research Ltd. 2009 a

Al(OH)_3_: Aluminiumhydroxid; Al_2_O_3_: Aluminiumoxid; TG: Prüfrichtlinie (Test Guideline)

In einer weiteren Studie erhielten fünf männliche ICR-Mäuse einmalig **γ-Aluminiumoxyhydroxid** und **γ-Aluminiumoxid** per Trinkwasser in den Dosen von 0, 5 und 10 mg/kg KG. Es ließ sich für γ-Aluminiumoxyhydroxid bei 10 mg/kg KG eine Abnahme an weißen Blutzellen, eine Zunahme an Neutrophilen und IL-8 sowie ab 5 mg/kg KG eine Abnahme an Thrombozyten im Blut erkennen. Für γ-Aluminiumoxid ließ sich bei 10 mg/kg KG eine Zunahme der weißen Blutzellen und ab 5 mg/kg KG ein Anstieg an IL-8 beobachten (Park et al. 2016).

#### 5.1.3 Dermale Aufnahme

Hierzu liegen keine Angaben vor.

#### 5.1.4 Intratracheale Gabe

Jeweils zehn männlichen ICR-Mäusen pro Gruppe wurden verschiedene ultrafeine Aluminiumoxid- und Aluminiumoxyhydroxid-Partikel intratracheal einmal in den Dosen von 0; 2,5 und 5 mg/kg KG appliziert. Für die niedrige Dosisgruppe ließ sich eine Zunahme an Neutrophilen, weißen Blutzellen und Basophilen im Blut und von Lymphozyten in der BALF beobachten. In der hohen Dosisgruppe stiegen der Gehalt an Neutrophilen und Eosinophilen im Blut und in der BALF die Gesamtzellzahl, Basophilen und Eosinophilen an, während die Makrophagen abnahmen. Weiterhin waren Interleukin-6, MIP-1a, MCP-1a (monocyte chemotactic protein) und GM-CSF (granulocyte-macrophage colony-stimulating factor) erhöht. Die LDH-Werte in der BALF waren bei allen Expositionsgruppen höher (Park et al. 2018).

### 5.2 Subakute, subchronische und chronische Toxizität

#### 5.2.1 Inhalative Aufnahme

##### 5.2.1.1 Mikroskalige Aluminiumverbindungen

Hierzu liegen seit dem Nachtrag von 2007 (Greim 2007) keine neuen Studien vor. In der Studie von Gross et al. (1973) wurde das Material nicht charakterisiert, zudem lässt sich nicht zuordnen, ob das unlösliche Korund enthalten ist. Die Studie wird deshalb nicht zur Bewertung herangezogen.

##### 5.2.1.2 Ultrafeine Aluminiumverbindungen 

Es liegen insgesamt sechs Inhalationsstudien vor (Adamcakova-Dodd et al. 2012; Bayer Schering Pharma AG 2008, 2009; Bourgois et al. 2021; Kim et al. 2018; Li et al. 2017; Pauluhn 2009 b; Zhang et al. 2015), bei zwei davon (Li et al. 2017; Zhang et al. 2015) fehlen Angaben zur Modifikation von **Aluminiumoxid**. Die Nachfragen an die Autoren blieben ohne Antwort. Die Angaben von Li et al. (2017) und Zhang et al. (2015) entsprechen nach Abgleich mit dem Produktkatalog des Herstellers (PlasmaChem GmbH 2022) > 90 % α-Al_2_O_3_, dem unlöslichen Korund, welches nicht Bestandteil dieser Begründung ist. Die verbleibenden Inhalationsstudien sind in Tabelle 7 dargestellt.

**Tab. 7:** Toxizität schwerlöslicher ultrafeiner Aluminiumverbindungen nach wiederholter inhalativer Exposition

**Spezies,** **Stamm, ** **Anzahl pro Gruppe**	**Exposition**	**Befunde^a)^**	**Literatur**
Ratte, Wistar, 12 ♂ (davon 6 für BALF, 3 für Histopathologie, 3 für Al-Quantifizierung in Lunge)	**Al_2_O_3_** (γ/δ-Modifikation), 13 nm, MMAD: 0,38 µm (Agglomerate), **4 d**, 0; 22,1 mg/m^3 ^(0; 11,7 mg Al/m^3^), 4 h/d, Reinheit: 99,8 %, nur über die Nase, Nebel (10 g Al_2_O_3_/l Suspension)	**bei 11,7 mg Al/m^3^**: BALF: Gesamtprotein ↑, LDH ↑, Gesamtzellzahl ↑, polymorphkernige Neutrophile ↑, TNF-α ↑, IL-1β ↑, GRO/KC ↑, MIP-2 ↑, 8-Isoprostan (Lipidperoxidations-Marker) ↓; Lunge: Entzündungsläsionen im interstitiellen u. alveolären Bereich, deutliche Ödeme der Alveolarwände, leichte Anzeichen bronchialer (Prä)-Exfoliation, vaskuläre Ödeme, vereinzelte lokalisierte entzündliche Bereiche, bestehend aus polymorphkernigen Zellen u. Lymphozyten; Lungenbeladung: ca. 260 μg Al/g Gewebe (p < 0,0001); keine weiteren Organe untersucht	Bourgois et al. 2021
Ratte, Sprague-Dawley, 16 ♂, 16 ♀ (davon jeweils 8 ♂, 8 ♀ für Nachbeobachtung, Histopathologie)	**Al_2_O_3_** (γ-Modifikation), 11,94°nm (berechnet); lt. Hersteller < 50 nm, MMAD: 0,378; 0,48; 0,515°µm (GSD: 1,7; 4,61; 3,28), **28 d**, 0; 0,2; 1; 5 mg/m^3 ^(0; 0,1; 0,5; 2,6 mg Al/m^3^), 6 h/d, 5 d/Wo, Reinheit: k. A., nur über die Nase, Nebel, 28 d Nachbeobachtung	**ab 0,1 mg Al/m^3^**: BALF: polymorphkernige Leukozyten ↑ (2,5; 6,6; 20,6; 71,2 %), Lunge: Grad minimal, 0; 0,2; 1; 5 mg/m^3^: Entzündungszellen** **↑ (0/8, 1/8, 0/8, 1/8); Akkumulation alveolärer Makrophagen (bei 0/8, 1/8, 0/8, 4/8), granulomatöse Entzündung (bei 0/8, 1/8, 0/8, 0/8); Al-Gehalt im Lungengewebe nimmt konzentrationsabhängig zu (0,01; 0,10; 0,59; 2,29 µg/g Lunge; nach 28 d Nachbeobachtung: 0,01; 0,08; 0,51; 1,85 µg/g); **ab 0,5 mg Al/m^3^ (NOAEC der Autoren)**: BALF: Gesamtzellzahl ↑; **ab 2,6 mg Al/m^3^**: abs. u. rel. Lungengew. ↑ (11,5 bzw. 10,6 %), BALF: Lymphozyten ↑, LDH ↑, IL-6 ↑, TNF-α ↑	Kim 2023; Kim et al. 2018
Ratte, Wistar Bor:WISW (SPF-Cpb), 12 ♂ (davon 6 für BALF u. Histopathologie, 6 für Al-Quantifizierung im Gewebe), Histopathologie nur Lunge	**AlO(OH) **(γ-Modifikation, Böhmit) (Dispersal^®^, 39,4 % Al; Pural^®^, 43,9 % Al) 10 nm (Dispersal^®^), 40 nm (Pural^®^), MMAD: 10 nm: 1,7 μm (GSD 2,6), 40 nm: 0,6 μm (GSD 2,7), **28 d**, 0; 0,4; 3; 28 mg/m^3^ (10 nm: 0,16; 1,18; 11,03 mg Al/m^3^; 40 nm: 0,18; 1,32; 12,29 mg Al/m^3^), 6 h/d, 5 d/Wo, Reinheit 99,8 %, nur über die Nase, Staub, 3 Mo Nachbeobachtung, Untersuchungen nach 10 Expositionstagen und am 1., 12., 33., 91. Post-Expositionstag	**1,18 mg Al/m^3^**:** NOAEC (10 nm)**; **1,32 mg Al/m^3^**:** NOAEC (40 nm)**; **ab 1,18 (10 nm)/1,32 (40 nm) mg Al/m^3^**: Lunge: Al-Konz. korreliert positiv mit kumulativer Expositionsdosis bei 40-nm-Partikel > 10-nm-Partikel; **ab 1,32 mg Al/m^3 ^(40 nm)**: abs. Lungengew. am Ende der Nachbeobachtungszeit ↑ (ca. 7 % bei 1,32 mg Al/m^3^, 14 % bei 12,29 mg Al/m^3^); **11,03 (10 nm)/12,29 (40 nm) mg Al/m^3^**:** LOAEC**; abs. Lungengewicht ↑, abs. und rel. Gewicht hiläre Lymphknoten ↑, BALF: Gesamtprotein ↑, γ-GT ↑, Gesamtzellzahl ↑, neutrophile Granulozyten ↑, LDH ↑, β-NAG ↑, Lunge (bronchoalveoläre Region): leichte Hyperzellularität (Epithelzellen ↑, Entzündungszellen ↑, fokale septale Verdickung), fokale septale Kollagenablagerungen, Partikel in Alveolen u. Alveolarmakrophagen (einige vergrößert mit schaumigem Aussehen), Lymphknoten: deutlicher Al-Anstieg mit der Zeit bei 40 nm Partikeln; leichte lymphatische Aktivierung, Epitheloidzellenakkumulation ↑, Aluminiumkonz. im Urin ↑ (siehe Tabelle 3); Nachbeobachtung: Al-Konz. in Lunge zeitabhängig ↓, ab 28 mg/m^3^ deutlicher Anstieg der Al-Konz. in Lungen-assoziierten Lymphknoten, insbesondere bei den 40-nm-Partikeln, in Nachbeobachtungszeit Effekte in BALF nicht vollständig reversibel	Bayer Schering Pharma AG 2008; Pauluhn 2009 b
Maus, C57Bl/6, 5 ♂	„**AO nanowhiskers**“, Mischung aus Al(OH)_3_ und AlO(OH), Partikelgröße (nanowhisker): Durchmesser 2–4 nm, Länge 2800 nm (Al-Anteil 30 %), **14 d**,** 28 d**, 0; 3,3 mg/m^3 ^(0; 1 mg Al/m^3^), 4 h/d, 5 d/Wo, Ganzkörper	**1 mg Al/m^3^**: Lunge: Al-Konz. ↑, Makrophagen ↑, Gesamtzellen ↑, wird wegen spezieller Partikelgröße und Zusammensetzung, nur einer untersuchten Konz., einer möglichen zusätzlichen oralen Aufnahme (Ganzkörperexposition) nicht zur Bewertung herangezogen	Adamcakova-Dodd et al. 2012

a: Jahr; abs.: absolut; Al_2_O_3_: Aluminiumoxid; Al(OH)_3_: Aluminiumhydroxid; AlO(OH): Aluminiumoxyhydroxid; BALF: bronchoalveoläre Lavageflüssigkeit; d: Tag; Gew.: Gewicht; GRO/KC: growth-related oncogene/keratinocyte-derived chemokine; GSD: geometrische Standardabweichung; γ-GT: γ-Glutamyl-Transferase; h: Stunde; IL: Interleukin; KG: Körpergewicht; Konz.: Konzentration; k. w. A.: keine weitere Angaben; LDH: Lactatdehydrogenase; MIP-2: Macrophage inflammatory protein 2; MMAD: massenmedianer aerodynamischer Durchmesser; Mo: Monat; β-NAG: β-N-Acetylglucosaminidase; rel.: relativ; resp.: respiratorisch; TNF-α: Tumornekrosefaktor-alpha; u.: und; Wo: Woche

^a)^ Wenn nicht anders angegeben, sind die aufgeführten Veränderungen statistisch signifikant (gegen die Vehikelkontrolle).

Männliche Wistar-Ratten wurden vier Tage lang, vier Stunden am Tag, nur über die Nase gegen **Aluminiumoxid** in Konzentrationen von 0 oder 22,1 mg/m^3^ exponiert (13 nm; γ/δ-Modifikation, Suspension, MMAD: 0,38 μm). Verglichen mit der einmaligen Exposition (siehe Abschnitt 5.1.1) war nach vier Tagen die Lungenbeladung statistisch signifikant erhöht. Nach viertägiger Exposition waren in der BALF die Gesamtprotein- und LDH-Konzentrationen statistisch signifikant erhöht, was darauf hindeutet, dass die alveolokapilläre Barriere beschädigt war. Außerdem wurden eine ausgeprägte Entzündungsreaktion und ein Marker für Lipidperoxidation induziert (Details siehe Tabelle 7, Bourgois et al. 2021). Aussagen zur Konzentrations-Wirkungs-Beziehung können aus dieser Studie nicht entnommen werden, da nur eine einzige Konzentration getestet wurde. Allerdings fügen sich die beobachteten Befunde gut in die Ergebnisse der beiden im Folgenden beschriebenen 28-Tage-Studien ein.

In einer 28-Tage-Studie wurden männliche Wistar-Ratten gegen **ultrafeinen Aluminiumoxyhydroxid**-Staub in Konzentrationen von 0; 0,4; 3 oder 28 mg/m^3^ nur über die Nase, sechs Stunden pro Tag, an fünf Tagen pro Woche exponiert. Die Materialien wurden als Staub in die Expositionskammer eingebracht. Die Aluminiumkonzentration betrug 0,16; 1,18 bzw. 11,03 mg Al/m^3^ bei einer Primärpartikelgröße von 10 nm (MMAD: 1,7 μm) und 0,18; 1,32 bzw. 12,29 mg Al/m^3^ bei einer Primärpartikelgröße von 40 nm (MMAD: 0,6 μm). Die Aluminiumkonzentration in der Lunge korrelierte positiv mit der kumulativen Expositionsdosis. Die kumulative Lungenbelastung war größer nach Exposition gegen die kleineren Agglomerate (0,6 μm), welche die größere primäre Partikelgröße (40 nm) aufwiesen. In den Lungen der Tiere, die gegen die 40-nm-Partikel exponiert waren, wurde mehr Aluminium deponiert (1,85; 15,7 und 130 μg/Tag bei 0,4; 3 bzw. 28 mg/m^3^) verglichen mit den Tieren, die gegen die 10-nm-Partikel exponiert waren (1,06; 8,7 und 77,2 μg/Tag). Es traten keine substanzinduzierten Anzeichen klinischer Toxizität, Mortalität oder Effekte auf das Körpergewicht auf. In Gehirn, Nieren und Leber wurden keine signifikanten Unterschiede der Aluminiumkonzentration zwischen den Kontroll- und den exponierten Tieren beobachtet. Die Aluminiumkonzentration im Urin wurde nur bei den gegen 40-nm-Partikel exponierten Tieren untersucht. Am 25. Tag der Exposition zeigte sich ein 3,5-facher Anstieg des Aluminiumgehaltes im Urin in der höchsten Konzentrationsgruppe im Vergleich zur Kontrollgruppe (siehe Tabelle 3). Das absolute Lungengewicht sowie das absolute und relative Gewicht der Lungen-assoziierten Lymphknoten war nach der vierwöchigen Expositionszeit bei der höchsten Konzentration von 28 mg/m^3^ erhöht und in der Nachbeobachtungszeit nicht reversibel. Am Ende der Nachbeobachtungszeit war das absolute Lungengewicht der Tiere der 3-mg/m^3^-Expositionsgruppe (40 nm; 1,32 mg Al/m^3^) erhöht. In der höchsten Konzentrationsgruppe wurden nach vierwöchiger Exposition erhöhte Entzündungswerte in der BALF gemessen (siehe Tabelle 7). Nach zweiwöchiger Exposition wurden bereits Veränderungen bei den Werten für γ-Glutamyl-Transferase (GT), Protein und neutrophile Granulozyten ermittelt. In der Nachbeobachtungszeit gingen die Effekte in der BALF wieder zurück, waren aber noch nicht vollständig reversibel.

Histopathologisch wurde nur die Lunge untersucht. Bei den Tieren der höchsten Konzentrationsgruppe wurde in der bronchoalveolären Region eine leichte Hyperzellularität und fokale septale Kollagenablagerungen sowie Partikel in den Alveolen und Alveolarmakrophagen beobachtet, wobei einige vergrößert waren und ein schaumiges Aussehen aufwiesen. Die Lymphknoten zeigten eine leichte lymphatische Aktivierung, assoziiert mit einer erhöhten Epitheloidzellenakkumulation. Im Bulbus olfactorius einschließlich der ethmoidalen Nasengänge wurden keine Partikel und keine spezifischen lokalen Veränderungen gesehen. In Gehirn, Nieren und Leber zeigten sich keine signifikanten Unterschiede der Aluminium-Konzentration zwischen Kontroll- und exponierten Tieren. Die LOAEC beträgt nach den Autoren 28 mg/m^3^ (11,03/12,29 mg Al/m^3^) aufgrund der Entzündungsreaktionen in der Lunge und der verlangsamten Clearance (Bayer Schering Pharma AG 2008, 2009; Pauluhn 2009 b). Die Kommission sieht das um ca. 7 % erhöhte absolute Lungengewicht bei 3 mg/m^3^ (40-nm-Gruppe; 1,32 mg Al/m^3^) nicht als adversen expositionsbedingten Effekt an. Das Lungengewicht nimmt mit dem Alter zu. Da Alter und Körpergewicht bei der Bewertung des Lungengewichts eine Rolle spielen, ist nur das relative Lungengewicht aussagekräftig. Das relative Lungengewicht war in der 3-mg/m^3^-Gruppe nicht verändert und Entzündungszeichen traten nicht auf. Die Kommission sieht somit die NOAEC dieser Studie bei 3 mg/m^3^ (1,18 /1,32 mg Al/m^3^).

In dieser Studie zeigt sich ein Überladungseffekt der Lunge (Abschnitt 2.1) sowie die Bioverfügbarkeit des ultrafeinen Aluminiumoxyhydroxids. Es ist davon auszugehen, dass der Anstieg von Aluminium im Urin bei der höchsten Konzentration gegen Ende der Expositionszeit (ab dem 11. Tag und sehr deutlich am 25 Tag, siehe Tabelle 3) aus einem verzögerten lysosomalen Abbau durch Makrophagen in der Lunge resultiert.

In einer zweiten 28-Tage-Studie wurden männliche Sprague-Dawley-Ratten fünf Tage pro Woche, an sechs Stunden pro Tag, nur über die Nase gegen **ultrafeine Aluminiumoxid**partikel (γ-Modifikation (Kim 2023)) in Konzentrationen von 0; 0,2; 1 oder 5 mg/m^3^ (0; 0,1; 0,5; 2,6 mg Al/m^3^) exponiert. Eine wässrige Suspension des Pulvers wurde durch Sonifizierung dispergiert und dann in die Expositionskammer versprüht. Die Aluminiumgehalte waren im Lungengewebe konzentrationsabhängig erhöht. Die Akkumulation von Alveolarmakrophagen in der Lunge der Ratten der 5-mg/m^3^-Gruppe nahm tendenziell zum Ende der Nachbeobachtungsphase zu. Bei dieser Konzentration waren das absolute und relative Lungengewicht am Expositionsende statistisch signifikant erhöht. In der BALF-Analyse nach 28-tägiger Exposition zeigte sich ein Anstieg der polymorphkernige Leukozyten schon ab 0,2 mg/m^3^ (Details siehe Tabelle 7). Marker für oxidativen Stress (Katalase, GSH und Thiobarbitursäure-reaktive Substanz (TBARS)) waren nicht verändert. Weiterhin ließen sich keine Effekte auf das Körpergewicht, keine histopathologischen Veränderungen an Leber, Nieren, Milz und Gehirn sowie keine veränderten Blutparameter (Alanin-Aminotransferase (ALT), Aspartat-Aminotransaminase (AST), LDH, weiße Blutzellen (WBC), Lymphozyten, Eosinophile) bis zur höchsten untersuchten Konzentration feststellen. Die NOAEC beträgt 1 mg/m^3^ (0,5 mg Al/m^3^) aufgrund der Befunde in der Lunge bei 5 mg/m^3^ (2,6 mg Al/m^3^) (Kim et al. 2018). Unstimmigkeiten zwischen Text und Tabellen wurden bei den Autoren angefragt und geklärt (Abbildung 3, Bild F zeigt die Effekte bei 1 mg/m^3^ anstelle von 0,2 mg/m^3^; Tabelle 9 bei 1 mg/m^3^ “inflammatory cells infiltration” 2 anstelle von 0 (Kim 2023)).

Nicht geklärt werden konnte die unterschiedliche Lungenbeladung in der Studie von Kim et al. (2018) verglichen mit der Studie von Pauluhn (Bayer Schering Pharma AG 2008, 2009; Pauluhn 2009 b) (siehe Tabelle 8). Möglicherweise sind bei Kim et al. (2018) mg Al/g Lunge statt μg Al/g Lunge gemeint. Bei 10-fach niedrigerer Exposition liegt bei Kim et al. (2018) eine fast 1000-fach niedrigere Aluminium-Beladung in der Lunge vor verglichen mit den Daten von Pauluhn (Bayer Schering Pharma AG 2008, 2009; Pauluhn 2009 b), andererseits beginnen die Effekte in der Studie von Kim et al. (2018) schon etwa bei einer um den Faktor 20 niedrigeren Konzentration in der Luft.

Bei dem Anstieg von Neutrophilen (gleichbedeutend mit neutrophilen Granulozyten) in der BALF handelt es sich um einen sensitiven Entzündungsmarker, welcher bereits vor histopathologischen Befunden auftritt. Beim gesunden Menschen ist der Anteil an Neutrophilen 0–4 % (Olsen et al. 2012). Es ist davon auszugehen, dass der Anteil an Neutrophilen bei der Ratte ähnlich ist. Ein Anstieg auf 6,6 % bei 0,2 mg/m^3^ (0,1 mg Al/m^3^) ist daher ein deutliches Zeichen für einen beginnenden Entzündungseffekt.

**Tab. 8:** Vergleich Lungenbeladung der beiden bewertungsrelevanten 4-Wochenstudien (Bayer Schering Pharma AG 2008, 2009; Kim 2023; Kim et al. 2018; Pauluhn 2009 a, b)

**Parameter**	**Pauluhn**		**Kim**
**Substanz**	**AlO(OH)**	**AlO(OH)**	**Al_2_O_3_**
Primärpartikelgröße [nm]	10	40	12
MMAD [μm]	1,7	0,6	0,38–0,5
NOAEC [mg Al/m^3^]	1,18	1,32	0,1
Lungenbeladung [µg Al/g Lunge]	ca. 100^a)^	ca. 170^a)^	0,22
LOAEC [mg Al/m^3^]	11,03	12,29	0,5
Lungenbeladung [µg Al/g Lunge]	ca. 800^a)^	ca. 1300^a)^	1,298
Weitere Konzentrtion [mg Al/m^3^]	–	–	2,5
Lungenbeladung [µg Al/g Lunge]	–	–	5,038

^a)^ Werte anhand Abbildung abgeschätzt

**Fazit Lungentoxizität: **Es liegen zwei bewertungsrelevante 28-Tage-Inhalationstudien vor (Bayer Schering Pharma AG 2008, 2009; Kim et al. 2018; Pauluhn 2009 b). Aufgrund der Unklarheiten zur Angabe der Lungenbeladung bei Kim et al. (2018) und der im Vergleich detaillierter beschriebenen Untersuchung von Pauluhn (Bayer Schering Pharma AG 2008, 2009; Pauluhn 2009 b) wird letztere für die Ableitung des MAK-Wertes herangezogen. In dieser 28-Tage-Inhalationsstudie an männlichen Wistar-Ratten mit Exposition gegen **Aluminiumoxyhydroxid**-Staub in Konzentrationen von 0; 0,4; 3 oder 28 mg/m^3^ (0,16/0,18; 1,18/1,32 bzw. 11,03/12,29 mg Al/m^3^; 10 oder 40 nm, MMAD: 1,7 μm bzw. 0,6 μm) zeigten sich in den höchsten Konzentrationsgruppen erhöhte Entzündungswerte in der BALF. Nach zweiwöchiger Exposition waren bereits γ-GT, Protein und neutrophile Granulozyten erhöht. Ebenfalls nur bei der höchsten Konzentration waren das absolute Lungengewicht sowie das absolute und relative Gewicht der Lungen-assoziierten Lymphknoten erhöht. In der bronchoalveolären Region wurden eine leichte Hyperzellularität und fokale septale Kollagenablagerungen nachgewiesen. Die NOAEC der Untersuchung beträgt 1,18 mg Al/m^3 ^bzw. 1,32 mg Al/m^3^ (Bayer Schering Pharma AG 2008, 2009; Pauluhn 2009 b). Andere Organe außer der Lunge wurden nicht histopathologisch untersucht.

In der 28-Tage-Inhalationsstudie von Kim et al. (2018) zeigten sich keine histopathologischen Effekte an Leber, Nieren, Milz und Gehirn bis zur höchsten Konzentration von 2,6 mg Al/m^3^. Somit ist anzunehmen, dass bei der NOAEC von 1,18 /1,32 mg Al/m^3^ (Bayer Schering Pharma AG 2008, 2009; Pauluhn 2009 b) auch keine systemischen Effekte aufgetreten sind.

#### 5.2.2 Orale Aufnahme 

Studien zur Toxizität nach wiederholter oraler Gabe von schwerlöslichen Aluminiumverbindungen sind im Nachtrag 2007 (Greim 2007) nicht enthalten. Relevante Veröffentlichungen werden in Tabelle 9 aufgeführt. In den Studien, in denen jeweils nur eine Dosis untersucht wurde (Abdelhameed et al. 2023; Abo-EL-Sooud et al. 2023; Abou-Zeid et al. 2021; Atia und Alghriany 2021; Cao et al. 2018, 2020; Naji et al. 2023; Nannepaga et al. 2014; Singla und Dhawan 2013, 2014; de Souza et al. 2023; Wasana et al. 2015; Yousef et al. 2019; Zhang et al. 2021, 2022 a), kann keine Dosis-Wirkungs-Beziehung aufgezeigt werden. Zudem sind die in diesen Studien eingesetzten Aluminiumdosen höher als jene von Park et al. (2015). Daher werden diese Studien hier nicht im Detail dargestellt. Außerdem werden einige Untersuchungen nicht zur Bewertung herangezogen, da hohe Aluminiumdosierungen (> 250 mg Al/kg KG) verwendet wurden (Hicks et al. 1987; Park et al. 2022; Thorne et al. 1986).

**Tab. 9:** Studien mit wiederholter oraler Exposition gegen schwerlösliche Aluminiumverbindungen

**Spezies, ** **Stamm, ** **Anzahl pro Gruppe **	**Exposition**	**Befunde** ^a), c)^	**Literatur**
Maus, Swiss Albino, 5 ♂	**Al_2_O_3_** (vermutlich γ-Modifikation, laut Herstellerkatalog), (Partikelgröße < 50 nm), **5 d**, 0, 15, 30, 60 mg/kg KG u. d (0, ca. 8, 16, 32 mg Al/kg KG u. d), Gavage, Reinheit (k. A.)	**ab 8 mg Al/kg KG: **Hoden: CAT ↓; **bei 16 mg Al/kg KG: **ALP ↑, veränderte Spermienkopf-Morphologie, Gehirn u. Hoden: LPO ↑; **ab 16 mg Al/kg KG: **AST ↑, ALT ↑, Gehirn: neurofibrilläre Bündel, SOD ↓, GSH ↓, Leber: erweiterte Zentralvene u. Portaltrakt, LPO ↑, CAT ↑, SOD ↓, GSH ↓, Nieren: LPO ↑, CAT ↑, SOD ↓, GSH ↓, Hoden: SOD ↓; **32 mg Al/kg KG: **Lymphozyten ↑, Hoden: GSH ↓	De et al. 2020
Maus, ICR, 12 ♂ (davon 4 Tiere/Gruppe für Histopathologie Leber, Nieren)	**Al_2_O_3_** (γ-Modifikation), (Partikelgröße < 50 nm), **13 Wo**, 0; 1,5; 3; 6 mg/kg KG u. d (0; ca. 0,8; 1,6; 3,2 mg Al/kg KG u. d), Trinkwasser, 6 d/Wo, Reinheit 99,8 %	**0,8 mg Al/kg KG: LOAEC**; **ab 0,8 mg Al/kg KG:** Wasser/Futter-Konsum^b)^ ↓ (0,8; 1,6; 3,2 mg Al/kg KG: Wasserkonsum 17 % ↓, 10 % ↓, 16 % ↓; Futterkonsum: 8 % ↓, 3 % ↓, 13 % ↓), Blut: IL-6 ↑; **bei 0,8 mg Al/kg KG:** BUN ↓, Nieren: minimale bis leichte Tubulus-Vakuolisierung (0,8; 1,6; 3,2 mg Al/kg KG: 1/4, 0/4, 2/4); **ab 1,6 mg Al/kg KG:** Blut: Lymphozyten ↑, MCP-1 ↑, LDH ↓, Leber: leichte Akkumulationen von Partikeln (1/4), Hypertrophie (1/4); **bei 1,6 mg Al/kg KG: **IL-1 ↑; **3,2 mg Al/kg KG:** KG-Zunahme ↓, Blut: AST ↑, ALT ↑ , LDH ↑ , Leukozyten ↑, Eosinophile ↓, TFG-β ↑, Leber: minimale Akkumulationen von Partikeln (1/4), mäßige chronische Entzündung (1/4), leichte Nekrose (1/4)	Park et al. 2015

Al: Aluminium; Al_2_O_3_: Aluminiumoxid; ALP: alkalische Phosphatase; ALT: Alanin-Aminotransferase; AST: Aspartat-Aminotransferase; BUN: Blut-Harnstoff-Stickstoff; CAT: Katalase; d: Tag; GSH: Glutathion; IL: Interleukin; LDH: Lactatdehydrogenase; LPO: Lipidperoxidation; MCP: Monozyten-chemotaktisches Protein; SOD: Superoxiddismutase; TFG: transforming growth factor; Wo: Woche

^a)^ Wenn nicht anders angegeben, sind die aufgeführten Veränderungen statistisch signifikant (gegen die Vehikelkontrolle).

^b)^ Wasser/Futterkonsum wurde alle 7 Tage untersucht, Effekt zeigte sich in der letzten Untersuchungswoche im Vergleich zur Kontrollgruppe

^c)^ Bei den Enzymen wurde die Aktivität untersucht.

Von den beiden bewertungsrelevanten Studien (De et al. 2020; Park et al. 2015) zeigen sich bei der Untersuchung von Park et al. (2015) Effekte bei niedrigeren Aluminiumdosen. In dieser Trinkwasserstudie wurden je zwölf männliche ICR-Mäuse pro Gruppe gegen **ultrafeines Aluminiumoxid** (γ-Modifikation) in Dosierungen von 0; 1,5; 3; 6 mg/kg KG und Tag (0 ca. 0,8; 1,6; 3,2 mg Al/kg KG und Tag) exponiert. Ab 0,8 mg Al/kg KG und Tag ist eine Verminderung des Wasser- und Futterkonsums zu beobachten, was nicht bewertet werden kann, da die Tiere schlecht randomisiert waren. Die Kontrolltiere hatten schon zu Beginn der Studie ein höheres Körpergewicht im Vergleich zu den mit Aluminiumoxid dosierten Tieren. Weiterhin zeigten sich ab der niedrigsten Konzentration eine Zunahme von IL-6 im Blut sowie minimale bis leichte Veränderungen in den Nieren. Ab 1,6 mg Al/kg KG und Tag ließen sich dosisabhängig histopathologische Veränderungen der Leber sowie eine Zunahme von Entzündungsmarkern und Lymphozyten im Blut erkennen. Bei 3,2 mg Al/kg KG und Tag waren zusätzlich die Körpergewichtszunahme reduziert und verschiedene Blutparameter verändert.

Die Studien mit wiederholter oraler Gabe von schwerlöslichen Aluminiumverbindungen spiegeln nicht die Verteilung nach Inhalation wider, bei der außer in der Lunge keine signifikante Aluminiumbelastung in Leber, Nieren und Gehirn beobachtet wurde (siehe Abschnitt 3.1.6). Sie sind daher nicht für eine Bewertung der Toxizität unter Arbeitsplatzbedingungen geeignet.

### 5.3 Wirkung auf Haut und Schleimhäute 

#### 5.3.1 Haut

**Aluminiumhydroxid** und **Aluminiumoxid** wirken nicht hautreizend (Tabelle 10).

**Tab. 10:** Studien zur Reizwirkung an der Haut durch schwerlösliche Aluminiumverbindungen

**Anzahl**	**Dauer**	**Verbindung, Dosis**	**Effekte**	**Bewertung**	**Literatur**
**Neuseeländer-Kaninchen**					
3 ♂	4 h, semiokklusiv, Ablesezeitpunkte: 1, 24, 48, 72 h, OECD-Prüfrichtlinie 404 (allerdings Testsubstanz nicht angeteigt)	**Al(OH)_3_**, 0,5 g (ohne Lösemittel)	1 h: sehr leichte Erytheme (bei 2/3 Tieren), 24, 48, 72 h: keine Effekte	nicht hautreizend	LAB Research Ltd. 2009 c
6 k. w. A.	24 h, okklusiv, intakte und abradierte Haut (je 3), Ablesezeitpunkte: 24, 72 h	**Al_2_O_3_** (k. A. zur Kristallform), 0,5 g (Pulver, mit 1 ml H_2_O)	intakte Haut: keine Effekte, abradierte Haut: leichte Erytheme nach 24 h, reversibel nach 72 h	nicht hautreizend	ECHA 2023 c
12 k. w. A.	24 h, semiokklusiv, intakte und abradierte Haut (je 6), Ablesezeitpunkte: 24, 72 h	**Al_2_O_3_** (k. A. zur Kristallform), 0,5 g (ohne Lösemittel)	intakte Haut: leichte Erytheme bei 2/6 Tieren nach 24 h, reversibel nach 72 h, abradierte Haut: kein Effekt	nicht hautreizend	ECHA 2023 c
12 k. w. A.	24 h, semiokklusiv, intakte und abradierte Haut (je 6), Ablesezeitpunkte: 24, 72 h	**Al_2_O_3_** (k. A. zur Kristallform), 0,5 g (k. w. A.)	intakte Haut: leichte Erytheme bei 1/6 Tieren nach 24 h sowie bei einem Tier nach 72 h abradierte Haut: kein Effekt	nicht hautreizend	Degussa Frankfurt 1979 b
3 k. w. A.	5 d, offen, rasierte Haut, Ablesezeitpunkt: 24 h	**Al(OH)_3_**, 10 % (G/V), Suspension mit 0,2 % Tween-80 pH 7,2	k. A.	nicht hautreizend	ECHA 2023 b
**Albino-Maus**					
5 k. w. A.	5 d, offen, rasierte Haut, Ablesezeitpunkt: 24 h	**Al(OH)_3_**, 10 % (G/V), Suspension mit 0,2 % Tween-80 pH 7,2	k. A.	nicht hautreizend	ECHA 2023 b
**Schwein**					
2 k. w. A.	5 d, offen, rasierte Haut, Ablesezeitpunkt: 24 h	**Al(OH)_3_**, 10 % (G/V), Suspension mit 0,2 % Tween-80 pH 7,2	k. A.	nicht hautreizend	ECHA 2023 b
**In-vitro-Studien**					
	Humanhaut, LabCyte EPI-Model24 SIT, 3 Replikate, Exposition: 15 min, Ablesezeitpunkt: 42 h, OECD TG 439	**Al_2_O_3_** (k. A. zur Kristallform), 0,025 g in 0,5 ml H_2_O	OD: 0,953 Gewebelebensfähigkeit: 97,9 ± 3,9 %	nicht hautreizend	ECHA 2023 c

Al_2_O_3_: Aluminiumoxid; Al(OH)_3_: Aluminiumhydroxid; d: Tag; h: Stunde; k. (w.) A.: keine (weitere) Angabe; OD: optische Dichte; TG: Prüfrichtlinie (Test Guideline)

#### 5.3.2 Auge

**Aluminiumhydroxid** und **Aluminiumoxid **wirken nicht reizend am Kaninchenauge (Tabelle 11).

**Tab. 11:** Studien zur Reizwirkung am Auge von Neuseeländer-Kaninchen

**Anzahl, Dauer**	**Verbindung, Dosis**	**Effekte (Grad nach Draize^a)^)**	**Bewertung**	**Literatur**
3 ♂, Ablesezeitpunkte: 1, 24, 48, 72 h, OECD-Prüfrichtlinie 405	**Al(OH)_3_**, 100 mg, gespült nach 1 h	**1 h**: gespült: Rötungen Grad 2 (3/3), Absonderung der Bindehäute, Konjunktivitis Grad 2 (1/3), Absonderung der Bindehäute, Konjunktivitis Grad 1 (2/3); **24 h**: leichte Rötung (2/3); **48 h**: keine Effekte	nicht augenreizend	LAB Research Ltd. 2009 b
9 (k. w. A.), Ablesezeitpunkte: 24, 48, 72 h, 4, 7 d	**Al_2_O_3_** (γ-Modifikation), 100 mg, nicht gespült: 3 Tiere, gespült nach 2 sec: 3 Tiere, gespült nach 4 sec: 3 Tiere	**24 h**: nicht gespült: Bindehautrötung Grad 1 (3/3), gespült (2 sec): Bindehautrötung Grad 1 (2/3), gespült (4 sec): keine Effekte; **48 h**: keine Effekte	nicht augenreizend	ECHA 2023 c
6 (k. w. A.), Ablesezeitpunkte: 24, 48, 72 h, 7 d, nach FDA (Fed. Reg. 28 (119), 5582, 1963)	**Al_2_O_3_** (k. w. A.), 100 mg, nicht gespült	**24 h**: Bindehautrötung Grad 1 (6/6), Bindehautschwellung Grad 1 (6/6); **48 h**: Bindehautrötung Grad 1 (6/6), Bindehautschwellung Grad 1 (4/6); **72 h**: Bindehautrötung Grad 1 (6/6); **7 d**: Bindehautrötung Grad 1 (1/6)	nicht augenreizend	Degussa Frankfurt 1979 b
6 (k. w. A.), Ablesezeitpunkte: 24, 48, 72 h, 7 d, nach FDA (Fed. Reg. 28 (119), 5582, 1963)	**Al_2_O_3_** (k. w. A.), 100 mg, nicht gespült	**24 h**: Bindehautrötung Grad 1 (5/6), 2 (1/6), Bindehautschwellung Grad 1 (3/6); **48 h**: Bindehautrötung Grad 1 (6/6), Bindehautschwellung Grad 1 (2/6); **72 h**: Bindehautrötung Grad 1 (6/6); **7 d**: Bindehautrötung Grad 1 (2/6)	nicht augenreizend	ECHA 2023 c

^a)^ Bindehautrötung max. Grad: 3; Bindehautschwellung max. Grad 4

Al_2_O_3_: Aluminiumoxid; Al(OH)_3_: Aluminiumhydroxid; d: Tage; h: Stunden; k. w. A.: keine weiteren Angaben; TG: Prüfrichtlinie (Test Guideline)

Auch in einer In-vitro-Studie (EpiOcular^TM^ EIT) nach OECD-Prüfrichtlinie 492 war **Aluminiumoxid** nicht augenreizend (ECHA 2023 c).

### 5.4 Allergene Wirkung 

#### 5.4.1 Hautsensibilisierende Wirkung

Im letzten Nachtrag (Greim 2007) war bereits eine Untersuchung am Meerschweinchen mit **Aluminiumhydroxid** mit negativem Ergebnis aufgeführt.

Ein Landsteiner-Draize-Test mit zwei **Aluminiumoxid**-Verbindungen unbekannter Kristallmodifikation (Aluminiumoxid „TBH“ 43/79 und Aluminiumoxid „TOF” 44/79) an Albino-SPF-Meerschweinchen verlief negativ. Es erfolgten zehn intradermale Injektionen mit einer 33%igen wässrigen Testzubereitung, dreimal wöchentlich in drei Wochen. Die Testsubstanzen bewirkten leichte bis mäßige Hautreaktionen während der Induktionsperiode. Auf die Provokation mit der ebenfalls 33%igen Testzubereitung des TBH-Aluminiumoxids wurden schwache Reaktionen bei allen zehn Tieren beobachtet, wobei auch bei sieben Kontrolltieren eine leichte und bei einem Tier eine mäßige Reaktion beobachtet wurde. Die Provokation mit dem „TOF“-Aluminiumoxid bewirkte sechs schwache und zwei mäßige Reaktionen bei den zehn behandelten Tieren, während bei den Kontrolltieren sieben schwache und eine mäßige Reaktion beobachtet wurde. Da es keine Unterschiede zwischen behandelten und Kontrolltieren gab, wurde das Testergebnis als negativ bewertet (Degussa Frankfurt 1979 c.)

Ein Maximierungstest mit **Aluminiumhydroxid** nach OECD-Prüfrichtlinie 406 an zehn Dunkin-Hartley-Meerschweinchen verlief negativ. Die intradermale Induktion erfolgte mit einer 1%igen Testzubereitung in 1%iger wässriger Methylcellulose-Lösung, die epikutane Induktion mit der 100%igen (G/V) Testzubereitung in Methylcellulose-Lösung. Auf die Provokation mit der 75%igen und 37,5%igen Testzubereitung in Methylcellulose-Lösung reagierte keines der zehn Tiere (LAB Research Ltd. 2010). Um das negative Testergebnis zu bestätigen, wäre die Testung von weiteren zehn Tieren erforderlich gewesen.

Es liegen keine Untersuchungen mit tierversuchsfreien Alternativverfahren vor.

#### 5.4.2 Atemwegssensibilisierende Wirkung

Zur Untersuchung der atemwegssensibilisierenden Wirkung am Tier steht derzeit kein validiertes Modell zur Verfügung.

Je 16 ICR-Mäuse pro Gruppe erhielten vier intratracheale Instillationen von Kochsalzlösung oder 0,1 mg **Aluminiumoxid** (Partikelgröße 1–5 µm) in Kochsalzlösung im Abstand von zwei Wochen. Die Tiere wurden einen Tag nach der letzten Instillation getötet. Acht von 16 Tieren in jeder Gruppe wurden für die histopathologische Untersuchung verwendet. Aluminiumoxid erhöhte nicht die Anzahl von Eosinophilen und Lymphozyten in der Submukosa, die Proliferation der Becherzellen in den Atemwegen, die Menge an Interleukinen, die Anzahl der Zellen in der BALF sowie den IgE-Spiegel im Serum (Ichinose et al. 2008).

### 5.5 Reproduktionstoxizität 

Daten zur Reproduktionstoxizität von Aluminiumverbindungen wurden in Übersichtsartikeln aufgeführt (ATSDR 2008; EFSA 2008; Yokel 2020).

Zu **metallischem Aluminium**, **Aluminiumoxid** und **Aluminiumoxyhydroxid** liegen keine Studien zur Reproduktionstoxizität vor.

#### 5.5.1 Fertilität

In Greim (2007) wurden keine Studien zur Fertilität nach Verabreichung von Aluminium und schwerlöslichen Aluminiumverbindungen aufgeführt. Seit Erscheinen dieses Nachtrags sind auch keine Studien publiziert worden.

In einer Studie an Sprague-Dawley-Ratten mit 28-tägiger Exposition gegen **ultrafeine Aluminiumoxid**partikel (γ-Modifikation) ergab die makroskopische Untersuchung der Reproduktionsorgane bis 2,6 mg Al/m^3^ keine auffälligen Befunde (Kim et al. 2018) (siehe Abschnitt 5.2.1.2). Nach Inhalation ist für Aluminium und seine schwerlöslichen Verbindungen der kritische Endpunkt die Wirkung auf die Lunge und Effekte auf Fertilität und Reproduktionsorgane sind aufgrund der geringen Bioverfügbarkeit nicht als kritisch anzusehen.

Studien mit **Aluminiumhydroxid **als Adjuvans in Impfstoffen werden aufgrund der fehlenden Arbeitsplatzrelevanz nicht berücksichtigt.

#### 5.5.2 Entwicklungstoxizität

Es liegen keine Studien zur Entwicklungstoxizität nach inhalativer Exposition gegen Aluminium oder seinen schwerlöslichen Verbindungen vor.

Nach oraler Verabreichung haben sich NOAEL für pränatale Entwicklungstoxizität und Maternaltoxizität bei Ratten von jeweils 768 mg **Aluminiumhydroxid**/kg KG und Tag (266 mg Al/kg KG und Tag) (Gomez et al. 1990, 1991; Greim 2007) und bei Mäusen von 266 bzw. 300 mg Aluminiumhydroxid/kg KG und Tag (92 bzw. 103 mg Al/kg KG und Tag) (Colomina et al. 1992, 1994; Domingo et al. 1989; Greim 2007), den höchsten eingesetzten Dosierungen, ergeben. Diese Studien sind nicht konform zur OECD-Prüfrichtlinie 414 durchgeführt worden. Die Ergebnisdarstellung ist eingeschränkt. Der Untersuchungsumfang der Muttertiere und Feten hinsichtlich externer, viszeraler und skelettaler Veränderungen ist ausreichend. Auch die Auswertung wurde OECD-Prüfrichtlinien-konform auf Wurfbasis vorgenommen. In der Studie von Colomina et al. (1994) fehlt die Angabe zur Tierzahl. In der Summe sind die Studien als valide anzusehen und decken den Endpunkt pränatale Entwicklungstoxizität für Aluminiumhydroxid nach oraler Gabe ab. Messungen der Aluminiumkonzentration bei Muttertieren und Feten zeigen die geringe Bioverfügbarkeit des schwerlöslichen Aluminiumhydroxids (siehe Abschnitt 3.1.5).

Seit Erscheinen des Nachtrags von 2007 (Greim 2007) ist eine Studie durchgeführt worden. Je zwölf Sprague-Dawley-Ratten erhielten vom 7. bis zum 16. Gestationstag 0, 100 oder 200 mg **ultrafeine** (32 nm) **Aluminiumoxid**-Partikel/kg KG und Tag (0, 20, 40 mg Al/kg KG und Tag) bzw. 200 mg **mikroskalige Aluminiumoxid**-Partikel/kg KG und Tag (Positivkontrolle: 300 mg Acetylsalicylsäure/kg KG und Tag) per Schlundsonde (Vehikel: entionisiertes Wasser). Bis zur höchsten Dosis traten keine auffälligen Befunde bei Muttertieren (Mortalität, Symptome, Körpergewicht, Futterverbrauch, Anzahl der Corpora lutea, Gewicht von Ovarien und Uterus, Anzahl der Implantationen und Resorptionen, Anzahl lebender Feten) sowie Feten (Körpergewicht, Geschlechterverhältnis, Körper- und Schwanzlänge, externe, skelettale und viszerale Fehlbildungen/Variationen) auf (Gao et al. 2022). Die Studie wurde nicht nach gültigen Prüfrichtlinien durchgeführt. Jedoch war die Methodik zur Untersuchung der Feten auf skelettale und viszerale Veränderungen Prüfrichtlinien-konform. Es erfolgte keine Auswertung auf Wurfbasis, nur auf Fetenbasis.

Studien zur Entwicklungsneurotoxizität sind mit Aluminium und seinen schwerlöslichen Verbindungen nicht durchgeführt worden.

### 5.6 Genotoxizität 

Daten zur Genotoxizität sind umfassend im vorherigen Nachtrag dargestellt (Greim 2007). Ergänzt werden in diesem Nachtrag neue Daten zu ultrafeinem, elementarem Aluminium sowie zu mikroskaligen und ultrafeinen schwerlöslichen Aluminiumverbindungen. In einigen Untersuchungen lässt sich nicht zuordnen, ob das unlösliche **Korund** oder **schwerlösliches Aluminiumoxid** anderer Modifikation (z. B. delta, gamma) eingesetzt wurden. Diese Studien wurden mitaufgeführt, sind aber aufgrund ihrer mangelnden Materialcharakterisierung für die Bewertung des Endpunktes nicht heranziehbar.

#### 5.6.1 In vitro

Neue Daten zu elementarem Aluminium und schwerlöslichen Aluminiumverbindungen in In-vitro-Untersuchungen sind in Tabelle 12 dargestellt.

**Tab. 12:** In-vitro-Studien zur Genotoxizität mit ultrafeinem, elementarem Aluminium sowie mikroskaligen und ultrafeinen schwerlöslichen Aluminiumverbindungen

**Endpunkt**	**Testsubstanz ** **Testsystem, Zeit**	**Konzentration **	**wirksame Konz.**	**Zytotoxizität/Anmerkung**	**Ergebnis**		**Literatur**
					**–m. A.**	**+m. A.**	
**Ultrafeines, elementares Aluminium**							
DNA-Schäden (γ-H2AX)	**Al^0^** (18 nm), suspendiert in Wasser mit 0,05 % BSA, Caco-2-Zellen, HepaRG-Zellen, 24 h Inkubation	0, 3, 9, 28, 40, 80 µg/cm^2^ (0, 9, 28, 90, 128, 256 µg Al/ml)	HepaRG: 256 µg Al/ml Caco-2: –	Zytotoxizität (Zellzahl, aktive Caspase-3): –	–	n. u.	Jalili et al. 2022
DNA-Schäden (Comet-Assay, alkalisch)	**Al^0^** (18 nm), suspendiert in Wasser mit 0,05 % BSA, Caco-2-Zellen, HepaRG-Zellen, 5 u. 24 h Inkubation	0, 3, 9, 28, 40, 80 µg/cm^2^ (0, 9, 28, 90, 128, 256 µg Al/ml)	5 h: Caco-2: ab 90 µg Al/ml; HepaRG: ab 28 µg Al/ml; 24 h: ab 90 µg Al/ml in beiden Zelllinien	Zytotoxizität (Zellzahl, aktive Caspase-3): – (nur nach 24 h untersucht)	+	n. u.	Jalili et al. 2022
Oxidative DNA-Schäden (Comet-Assay mit FPG, alkalisch)	**Al^0^** (18 nm), suspendiert in Wasser mit 0,05 % BSA, Caco-2-Zellen, HepaRG-Zellen, 5 u. 24 h Inkubation	0, 3, 9, 28, 40, 80 µg/cm^2^ (0, 9, 28, 90, 128, 256 µg Al/ml)	5 h: Caco-2: ab 28 µg Al/ml; HepaRG: nur „hedgehogs“ beobachtet; 24 h: HepaRG: ab 9 µg Al/ml	Zytotoxizität (Zellzahl, aktive Caspase-3): – (nur nach 24 h untersucht)	+	n. u.	Jalili et al. 2022
MN	**Al^0^** (18 nm), suspendiert in Wasser mit 0,05 % BSA, Caco-2-Zellen, HepaRG-Zellen, 24 h Inkubation	0, 3, 9, 28, 40, 80 µg/cm^2^ (0, 9, 28, 90, 128, 256 µg Al/ml)	–	Zytotoxizität (RI: replicative index): –	–	n. u.	Jalili et al. 2022
**Schwerlösliche mikroskalige Aluminiumverbindungen**							
Genmutation (Präinkubation)	**Al_2_O_3_** (50–100 µm), keine Angabe zur Modifikation, suspendiert in DMSO/Wasser (1:1), S. typhimurium TA97a, TA98, TA100, TA102, TA1535	0, 20, 40, 75, 150, 300, 600, 1250, 2500 µg/Platte (0, 11, 21, 40, 80, 159, 318, 663, 1325 µg Al/Platte)	–	–	–	–	Balasubramanyam et al. 2010
Genmutation (Platteninkorporation)	**Al_2_O_3_** (10 µm), k. A. zur Modifikation, suspendiert in Wasser, S. typhimurium TA97, TA98, TA100, TA102	0; 0,5; 5; 50; 500; 5000 µg/Platte (0; 0,3; 3; 27; 265; 2650 µg Al/Platte)	–	–	–	–	Zhang et al. 2017
DNA-Schäden (Comet-Assay, alkalisch)	**Al_2_O_3_** (10 µm), k. A. zur Modifikation, suspendiert in Wasser, CHL-Zellen, 12, 24, 48 h Inkubation	0, 15, 30, 60 µg/ml (0, 8, 16, 32 µg Al/ml)	12 und 24 h: 32 µg Al/ml, 48 h: ab 16 µg Al/ml	Zytotoxizität (Trypanblau): 6 µg Al/ml: 18 %, 13 µg Al/ml: 26 %, 25 µg Al/ml: 35 %, 50 µg Al/ml: 41 %, 100 µg Al/ml: 61 % (nur nach 24 h bestimmt); Untersuchungen zu Markern für oxidativen Stress siehe Abschnitt 2.3.	+	n. u.	Zhang et al. 2017
Genmutation Tk^+/–^	**Al(OH)_3_**, suspendiert in Wasser mit 0,5 % Methylcellulose, Mauslymphomzellen L5178Y, 3 h Inkubation, OECD TG 476	0, 6, 12, 24, 49, 98, 195, 390, 780 µg/ml (0, 2, 4, 8, 17, 33, 66, 133, 265 µg Al/ml)	–	Zytotoxizität: „relative total growth“ (RTG) bei 780 µg/ml: Exp1: 92 % (–m. A.), 66 % (+m. A.), Exp2: 101 % (–m. A.), 93 % (+m. A.)	–	–	Covance Laboratories Ltd 2010 b
**Schwerlösliche ultrafeine Aluminiumverbindungen **							
Genmutation (Präinkubation)	**Al_2_O_3_** (30, 40 nm), k. A. zur Modifikation, suspendiert in DMSO/Wasser (1:1), S. typhimurium TA97a, TA98, TA100, TA102, TA1535	0, 20, 40, 75, 150, 300, 600, 1250, 2500 µg/Platte (0, 11, 21, 40, 80, 159, 318, 663, 1325 µg Al/Platte)	–	–	–	–	Balasubramanyam et al. 2010
Genmutation (Präinkubation)	**Al_2_O_3_** (< 50 nm), k. A. zur Modifikation, suspendiert in Wasser, S. typhimurium TA97a, TA100, Escherichia coli WP2 trp uvrA	0, 10, 100, 1000 µg/Platte (0, 5, 53, 530 µg Al/Platte)	–	–	–	–	Pan et al. 2010
Genmutation (Platteninkorporation)	**Al_2_O_3_** (13, 50 nm), γ-Modifikation, suspendiert in Wasser, S. typhimurium TA97, TA98, TA100, TA102	0; 0,5; 5; 50; 500; 5000 µg/Platte (0; 0,3; 3; 27; 265; 2650 µg Al/Platte)	–	–	–	–	Zhang et al. 2017
DNA-Schäden (γ-H2AX)	**Al_2_O_3_** (20 nm), γ-Modifikation, suspendiert in Wasser mit 0,05 % BSA, Caco-2-Zellen, HepaRG-Zellen, 24 h Inkubation	0, 3, 9, 28, 40, 80 µg/cm^2^ (0, 5, 15, 48, 68, 136 µg Al/ml)	–	Zytotoxizität (Zellzahl, aktive Caspase-3): –	–	n. u.	Jalili et al. 2022
Oxidative DNA-Schäden (Comet-Assay mit FPG und Endo III, alkalisch)	**Al_2_O_3_** (16,7 nm), k. A. zur Modifikation, suspendiert in Wasser, HEK293-Zellen, humane Lymphozyten (2 ♂ Spender, 24 a), 3 h Inkubation	0, 100 µg/ml (0, 53 µg Al/ml)	–	Zytotoxizität (Zellviabilität): –	–	n. u.	Demir et al. 2013 b
Oxidative DNA-Schäden (Comet-Assay mit FPG, alkalisch)	**Al_2_O_3_** (20 nm), γ-Modifikation, suspendiert in Wasser mit 0,05 % BSA, Caco-2-Zellen, HepaRG-Zellen, 5, 24 h Inkubation	0, 3, 9, 28, 40, 80 µg/cm^2^ (0, 5, 15, 48, 68, 136 µg Al/ml)	5 h: Caco-2: bei 15 und 48 µg Al/ml; HepaRG: nur „hedgehogs“ beobachtet; 24 h: nur Caco-2: 5, 15 und 48 µg Al/ml	Zytotoxizität (Zellzahl, aktive Caspase-3): – (nur nach 24 h untersucht); intrazelluläre ROS-Induktion: –, aber in Caco-2-Zellen starke Schwankung; am höchsten bei 5, 15 und 136 µg Al/ml	+	n. u.	Jalili et al. 2022
DNA-Schäden (Comet-Assay, alkalisch)	**Al_2_O_3_** (Partikelgröße: < 50 nm), γ-Modifikation, k. A. zum Lösungsmittel, humane Lymphozyten, 3 Spender, 20–25 a, vermutlich 24 h Inkubation	0, 50, 75, 100 µg/ml (27, 40, 53 µg Al/ml)	–	Zytotoxizität: ab 25 µg/ml (MTT), ab 100 µg/ml (LDH-Freisetzung)	–	n. u.	Rajiv et al. 2016
DNA-Schäden (Comet-Assay, alkalisch)	**Al_2_O_3_** (30–60 nm), k. A. zur Modifikation, suspendiert in Wasser, HepG2-Zellen, 24, 48 h Inkubation	0, 25, 50, 150, 450 µg/ml (13, 27, 80, 239 µg Al/ml)	24 und 48 h: ab 27 µg Al/ml	Zytotoxizität (MTT, NRU): 24 h: ab 80 µg Al/ml, 48 h: ab 27 µg Al/ml, Caspase-3-Aktivität: 24 h: bei 239 µg Al/ml, 48 h: ab 90 µg Al/ml, die publizierten Abbildungen zeigen keine schweifförmigen Kometen, Untersuchungen zu Markern für oxidativen Stress siehe Abschnitt 2.3.	+	n. u.	Alarifi et al. 2015
DNA-Schäden (Comet-Assay, alkalisch)	**Al_2_O_3_** (16,7 nm), k. A. zur Modifikation, suspendiert in Wasser, HEK293-Zellen, humane Lymphozyten (2 ♂ Spender, 24 a), 3 h Inkubation	0, 1, 10, 100 µg/ml (0; 0,5; 5; 53 µg Al/ml)	–	Zytotoxizität (Ethidiumbromid, Fluoresceindiacetat): –	–	n. u.	Demir et al. 2013 a
DNA-Schäden (Comet-Assay, alkalisch)	**Al_2_O_3_** („Ionic form“), suspendiert in Wasser, HEK293-Zellen, humane Lymphozyten (2 ♂, 24 a), 3 h Inkubation	0, 1, 10, 100 µg/ml (0; 0,5; 5; 53 µg Al/ml)	–	Zytotoxizität (Zellviabilität): –	–	n. u.	Demir et al. 2013 a
DNA-Schäden (Comet-Assay, alkalisch)	**Al_2_O_3_** (13, 50 nm), γ-Modifikation, suspendiert in Wasser, CHL-Zellen, 12, 24, 48 h Inkubation	0, 15, 30, 60 µg/ml (0, 8, 16, 32 µg Al/ml)	**13 und 50 nm**: 12 h: 32 µg Al/ml, 24 h: 16 µg Al/ml, **50 nm**: 48 h: ab 8 µg Al/ml, **13 nm**: 48 h: ab 16 µg Al/ml	Zytotoxizität: (Trypanblau) nur nach 24 h bestimmt: 6 µg Al/ml: 13 nm: 17 %, 50 nm: 27 %, 12,5 µg Al/ml: 13 nm: 26 %, 50 nm: 36 %, 25 µg Al/ml: 13 nm: 30 %, 50 nm: 47 %, 50 µg Al/ml: 13 nm: 44 %, 50 nm: 52 %, 100 µg Al/ml: 13 nm: 65 %, 50 nm: 57 %	+	n. u.	Zhang et al. 2017
DNA-Schäden (Comet-Assay, alkalisch)	**Al_2_O_3_** (20 nm), γ-Modifikation, suspendiert in Wasser mit 0,05 % BSA, Caco-2-Zellen, HepaRG-Zellen, 5, 24 h Inkubation	0, 3, 9, 28, 40, 80 µg/cm^2^ (0, 5, 15, 48, 68, 136 µg Al/ml)	–	Zytotoxizität (Zellzahl, aktive Caspase-3): – (nur nach 24 h untersucht)	–	n. u.	Jalili et al. 2022
SCE	**Al_2_O_3_** (28 nm), k. A. zur Modifikation, suspendiert in PBS, CHO-K1-Zellen, 24 h Inkubation	0, 1, 5, 10, 25 µg/ml (0, 1, 3, 5, 13 µg Al/ml)	–	Zytotoxizität: nach 24 h (MTT) ab 13 µg Al/ml, (NRU) ab 13 µg Al/ml	–	n. u.	Di Virgilio et al. 2010
CA	**Al_2_O_3_** (4,2 nm), k. A. zur Modifikation, suspendiert in Wasser, humane Lymphozyten, 4 ♂ Spender (20–24 a, Nichtraucher), 70 h Inkubation	0; 1; 12,5; 25; 50; 100; 250; 500; 1000; 2000 µg/ml (0, 1, 7, 13, 27, 53, 133, 265, 530, 1060 µg Al/ml)	–	keine Zytotoxizität festgestellt (Daten nicht dargestellt)	–	n. u.	Akbaba und Türkez 2018
CA	**Al_2_O_3_** (< 50 nm), γ-Modifikation, k. A. zum Lösungsmittel, humane Lymphozyten, 3 Spender (20–25 a), 1 h Inkubation	0, 50, 75, 100 µg/ml (0, 27, 40, 53 µg Al/ml)	–	Zytotoxizität: ab 25 µg/ml (MTT), ab 100 µg/ml (LDH-Freisetzung); laut OECD TG 473 zu kurze Inkubationszeit, zu wenig Zellen ausgezählt, falscher Toxizitätsparameter	–	n. u.	Rajiv et al. 2016
MN	**Al_2_O_3_** (4,2 nm), k. A. zur Modifikation, suspendiert in Wasser, humane Lymphozyten, 4 ♂ Spender (Alter: 20–24 a, Nichtraucher), 72 h Inkubation	0; 1; 12,5; 25; 50; 100; 250; 500; 1000; 2000 µg/ml (0, 1, 7, 13, 27, 53, 133, 265, 530, 1060 µg Al/ml)	–	keine Zytotoxizität festgestellt (Daten nicht dargestellt)	–	n. u.	Akbaba und Türkez 2018
MN	**Al_2_O_3_** (28 nm), k. A. zur Modifikation, suspendiert in PBS, CHO-K1-Zellen, 24 h Inkubation	0; 0,5; 1; 5; 10 µg/ml (0; 0,3; 1; 3; 5 µg Al/ml)	ab 0,3 µg Al/ml	Zytotoxizität: nicht nach TG; im Bereich 3–53 µg Al/ml untersucht, MTT: ab 3 µg Al/ml, NRU: ab 13 µg Al/ml	+	n. u.	Di Virgilio et al. 2010
MN	**Al_2_O_3_** (20 nm), γ-Modifikation, suspendiert in Wasser mit 0,05 % BSA, Caco-2-Zellen, HepaRG-Zellen, 24 h Inkubation	0, 3, 9, 28, 40, 80 µg/cm^2^ (0, 5, 15, 48, 68, 136 µg Al/ml)	–	Zytotoxizität (RI: replicative index): –	–	n. u.	Jalili et al. 2022

Al^0^: elementares Aluminium; Al_2_O_3_: Aluminiumoxid; Al(OH)_3_: Aluminiumhydroxid; BSA: bovines Serumalbumin; CA: Chromosomenaberrationen; Caco-2-Zellen: humane Colonadenokarzinom-Zellen; CHO-K1-Zellen: Eierstockzellen des Chinesischen Hamsters; Endo III: Endonuklease III; FPG: Formamidopyrimidin-DNA-Glykosylase; HEK293-Zellen: humane embryonale Nierenzellen; HepaRG-Zellen: immortalisierte humane Leber-Stammzellen; HepG2-Zellen: humane Leberkarzinomzellen; k. A.: keine Angaben; LDH: Lactatdehydrogenase; LM: Lösungsmittel; MI: mitotischer Index; MN: Mikronuklei; MTT: 3-(4,5-Dimethylthiazol-2-yl)-2,5-diphenyltetrazoliumbromid; NR: Nichtraucher; NRU: Neutralrot-Aufnahme; n. u.: nicht untersucht; PBS: phosphatgepufferte Kochsalzlösung; R: Raucher; SCE: Schwesterchromatidaustausch; sig.: statistisch signifikant; TG: Prüfrichtlinie (Test Guideline)

Hinweis: Dargestellt sind die statistisch signifikanten Ergebnisse, wenn nicht anders angegeben.

##### 5.6.1.1 Bakterien

Aluminium und seine Verbindungen zeigten in bakteriellen Mutagenitätstests, Tests auf differentielle Abtötung DNA-Reparatur-profizienter und -defizienter Stämme von Bacillus subtilis sowie in SOS-Chromotests in Escherichia coli keine genotoxische Wirkung (Greim 2007).

Ergänzend liegen drei Genmutationsuntersuchungen mit Salmonella-typhimurium- sowie Escherichia-coli-Stämmen vor, die alle negativ waren (Balasubramanyam et al. 2010; Pan et al. 2010; Zhang et al. 2017).

##### 5.6.1.2 Säugetierzelllinien und humane Lymphozyten

###### 5.6.1.2.1 Indikatortests

**Ultrafeines**, **elementares Aluminium **verursachte bei nicht zytotoxischen Konzentrationen eine statistisch signifikante Zunahme an phosphoryliertem Histon H2AX (γ-H2AX). Mit **ultrafeinem Aluminiumoxid** (γ-Modifikation) trat kein Effekt auf (Jalili et al. 2022). Die Phosphorylierung von H2AX geschieht im Zuge der DNA-Reparatur. Meist sind DNA-Doppelstrangbrüche der Auslöser, aber auch DNA-Schäden während der Proliferation oder der Apoptose können die Phosphorylierung von H2AX bewirken. Um zu beweisen, dass es sich zweifelsfrei um DNA-Doppelstrangbrüche handelt, muss die Kolokalisation eines zweiten Proteins (z. B. 53BP1) nachgewiesen werden (Rothkamm et al. 2015). Da dies in den vorliegenden Studien nicht geschehen ist, handelt es sich hier um nicht weiter spezifizierte DNA-Schäden.

Untersuchungen auf DNA-Schäden mittels Comet-Assay (alkalisch) liegen für ultrafeines, elementares Aluminium, mikroskaliges Aluminiumoxid sowie ultrafeines Aluminiumoxid vor. Für **ultrafeines**, **elementares Aluminium** zeigte sich nach 24-stündiger Inkubation in Caco-2- sowie HepaRG-Zellen eine Zunahme der „tail intensity“ ab 90 µg Al/ml ohne Zytotoxizität (Jalili et al. 2022). Für **mikroskaliges Aluminiumoxid** ließ sich in CHL-Zellen ab 32 µg Al/ml nach 12- und 24-stündiger Inkubation sowie ab 16 µg Al/ml nach 48-stündiger Inkubation ein positives Ergebnis beobachten. Zytotoxizität trat nach 24 Stunden ab 12,5 µg/ml auf (Zhang et al. 2017). **Ultrafeines Aluminiumoxid** wurde in sechs Comet-Assays in vitro untersucht, wovon vier negativ waren. In einem Test (Alarifi et al. 2015) fehlt eine Angabe zur Modifikation, sodass es sich bei der Verbindung auch um α-Aluminiumoxid handeln kann. Weiterhin zeigt die Abbildung in der Publikation keinen schweifförmigen Kometen. Dieser Test wird nicht für die Bewertung herangezogen. In einem weiteren Comet-Test wurden mit unterschiedlichen Inkubationszeiten (12, 24, 48 Stunden) und unterschiedlichen Partikelgrößen (13, 50 nm, γ-Modifikation) positive Ergebnisse in CHL-Zellen beobachtet. Die geringste Konzentration, die Effekte zeigte, lag bei 8 µg Al/ml nach 48-stündiger Inkubation. Zytotoxizität wurde nur nach 24 Stunden untersucht, sie trat bei geringeren Konzentrationen als die DNA-Schäden auf. Weiterhin wurden, meist nach 24 und 48 Stunden, eine Abnahme von GSH, SOD-Aktivität und TAC sowie eine Zunahme von MDA als Biomarker für oxidativen Stress gemessen (Zhang et al. 2017, siehe auch Abschnitt 2.3).

Oxidative DNA-Basenschäden wurden für **ultrafeines**, **elementares Aluminium** nach einer 24-stündigen Inkubation in HepaRG-, nicht aber in Caco-2-Zellen, ab der geringsten eingesetzten Konzentration beobachtet. Zytotoxizität trat nicht auf. Nach fünfstündiger Inkubation war der Test nur in Caco-2-Zellen positiv, Zytotoxizität wurde hier nicht untersucht. Für **ultrafeines Aluminiumoxid** wurden nicht konzentrationsabhängige positive Befunde bei nicht zytotoxischen Konzentrationen beobachtet (Jalili et al. 2022). Eine zweite Untersuchung zu oxidativen Basenschäden mit **ultrafeinem Aluminiumoxid** (Demir et al. 2013 b) kann aufgrund fehlender Angaben zur Modifikation nicht zur Bewertung herangezogen werden.

Eine Untersuchung auf Schwesterchromatidaustausche (SCE) mit **ultrafeinem Aluminiumoxid** (Di Virgilio et al. 2010) kann aufgrund fehlender Angaben zur Modifikation nicht zur Bewertung herangezogen werden.

###### 5.6.1.2.2 Aneugene und klastogene Wirkungen 

Zwei Tests auf Chromosomenaberrationen in humanen Lymphozyten mit **ultrafeinem Aluminiumoxid** waren negativ. Es wurde einmal eine Stunde mit Konzentrationen bis 53 µg Al/ml inkubiert oder 70 Stunden mit Konzentrationen bis 1060 µg Al/ml. Nur für die Kurzzeituntersuchung liegt eine Angabe zur Modifikation (γ-Modifikation) vor (Akbaba und Türkez 2018; Rajiv et al. 2016).

Zur Mikronukleus-Bildung sind eine Untersuchung mit **ultrafeinem**, **elementarem Aluminium** sowie drei mit **ultrafeinem Aluminiumoxid** publiziert worden (Akbaba und Türkez 2018; Di Virgilio et al. 2010; Jalili et al. 2022). Nur eine Untersuchung war positiv. In CHO-K1-Zellen ließen sich nach einer 24-stündigen Inkubation mit **ultrafeinem Aluminiumoxid **(k. A. zur Modifikation) in Konzentrationen von 0,3–5 µg Al/ml erhöhte Werte an Mikronuklei erkennen. Im MTT-Test wurde Zytotoxizität nach 24 Stunden im gesamten untersuchten Bereich von 3–53 µg Al/ml festgestellt. Für die Konzentrationen 0,3 und 0,5 µg/ml liegt keine Angabe zur Zytotoxizität vor (Di Virgilio et al. 2010). Nach OECD-Prüfrichtlinie 487 sind die hier verwendeten Marker nicht geeignet, um Zytotoxizität in diesem Testsystem hinreichend abzubilden. Der Test ist somit fraglich bewertungsrelevant.

###### 5.6.1.2.3 Genmutationstest

Die Aluminiumverbindungen hatten in den bisher verfügbaren Untersuchungen in Säugetierzellen keine mutagene Wirkung (ATSDR 2008; COT 2013; EFSA 2008; Greim 2007; NEG und DECOS 2011). Dies wird bestätigt durch einen neuen nach OECD-Prüfrichtlinie 476 durchgeführten Tk^+/–^-Mutationstest mit **Aluminiumhydroxid** in Konzentrationen bis 265 µg Al/ml in An- und Abwesenheit metabolischer Aktivierung. Es wurde bis zur Zytotoxizität getestet (Covance Laboratories Ltd 2010 b).

#### 5.6.2 In vivo

Im Nachtrag von 2007 (Greim 2007) sind keine Untersuchungen mit schwerlöslichen Aluminiumverbindungen aufgeführt. Untersuchungen zu elementarem Aluminium sowie mikroskaligen und ultrafeinen schwerlöslichen Aluminiumverbindungen sind nachfolgend aufgelistet (siehe Tabelle 13).

**Tab. 13:** In-vivo-Studien zur Genotoxizität mit ultrafeinem, elementarem Aluminium, mikroskaligen und ultrafeinen schwerlöslichen Aluminiumverbindungen

**Endpunkt**	**Testsystem**	**Exposition**	**Ergebnis**	**Zytotoxizität/Anmerkungen**	**Literatur**
**Ultrafeines, elementares Aluminium**					
DNA-Schäden (Comet-Assay, alkalisch), Leber, Zwölffingerdarm, Milz, Knochenmark, Nieren, Blut	Ratte, Sprague Dawley, je 5 ♂	3 × (0, 24 u. 45 h), **Al^0^** (18 nm), 0; 6; 12,5; 25 mg Al/kg KG u. d, Wasser mit 0,05 % BSA, Gavage, untersucht 3 h nach letzter Gabe	–	Zytotoxizität nur im Knochenmark untersucht (PCE/NCE-Verhältnis unverändert), Dosiswahl nicht begründet	Jalili et al. 2020
oxidative DNA-Schäden (Comet-Assay mit FpG, alkalisch), Leber, Zwölffingerdarm, Milz, Knochenmark, Nieren, Blut	Ratte, Sprague Dawley, je 5 ♂	3 × (0, 24 u. 45 h), **Al^0^** (18 nm), 0; 6; 12,5; 25 mg Al/kg KG u. d, Wasser mit 0,05 % BSA, Gavage, untersucht 3 h nach letzter Gabe	–	keine Zytotoxizität untersucht, außer Knochenmark (PCE/NCE-Verhältnis unverändert), Dosiswahl nicht begründet	Jalili et al. 2020
MN, Colon, Knochenmark	Ratte, Sprague Dawley, je 5 ♂	3 × (0, 24 u. 45 h), **Al^0^** (18 nm), 0; 6; 12,5; 25 mg Al/kg KG u. d, Wasser mit 0,05 % BSA, Gavage, untersucht 3 h nach letzter Gabe	–	keine Zytotoxizität im Knochenmark (PCE/NCE-Verhältnis unverändert); Colon: kein Anstieg mitotischer und apoptotischer Zellen (k. w. A.), keine validierte Methode für MN, Dosiswahl nicht begründet	Jalili et al. 2020
**Mikroskalige, schwerlösliche Aluminiumverbindungen**					
SMART	D. melanogaster Larven	gesamte Larvalentwicklung, **Al_2_O_3_**, k. A. zur Modifikation, suspendiert in Wasser, 0; 0,1; 1; 5; 10 mM, Futter	–	keine Zytotoxizität untersucht	Demir et al. 2013 b
DNA-Schäden (Comet-Assay, alkalisch), peripheres Blut	Ratte, Wistar, je 5 ♀	einmalig, **Al_2_O_3_**, k. A. zur Modifikation, suspendiert in 1 % Tween, 0, 500, 1000, 2000 mg/kg KG (0, 265, 529, 1059 mg Al/kg KG), Gavage, untersucht nach 4, 24, 48, 72 h	–	keine Zytotoxizität (Zellviabilität > 80 %)	Balasubramanyam et al. 2009 a
CA, Knochenmark	Ratte, Wistar, je 5 ♀	einmalig, **Al_2_O_3_**, k. A. zur Modifikation, suspendiert in 1 % Tween, 0, 500, 1000, 2000 mg/kg KG (0, 265, 529, 1059 mg Al/kg KG), Gavage, untersucht nach 18, 24 h	–	keine Zytotoxizität (MI nicht reduziert)	Balasubramanyam et al. 2009 b
MN, Knochenmark	Ratte, Wistar, je 5 ♀	einmalig, **Al_2_O_3_**, k. A. zur Modifikation, suspendiert in 1 % Tween, 0, 500, 1000, 2000 mg/kg KG (0, 265, 529, 1059 mg Al/kg KG), Gavage, untersucht nach 30, 48 h	–	keine Zytotoxizität (PCE/NCE-Verhältnis unverändert)	Balasubramanyam et al. 2009 b
MN, peripheres Blut	Ratte, Wistar, je 5 ♀	einmalig, **Al_2_O_3_**, k. A. zur Modifikation, suspendiert in 1 % Tween, 0, 500, 1000, 2000 mg/kg KG u. d (0, 265, 529, 1059 mg Al/kg KG), Gavage, untersucht nach 48, 72 h	–	keine Zytotoxizität (PCE/NCE-Verhältnis unverändert)	Balasubramanyam et al. 2009 a
MN, Knochenmark	Ratte, Sprague Dawley, je 6 ♂	2 × (0, 24 h), **Al(OH)_3_**, k. A. zur Modifikation, suspendiert in 1 % Carboxymethylcellulose in Wasser, 0, 500, 1000, 2000 mg/kg KG u. d (0, 173, 346,692 mg Al/kg KG u. d), Gavage, untersucht nach 24 h, OECD TG 474	–	keine Zytotoxizität (PCE/NCE-Verhältnis unverändert; keine systemische Toxizität)	Covance Laboratories Ltd 2010 a
MN, Knochenmark	Maus, ICR, je 5 ♀, 5 ♂	2 × (0, 24 h), **Al_2_O_3_**, k. A. zur Modifikation, 0, 300, 600, 1200 µg/kg KG u. d (0, 159, 318, 635 µg Al/kg KG u. d), i.p., untersucht 6 h nach letzter Gabe	–	keine Zytotoxizität (PCE/NCE-Verhältnis unverändert), Probenahme zu früh	Zhang et al. 2017
**Ultrafeine, schwerlösliche Aluminiumverbindungen**					
SMART	D. melanogaster Larven	gesamte Larvalentwicklung, **Al_2_O_3_** (16,7 nm), k. A. zur Modifikation, suspendiert in Wasser, 0; 0,1; 1, 5; 10 mM, Futter	–	keine Zytotoxizität untersucht	Demir et al. 2013 b
DNA-Schäden (Comet-Assay, alkalisch), Leber, Zwölffingerdarm, Magen	Ratte, Sprague Dawley, je 6 ♂	3 × (0, 24 u. 45 h), **Al_2_O_3_** (< 90 µm), k. A. zur Modifikation, suspendiert in Wasser mit 1 % Carboxymethylcellulose, 0, 500, 1000, 2000 mg/kg KG u. d (0, 265, 529, 1059 mg Al/kg KG u. d), Gavage, untersucht 3 h nach letzter Behandlung (48 h), OECD TG 489	–	keine Zytotoxizität (PCE/NCE-Verhältnis unverändert; keine systemische Toxizität)	Covance Laboratories Ltd 2021
DNA-Schäden (Comet-Assay, alkalisch), Gehirn, Leber, Milz, Nieren, Hoden	Maus, Swiss-Albino, je 5 ♂	5 d, **Al_2_O_3_** (< 50 nm), vermutlich γ-Modifikation, suspendiert in Wasser, 0, 15, 30, 60 mg/kg KG u. d (0, ca. 8, 16, 32 mg Al/kg KG u. d.), Gavage	+ ab 15 mg/kg KG (8 mg Al/kg KG) in Gehirn, Milz, Hoden; + ab 30 mg/kg KG (16 mg Al/kg KG) in Leber, Nieren	keine Zytotoxizität untersucht, allerdings Toxizitätsmarker verändert ab 16 mg Al/kg KG siehe Abschnitt 5.2.2, k. A. zu eingesetzten Zelltypen	De et al. 2020
DNA-Schäden (Comet-Assay, alkalisch), peripheres Blut	Ratte, Wistar, je 5 ♀	einmalig, **Al_2_O_3_** (30 nm, 40 nm), k. A. zur Modifikation, suspendiert in 1 % Tween, 0, 500, 1000, 2000 mg/kg KG (0, 265, 529, 1059 mg Al/kg KG), Gavage, untersucht nach 4, 24, 48, 72 h	+ 30 nm (4, 24, 48 h): ab 1000 mg/kg KG (529 mg Al/kg KG), 40 nm (4, 24 h): ab 1000 mg/kg KG (529 mg Al/kg KG), 40 nm (48 h): ab 2000 mg/kg KG (1059 mg Al/kg KG)	keine Zytotoxizität (Zellviabilität > 80 %)	Balasubramanyam et al. 2009 a
DNA-Schäden (Comet-Assay, alkalisch), Gehirn	Ratte, Albino, je 3 ♂	einmalig, **Al_2_O_3_** (< 13 nm), k. A. zur Modifikation, suspendiert in physiologischer Kochsalzlösung, 0, 3900, 6400, 8500 mg/kg KG (0, 2064, 3387, 4499 mg Al/kg KG, i.p., untersucht nach 48 h	+ ab 6400 mg/kg KG (3387 mg Al/kg KG)	k. A. zur Zytotoxizität	Morsy et al. 2016
DNA-Schäden (Comet-Assay, alkalisch) Gehirn	Ratte, Albino, je 3 ♂	28 d, im Abstand von 2 d, **Al_2_O_3_** (< 13 nm), k. A. zur Modifikation, suspendiert in physiologischer Kochsalzlösung, 0, 1300 mg/kg KG u. d (0, 688 mg Al/kg KG u. d), i.p., untersucht nach 1, 3, 7, 14, 28 d	+	k. A. zur Zytotoxizität	Morsy et al. 2016
DNA-Schäden (Comet-Assay, alkalisch), Leber, Zwölffingerdarm, Milz, Knochenmark, Nieren, Blut	Ratte, Sprague Dawley, je 5 ♂	3 × (0, 24 u. 45 h), **Al_2_O_3_** (20 nm), γ-Modifikation, suspendiert in Wasser mit 0,05 % BSA, 0; 6; 12,5; 25 mg/kg KG u. d (0; 3,31; 6,63; 13,25 mg Al/kg KG u. d), Gavage, untersucht nach 3 h	– Leber, Zwölffingerdarm, Milz, Nieren, Blut; + Knochenmark bei 25 mg/kg KG u. d (13,25 mg Al/kg KG u. d)	Zytotoxizität nur im Knochenmark untersucht (PCE/NCE-Verhältnis unverändert), Dosiswahl nicht begründet	Jalili et al. 2020
oxidative DNA-Schäden (Comet-Assay mit FpG, alkalisch), Leber, Zwölffingerdarm, Milz, Knochenmark, Nieren, Blut	Ratte, Sprague Dawley, je 5 ♂	3 × (0, 24 u. 45 h), **Al_2_O_3_** (20 nm), γ-Modifikation, 0; 6; 12,5; 25 mg/kg KG u. d (0; 3,31; 6,63; 13,25 mg Al/kg KG u. d), suspendiert in Wasser mit 0,05 % BSA, Gavage, untersucht nach 3 h	–	Zytotoxizität nur im Knochenmark untersucht (PCE/NCE-Verhältnis unverändert), Dosiswahl nicht begründet	Jalili et al. 2020
CA, Knochenmark	Ratte, Wistar, je 5 ♀	einmalig, **Al_2_O_3_** (30 nm, 40 nm), k. A. zur Modifikation, suspendiert in 1 % Tween, 0, 500, 1000, 2000 mg/kg KG (0, 265, 529, 1059 mg Al/kg KG), Gavage, untersucht nach 18, 24 h	+ Gesamt-Aberrationen (ohne Gaps): 30 nm: ab 1000 mg/kg KG (529 mg Al/kg KG); 40 nm: 2000 mg/kg KG (1059 mg Al/kg KG)	keine Zytotoxizität (MI nicht reduziert)	Balasubramanyam et al. 2009 b
MN, Knochenmark	Ratte, Sprague Dawley, je 6 ♂	3 × (0, 24 u. 45 h), **Al_2_O_3_** (< 90 µm), k. A. zur Modifikation, Reinheit: 98,7 %, suspendiert in Wasser mit 1 % Carboxymethylcellulose, 0, 500, 1000, 2000 mg/kg KG u. d (0, 265, 529, 1059 mg Al/kg KG u. d), Gavage, untersucht 3 h nach letzter Behandlung (48 h), OECD TG 474	–	keine Zytotoxizität (PCE/NCE-Verhältnis unverändert; keine systemische Toxizität)	Covance Laboratories Ltd 2021
MN, Knochenmark, Colon	Ratte, Sprague Dawley, je 5 ♂	3 × (0, 24 u. 45 h), **Al_2_O_3_** (20 nm), γ-Modifikation, suspendiert in Wasser mit 0,05 % BSA, 0; 6; 12,5; 25 mg/kg KG u. d (0, 3,31; 6,63; 13,25 mg Al/kg KG u. d), Gavage, untersucht 3 h nach der letzten Dosis	–	keine Zytotoxizität im Knochenmark (PCE/NCE-Verhältnis unverändert); Colon: kein Anstieg von mitotischen und apoptotischen Zellen (k. w. A.), keine validierte Methode für MN, Dosiswahl nicht begründet	Jalili et al. 2020
MN, Knochenmark	Ratte, Wistar, je 5 ♀	einmalig, **Al_2_O_3_** (30 nm, 40 nm), k. A. zur Modifikation, Reinheit > 90 %, suspendiert in 1 % Tween, 0, 500, 1000, 2000 mg/kg KG. (0, 265, 529, 1059 mg Al/kg KG), Gavage, untersucht nach 30, 48 h	+ 30 nm, 40 nm (30, 48 h): ab 1000 mg/kg KG (529 mg Al/kg KG)	keine Zytotoxizität (PCE/NCE-Verhältnis unverändert)	Balasubramanyam et al. 2009 b
MN, peripheres Blut	Ratte, Wistar, je 5 ♀	einmalig, **Al_2_O_3_** (30 nm, 40 nm), k. A. zur Modifikation, Reinheit > 90 %, suspendiert in 1 % Tween, 0, 500, 1000, 2000 mg/kg KG (0, 265, 529, 1059 mg Al/kg KG), Gavage, untersucht nach 48, 72 h	+ 30 nm, 40 nm (48 h, 72 h): ab 1000 mg/kg KG u. d (529 mg Al/kg KG)	keine Zytotoxizität (PCE/NCE-Verhältnis unverändert)	Balasubramanyam et al. 2009 a
MN, Knochenmark	Maus, ICR, je 5 ♀, 5 ♂	zweimalig, **Al_2_O_3_** (13 nm): k. A. zur Modifikation, 0, 300, 600, 1200 µg/kg KG u. d (0, 159, 318, 635 µg Al/kg KG u. d), i.p., untersucht 6 h nach letzter Gabe	–	keine Zytotoxizität (PCE/NCE-Verhältnis unverändert), Probenahme zu früh	Zhang et al. 2017

Al^0^: elementares Aluminium; Al_2_O_3_: Aluminiumoxid; Al(OH)_3_: Aluminiumhydroxid; BSA: bovines Serumalbumin; CA: Chromosomenaberrationen; d: Tag; D: Drosophila; FPG: Formamidopyrimidin-DNA-Glykosylase; k. A.: keine Angabe; MI: Mitotischer Index; MN: Mikronuklei; NCE: normochromatische Erythrozyten; PCE: polychromatische Erythrozyten; SCE: Schwesterchromatidaustausch; SMART: Somatic mutation and recombination test; TG: Prüfrichtlinie

Hinweis: Dargestellt sind die statistisch signifikanten Ergebnisse, wenn nicht anders angegeben.

##### 5.6.2.1 Indikatortests

**Ultrafeines**, **elementares Aluminium** verursachte keine (oxidativen) DNA-Schäden in verschiedenen Organen, **mikroskalige, schwerlösliche Aluminiumverbindungen** keine im peripheren Blut von Ratten.

Mit **ultrafeinen, schwerlöslichen Aluminiumverbindungen **liegen sieben Untersuchungen auf DNA-Schäden und oxidative Basenschäden an Ratten vor, wobei fünf positive Ergebnisse mittels des alkalischen Comet-Assays erzielten (Balasubramanyam et al. 2009 a; De et al. 2020; Jalili et al. 2020; Morsy et al. 2016). In drei dieser positiven Untersuchungen wurde einmalig oder wiederholt eine sehr hohe Dosis eingesetzt (≥ 1000 mg/kg KG (529 mg Al/kg KG)) oder es fehlen Angaben zur Zytotoxizität (De et al. 2020). Zudem wurde eine für die Exposition am Arbeitsplatz nicht relevante Verabreichungsform (i.p. Gabe) gewählt (Morsy et al. 2016), was die Belastbarkeit der Studien stark einschränkt. In einer weiteren Studie mit positivem Ergebnis im peripheren Blut wurden gleichfalls hohe Dosen eingesetzt, allerdings trat hierbei keine Zytotoxizität auf (Balasubramanyam et al. 2009 a). An der Studie wurde die ungenügende Charakterisierung und Reinheit der eingesetzten Aluminiumverbindung kritisiert (siehe unten Diskussion Mikronukleustest). Bei einer Untersuchung mit der niedrigen Konzentration von 25 mg **Aluminiumoxid**/kg KG und Tag (13,25 mg Al/kg KG und Tag) zeigte sich nach dreimaliger Gabe im Knochenmark von Ratten eine Zunahme der „tail intensity“ im nicht zytotoxischen Bereich. In Leber, Zwölffingerdarm, Milz, Nieren und Blut trat kein Effekt auf. Die Untersuchung auf oxidative DNA-Schäden in diesen Geweben und auch im Knochenmark ergab negative Ergebnisse (Jalili et al. 2020). Den positiven Ergebnissen steht eine negative Untersuchung nach OECD-Prüfrichtlinie 489 mit **Aluminiumoxid** unter ähnlichen Versuchsbedingungen (Gabe, Spezies, Untersuchungszeitraum) in Dosen bis 2000 mg Aluminiumoxid/kg KG und Tag (1059 mg Al/kg KG u. Tag) in Leber, Zwölffingerdarm und Magen gegenüber. Eine Untersuchung des Knochenmarks wurde jedoch nicht durchgeführt (Covance Laboratories Ltd 2021).

##### 5.6.2.2 Drosophila-Tests

**Mikroskalige **und** ultrafeine schwerlösliche Aluminiumverbindungen **waren negativ im Test auf somatische Mutationen und Rekombinationen in Drosophila (Demir et al. 2013 b).

##### 5.6.2.3 Klastogene und aneugene Wirkung

**Ultrafeines**, **elementares Aluminium **war in einem Mikronukleustest im Knochenmark und Colon von Ratten negativ (Jalili et al. 2020).

Für **mikroskalige, schwerlösliche Aluminiumverbindungen **liegen vier negative Mikronukleustests vor, einer davon nach OECD-Prüfrichtlinie 474 (Covance Laboratories Ltd 2010 a). Anzumerken ist, dass bei einem der Tests (Zhang et al. 2017) die Zellen zu einem zu frühen Zeitpunkt (sechs Stunden nach letzter Behandlung bei zweimaliger Gabe) untersucht wurden, wodurch das Ergebnis beeinflusst werden könnte. Diese Untersuchung ist somit nicht valide.

Einmalige Gabe per Schlundsonde von **ultrafeinem Aluminiumoxid** (Modifikation nicht bekannt) führte bei Wistar-Ratten ab 1000 mg/kg KG und Tag (529 mg Al/kg KG) zur Induktion von Chromosomenaberrationen im Knochenmark. Der mitotische Index als Maß für die Zytotoxizität war im Vergleich zur Kontrolle nicht reduziert (Balasubramanyam et al. 2009 b). An der Studie wurde die ungenügende Charakterisierung und Reinheit der eingesetzten Aluminiumverbindung kritisiert (siehe unten Diskussion Mikronukleustest).

Es liegen zwei negative Mikronukleustests (Jalili et al. 2020; Zhang et al. 2017) mit **ultrafeinem Aluminiumoxid** vor sowie zusätzlich ein negativer Test nach OECD-Prüfrichtlinie 474 (Covance Laboratories Ltd 2021).

In den zwei positiven Studien ließ sich die Bildung von Mikronuklei in Wistar-Ratten nach einmaliger Gabe von **Aluminiumoxid** (keine Angabe zur Modifikation, Reinheit > 90 %) per Schlundsonde in der mittleren und hohen Dosis von 1000 und 2000 mg/kg KG (529 und 1059 mg Al/kg KG) im Knochenmark und im peripherem Blut beobachten. Das PCE/NCE-Verhältnis war nicht vermindert, was anzeigt, dass keine Zytotoxizität auftrat (Balasubramanyam et al. 2009 a, b). Die Modifikation der eingesetzten Aluminiumoxidverbindung ist jedoch nicht angegeben, somit ist unklar, ob es sich um eine **schwerlösliche Aluminiumoxidverbindung** (γ/δ-Aluminiumoxid) oder um das unlösliche **Korund** handelt. Eine Nachfrage bei den Studienautoren blieb unbeantwortet. Die Autoren der validen negativen Mikronukleus-Untersuchung (Covance Laboratories Ltd 2021) bemängelten an den Studien von Balasubramanyam et al. (2009 a, b), dass die darin verwendeten ultrafeinen Partikel nicht ausreichend charakterisiert waren und einen hohen Gehalt an Verunreinigungen aufwiesen. Der oben erwähnte valide Mikronukleustest nach OECD-Prüfrichtlinie 474 im Knochenmark mit dreimaliger Gabe von 0, 500, 1000 oder 2000 mg **Aluminiumoxid**/kg KG und Tag (0, 265, 529, 1059 mg Al/kg KG und Tag) per Schlundsonde in Sprague-Dawley-Ratten erzielte ein negatives Ergebnis. Zytotoxizität trat nicht auf. Bei einer Nachweisgrenze von 50 µg Al/l Plasma konnte nur bei einem Tier Aluminium gesichert nachgewiesen werden (Covance Laboratories Ltd 2021; Labcorp Early Development Laboratories Ltd 2023). Untersuchungen zur Verteilung von **ultrafeinem Aluminiumoxid** nach oraler Gabe zeigen eine Verteilung in verschiedenen Organen (Abschnitt 3.1.6), was als Hinweis gewertet werden kann, dass bei den in dieser Studie eingesetzten Dosierungen das Zielgewebe vermutlich erreicht wird.

#### 5.6.3 Gesamtfazit zur Genotoxizität in vitro und in vivo

Bestätigend zu den bisherigen Untersuchungen waren Aluminium und seine schwerlöslichen Verbindungen in Bakterien und Säugetierzellen nicht mutagen. Dies ist im Einklang mit den Ergebnissen anderer Metalle und Metallsalze (mit Ausnahme von Chrom (VI)-Verbindungen), welche ebenso nicht oder nur schwach mutagen in bakteriellen Testsystemen wirken (Beyersmann und Hartwig 2008).

**Ultrafeines elementares Aluminium** hat in vitro in drei von vier Indikatortests eine (oxidative) DNA-schädigende Wirkung (γ-H2AX, Comet-Assay) bei teilweise nicht zytotoxischen Konzentrationen. Mikronuklei wurden in vitro und in vivo jedoch nicht induziert. Für **mikroskalige Aluminiumverbindungen** ist nur eine In-vitro-Untersuchung auf DNA-Schäden (Comet-Assay) verfügbar. Diese war positiv, allerdings trat gleichzeitig Zytotoxizität auf (nur nach 24 h untersucht), sodass nicht zwischen genotoxischer und zytotoxischer Wirkung unterschieden werden kann. Alle In-vivo-Untersuchungen mit ultrafeinem elementarem Aluminium und mikroskaligen Aluminiumverbindungen verliefen negativ.

Für **ultrafeine Aluminiumverbindungen** zeigen Indikatortests auf (oxidative) DNA-schädigende Wirkung ein heterogenes Bild. Neben validen negativen Untersuchungen gibt es auch positive Ergebnisse bei niedrigen, zytotoxischen Konzentrationen (in vitro; Zhang et al. 2017) oder bei fehlender Konzentrations-Wirkungs-Beziehung (in vitro; Jalili et al. 2022). Allerdings war bei einer positiven Untersuchung auch nachweislich keine Zytotoxizität vorhanden (in vivo; Jalili et al. 2020). Dies kann durch die Bildung von ROS, die für verschiedene Aluminiumverbindungen gezeigt wurde, ausgelöst worden sein (siehe Abschnitt 2.3). Da ROS je nach Generierungsweg zu einem unterschiedlichen Schadensspektrum führen können, widerspricht dies nicht den negativen In-vivo-Tests auf oxidative Basenschäden. Untersuchungen zur klastogenen und aneugenen Wirkung waren in vitro überwiegend negativ. Von nur einer Arbeitsgruppe wurden positive Ergebnisse im Chromosomenaberrations-Test und zwei Mikronukleustests berichtet (Balasubramanyam et al. 2009 a, b). Zytotoxizität wurde nicht festgestellt. Die Charakterisierung der eingesetzten ultrafeinen Partikel war jedoch unzureichend und die Partikel wiesen im Vergleich zu anderen negativen Studien Verunreinigungen auf. Den positiven stehen drei negative Mikronukleus-Untersuchungen an Ratten und Mäusen gegenüber, darunter auch eine nach OECD-Prüfrichtlinie 474 mit mehrfacher Gabe von Aluminiumoxid bis 1059 mg Al/kg KG und Tag (Covance Laboratories Ltd 2021).

Zusammenfassend lassen sich für elementares Aluminium sowie schwerlösliche Aluminiumverbindungen zwar vereinzelte, vermutlich durch ROS ausgelöste, DNA-schädigende Wirkungen in Indikatortests beobachten. Untersuchungen zur Induktion von Chromosomenaberrationen und Mikronuklei geben jedoch keine Hinweise auf eine direkte genotoxische Wirkung für elementares Aluminium und schwerlösliche Aluminiumverbindungen.

### 5.7 Kanzerogenität

Nach zweijähriger intratrachealer Instillation von 30 oder 60 mg ultrafeinem **Aluminiumoxid** und **Aluminiumsilikat** traten Lungentumoren bei Ratten (Greim 2007) auf. Es liegen keine neueren Studien zur Kanzerogenität mit **elementarem Aluminium** oder **schwerlöslichen Aluminiumverbindungen** vor.

## 6 Bewertung

Beim Menschen treten nach inhalativer Exposition Effekte am respiratorischen System sowie Neurotoxizität auf. Nach chronischer inhalativer Exposition steht bei der Ratte die Lungentoxizität im Vordergrund. Die durch den Partikelüberladungseffekt verursachte Überlastung der Lungenclearance kann zu einer kanzerogenen Wirkung in der Lunge führen.

**MAK-Wert.**

**A-Fraktion**

Bei 0,47–0,76 mg Schweißrauch/m^3^ (Al-haltiger Schweißrauch mit Al-Anteil der Schweißelektrode von min. 90 %, alveolengängige Fraktion) wurden keine neurotoxischen Wirkungen an Aluminium-Schweißern beobachtet (Buchta et al. 2003; Kiesswetter et al. 2009; Letzel et al. 2006). Es ist nicht untersucht worden, wie hoch der Al-Anteil im Schweißrauch ist. Falls Aluminium unverändert vorliegt, ist die Konzentration ca. 0,5 mg Al/m^3^, wird davon ausgegangen, dass Aluminium als Al_2_O_3_ vorliegt, ist der Al-Anteil im Schweißrauch ca. 50 % also ca. 0,25 mg Al/m^3^. Im gleichen Konzentrationsbereich zeigten sich erhöhte emphysematöse, bullöse und bronchitische Lungenveränderungen, die durch Aluminium, aber auch durch Koexposition gegen Zigarettenrauch und Ozon verursacht sein könnten (siehe Abschnitt 4.2.1). Eine NOAEC für Lungenveränderungen lässt sich daher aus den vorliegenden Humandaten nicht ableiten. Dafür wird eine Tierstudie verwendet:

In einer 28-Tage-Inhalationsstudie an männlichen Wistar-Ratten zeigten sich bei Exposition gegen **ultrafeinen Aluminiumoxyhydroxid**-Staub bei der höchsten Konzentration von 28 mg/m^3^ (11,03 bzw. 12,29 mg Aluminium/m^3^) erhöhte Entzündungswerte in der BALF, leichte Hyperzellularität und fokale septale Kollagenablagerungen in der bronchoalveolären Region, ein erhöhtes absolutes Lungengewicht, erhöhte absolute und relative Gewichte der Lungen-assoziierten Lymphknoten und eine verringerte Lungen-Clearance. Die NOAEC beträgt 1,18 bzw. 1,32 mg Al/m^3 ^(siehe Abschnitt 5.2.1). Da keine Studie mit längerer Expositionszeit vorliegt, ist nicht auszuschließen, dass nach chronischer Inhalation eine Wirkungsverstärkung mit der Zeit eintritt und somit die chronische NAEC (Extrapolation subakut zu chronisch 1:6) im Bereich von 0,2 mg Al/m^3^ liegt. Unter Berücksichtigung des erhöhten Atemvolumens am Arbeitsplatz im Vergleich zu inhalativen Tierversuchen in Ruhe (1:2) ergibt sich eine Konzentration von 0,1 mg Al/m^3^. Da dieser Wert aus tierexperimentellen Untersuchungen stammt (Übertragung Tier-Mensch 1:2), kann entsprechend der Vorgehensweise der Kommission (siehe Abschnitt I der MAK- und BAT-Werte-Liste, DFG 2024) ein MAK-Wert für Aluminium und seine schwerlöslichen Verbindungen von 0,05 mg Al/m^3^ (A-Fraktion) abgeleitet werden. Dieser Wert stellt einen Worst-Case dar, da er aus ultrafeinen Partikeln abgeleitet ist, am Arbeitsplatz aber in der Regel Mischexpositionen aus ultrafeinen und mikroskaligen Partikeln vorliegen. Dieser MAK-Wert ist deutlich niedriger als die NOAEC für neurotoxische Wirkung (0,25–0,5 mg Al/m^3^) aus Studien am Arbeitsplatz.

Der MAK-Wert von 0,05 mg/m^3^ A ist niedriger als der für Aluminium (Dichte 2,7 g/cm^3^) als GBS berechnete Grenzwert: 0,3 mg/m^3^ × Dichte 2,7 g/cm^3^ = 0,81 mg/m^3^ A. Gleiches gilt für jene schwerlöslichen Aluminiumverbindungen, deren Dichte in einem ähnlichen Bereich liegt (siehe Tabelle 1).

Zusammenfassend zeigt sich, dass eine Ableitung gemäß „Allgemeiner Staubgrenzwert (A-Fraktion) (Granuläre biobeständige Stäube (GBS))“ nicht erfolgen kann: 1) da im Urin der Ratte nach Inhalation der A-Fraktion Aluminium nachgewiesen (Tabelle 3) wurde, 2) systemische Effekte in Form von Neurotoxizität bei Untersuchungen am Arbeitsplatz auftreten und 3) da der GBS-Wert nicht die zusätzlichen Effekte der Aluminiumionen an der Lunge abdeckt (MAK-Wert von 0,05 mg/m^3^ A, errechneter GBS-Wert 0,81 mg/m^3^ A).

**E-Fraktion**

Für die Ableitung eines entsprechenden MAK-Werts fehlen Daten beim Menschen mit der E-Fraktion. Schwerlösliche Aluminiumverbindungen sind akut nicht haut- und augenreizend. Die Verweildauer im oberen Atemtrakt ist durch die mukoziliäre Clearance wesentlich geringer als in der Lunge. Eine Akkumulation und Reizwirkung im oberen Atemtrakt ist bei Konzentrationen unterhalb des allgemeinen Staubgrenzwerts von 4 mg/m^3^ für die E-Fraktion nicht anzunehmen. Die neurotoxische Wirkung wird durch Einhaltung des BAT-Werts vermieden. Die Korrelation zwischen Aluminium im Urin und in der Luft ist jedoch nicht belastbar, sodass aus dem BAT-Wert kein MAK-Wert abgeleitet werden kann.

Bei ca. 0,25–0,5 mg Al/m^3^ wurden keine neurotoxischen Wirkungen an Aluminium-Schweißern beobachtet (Buchta et al. 2003; Kiesswetter et al. 2009; Letzel et al. 2006).

Im Folgenden wird aufgezeigt, wie aus der NOAEC von 0,25–0,5 mg Al/m^3^ ein MAK-Wert für die E-Fraktion abgeleitet werden kann. Hierbei wird die konservative Annahme getroffen, dass diese NOAEC auch für die E-Fraktion gilt, obwohl in der Studie eine Exposition gegen Schweißrauch erfolgte, für den aufgrund der geringen Partikelgröße eine bessere Resorption unterstellt werden kann:

Die im oberen Atemtrakt deponierte E-Fraktion gelangt durch Abschlucken in den Magen-Darm-Trakt. Die orale Bioverfügbarkeit (<1 %) ist kleiner im Vergleich zur inhalativen Bioverfügbarkeit der A-Fraktion (2 %; siehe Abschnitt 3.1.1), sodass im Vergleich zur alveolengängigen Fraktion weniger systemisch verfügbar ist und damit auch der obere Wert der NOAEC von 0,5 mg/m^3^ als MAK-Wert für die E-Fraktion festgesetzt werden kann. Zusätzlich wird eine Plausibilitätsbetrachtung mit dem BAT-Wert vorgenommen:

Bei einer Konzentration von 0,5 mg Al/m^3^ und 10 m^3^ Atemvolumen werden bei 100 % inhalativer Deposition 5 mg Aluminium deponiert. Beim Worst-Case, einer maximalen 1%igen oralen Bioverfügbarkeit (siehe Abschnitt 3.1.2), entspricht dies 50 µg, die oral aufgenommen werden und systemisch wirken können. Diese Menge entspricht maximal der Menge, die bei Einhaltung des BAT-Wertes von 50 µg Al/g Kreatinin täglich ausgeschieden wird (tägliche Kreatininausscheidung 1–2,5 g (Manski 2022)). Der MAK-Wert für die E-Fraktion wird daher auf 0,5 mg Al/m^3^ festgesetzt.

Da am Arbeitsplatz Aluminiumverbindungen mit unterschiedlicher Löslichkeit vorkommen können, ist neben der Einhaltung des MAK-Wertes für A- und E-Fraktion auch auf die Einhaltung des BAT-Wertes (50 µg Al/g Kreatinin) zu achten.

**Spitzenbegrenzung. **Der MAK-Wert wurde aufgrund von Entzündungsreaktionen durch schwerlösliches Aluminiumoxyhydroxid in der Lunge abgeleitet.

Aluminium und schwerlösliche Aluminiumverbindungen sind akut nicht reizend. Sie werden deshalb der Spitzenbegrenzungs-Kategorie II zugeordnet.

Für die Akkumulation der schwerlöslichen Aluminiumverbindungen und die dadurch ausgelösten entzündlichen Effekte in der Lunge sowie für die neurotoxische Wirkung ist anzunehmen, dass dies durch das Konzentrations-Zeit-Produkt und nicht durch eine kurzzeitige höhere Spitzenkonzentration beeinflusst wird. Aufgrund der langen Halbwertszeit beim Menschen (Klotz et al. 2018) wird sowohl für die A-Fraktion, als auch für die E-Fraktion ein Überschreitungsfaktor von 8 festgesetzt.

**Fruchtschädigende Wirkung. **Mehrere epidemiologische Studien können aufgrund geringer Fallzahlen, fehlender Expositionserfassung, fehlender Kontrolle von Störvariablen (Confounder) oder anzunehmender Mischexposition nicht zur Bewertung herangezogen werden. Aluminiumhydroxid wird therapeutisch als Antazidum eingesetzt. Klinische Hinweise auf funktionelle Störungen im Zentralnervensystem und in den Nieren der Feten durch das aus den Antazida resorbierte Aluminium haben sich bisher nicht ergeben (Schaefer et al. 2011).

Es liegen keine Studien zur Entwicklungstoxizität nach inhalativer Exposition gegen Aluminium und seine schwerlöslichen Verbindungen vor. Nach oraler Verabreichung haben sich für **Aluminiumhydroxid**, der einzigen untersuchten Verbindung, NOAEL für pränatale Entwicklungstoxizität und Maternaltoxizität bei Ratten von 266 mg Aluminium/kg KG und Tag (Gomez et al. 1990, 1991; Greim 2007) und bei Mäusen von 100 mg Aluminium/kg KG und Tag (Colomina et al. 1992, 1994; Domingo et al. 1989; Greim 2007), den höchsten eingesetzten Dosierungen, ergeben.

Daten an Beschäftigten am Arbeitsplatz und Tierstudien mit inhalativer Gabe von schwerlöslichen Aluminiumverbindungen zeigen die systemische Verfügbarkeit von Aluminium an. Bei Berücksichtigung von ultrafeinem Aluminium und ultrafeinen Aluminiumverbindungen zeigt sich, dass orale Studien am Tier für die Bewertung der Aluminiumtoxizität am Arbeitsplatz nicht geeignet sind, weil es zu unterschiedlichen Aluminiumbelastungen in Organen nach oraler Verabreichung von ultrafeinem elementarem Aluminium sowie von ultrafeinem Aluminiumoxid im Vergleich zur inhalativen Exposition gegen Aluminiumoxyhydroxid kommt (siehe Abschnitt 3.1.6). Da keine Untersuchungen zur Entwicklungstoxizität mit inhalativer Applikation am Tier vorliegen, werden Aluminium und seine schwerlöslichen Verbindungen der Schwangerschaftsgruppe D zugeordnet.

**Krebserzeugende Wirkung. **Untersuchungen am Arbeitsplatz mit Exposition gegen schwerlösliche Aluminiumverbindungen lassen keinen Rückschluss auf eine kanzerogene Wirkung zu. Bewertungsrelevante Kanzerogenitätsstudien am Tier liegen nicht vor.

Für schwerlösliches **Aluminiumoxyhydroxid** zeigt sich in einer 28-Tage-Inhalationsstudien an männlichen Wistar-Ratten, dass ein Partikelüberladungseffekt auftritt (Pauluhn 2009 b, c; Bayer Schering Pharma AG 2008, 2009). Detailliert beschrieben ist der Wirkmechanismus der Partikelüberladung nach chronischer inhalativer Exposition gegen unlösliches **α-Aluminiumoxid** (Korund), was als GBS ohne stoffspezifische Toxizität zur Akkumulation von Partikeln in der Lunge sowie zur Beeinträchtigung der Lungenfunktion, zur Überlastung der Clearance, zu entzündlichen Veränderungen der Lunge, zur Fibrosierung und zur Tumorentstehung führen kann (Hartwig 2012; Hartwig und MAK Commission 2019).

Es kann angenommen werden, dass für schwerlösliche Aluminiumverbindungen ein Wirkungsmechanismus ähnlich dem von GBS vorliegt und der beobachtete Partikelüberladungseffekt in der Lunge zu Tumoren führen kann. Daher werden Aluminium und schwerlösliche Aluminiumverbindungen in Kategorie 4 für Kanzerogene eingestuft.

**Keimzellmutagene Wirkung. **Die In-vitro-Daten an Bakterien und Säugetierzellen und In-vivo-Daten an Drosophila zeigen keine Mutagenität. Eine DNA-schädigende Wirkung konnte für **ultrafeines elementares Aluminium** und **mikroskalige Aluminiumverbindungen** nur in vitro in Indikatortests gezeigt werden. **Ultrafeine Aluminiumverbindungen** zeigen neben validen negativen auch vereinzelt positive Ergebnisse in Indikatortests zur DNA-Schädigung, die in vivo in niedrigen und nachweislich nicht zytotoxischen Konzentrationen auftreten, in vitro bei gleichzeitig auftretender Zytotoxizität oder fehlender Konzentrations-Wirkungs-Beziehung. In vivo verursachten **ultrafeine Aluminiumverbindungen **zwar in einer Studie die Bildung von Mikronuklei und Chromosomenaberrationen bei hohen einmaligen Dosen (≥ 529 mg Al/kg KG), allerdings ohne eine ausreichende Charakterisierung der eingesetzten ultrafeinen Partikel. Zudem wiesen diese Partikel Verunreinigungen auf. Dieser positiven mit Mängeln behafteten Studie stehen mehrere negative Mikronukleustests gegenüber, darunter auch eine nach OECD-Prüfrichtlinie 474 durchgeführte Studie mit mehrfacher Gabe von Dosierungen bis 1059 mg Al/kg KG und Tag.

Zusammenfassend lässt sich für elementares Aluminium sowie schwerlösliche Aluminiumverbindungen zwar vereinzelt eine DNA-schädigende Wirkung in Indikatortests beobachten. Untersuchungen zur Bildung von Chromosomenaberrationen und Mikronuklei geben jedoch keine Hinweise auf eine direkte genotoxische Wirkung für elementares Aluminium und schwerlösliche Aluminiumverbindungen. Studien an Keimzellen liegen nicht vor. Daher werden elementares Aluminium und schwerlösliche Aluminiumverbindungen nicht in eine Kategorie für Keimzellmutagene eingestuft.

**Hautresorption. **Eine In-vitro-Studie mit Humanhaut ergab eine sehr geringe dermale Resorption im ng/cm^2^-Bereich nach 24-stündiger Applikation von **ultrafeinem Aluminiumoxid** (Mauro et al. 2019). Die dermale Aufnahme von schwerlöslichen Aluminiumverbindungen ist verglichen mit den löslichen Aluminiumverbindungen (Hartwig und MAK Commission 2025) als deutlich geringer anzunehmen. Die Aufnahme der löslichen Aluminiumverbindungen liegt bezogen auf den Referenzwert (BAR) weit im Hintergrundbereich und damit auch sehr weit unterhalb des BAT-Werts von 50 µg/g Kreatinin (Klotz et al. 2018). Die sehr geringe Aufnahme von Al über die Haut wird auch durch die Probandenstudie mit 14-tägiger Applikation eines **Aluminiumchlorhydrat**-Antitranspirants (Letzel et al. 2020) bestätigt. Aluminium und schwerlösliche Aluminiumverbindungen werden daher nicht mit „H“ markiert.

**Sensibilisierende Wirkung. **Da sich Metallallergien gegen die entsprechenden Ionen richten, sind Sensibilisierungen durch freigesetzte Ionen aus Aluminium und schwerlösliche Aluminiumverbindungen sehr unwahrscheinlich und dementsprechend liegen nur wenige Fälle vor, bei denen es zur Auslösung nach Kontakt mit schwerlöslichen Aluminiumverbindungen kam.

Tierexperimentelle Untersuchungen lieferten ebenfalls keine Hinweise auf eine sensibilisierende Wirkung schwerlöslicher Aluminiumverbindungen. Es erfolgt daher weiterhin keine Markierung mit „Sh“.

Zahlreiche Untersuchungen belegen zwar das Auftreten von Lungenerkrankungen nach massiver inhalativer Exposition gegen Aluminiumverbindungen. Eindeutige Ergebnisse, die auf eine atemwegssensibilisierende Wirkung hinweisen, finden sich in den vorliegenden Berichten jedoch nach wie vor nicht, sodass auch weiterhin keine Markierung mit „Sa“ erfolgt.
